# Engineering kinetics of TLR7/8 agonist release from bottlebrush prodrugs enables tumor-focused immune stimulation

**DOI:** 10.1126/sciadv.adg2239

**Published:** 2023-04-19

**Authors:** Sachin H. Bhagchandani, Farrukh Vohidov, Lauren E. Milling, Evelyn Yuzhou Tong, Christopher M. Brown, Michelle L. Ramseier, Bin Liu, Timothy B. Fessenden, Hung V.-T. Nguyen, Gavin R. Kiel, Lori Won, Robert S. Langer, Stefani Spranger, Alex K. Shalek, Darrell J. Irvine, Jeremiah A. Johnson

**Affiliations:** ^1^ Department of Chemistry, Massachusetts Institute of Technology, 77 Massachusetts Avenue, Cambridge, MA 02139, USA.; ^2^ Department of Chemical Engineering, Massachusetts Institute of Technology, 77 Massachusetts Avenue, Cambridge, MA 02139, USA.; ^3^ Koch Institute for Integrative Cancer Research, Massachusetts Institute of Technology, 500 Main Street, Cambridge, MA 02139, USA.; ^4^ Ragon Institute of MGH, MIT, and Harvard, Cambridge, MA 02139, USA.; ^5^ Department of Biological Engineering, Massachusetts Institute of Technology, 77 Massachusetts Avenue, Cambridge, MA 02139, USA.; ^6^ Institute for Medical Engineering and Science, Massachusetts Institute of Technology, 77 Massachusetts Avenue, Cambridge, MA 02139, USA.; ^7^ Broad Institute of MIT and Harvard, Cambridge, MA, 02142, USA.; ^8^ Department of Biology, Massachusetts Institute of Technology, 77 Massachusetts Avenue, Cambridge, MA 02139, USA.; ^9^ Howard Hughes Medical Institute, Chevy Chase, MD 20815, USA.

## Abstract

Imidazoquinolines (IMDs), such as resiquimod (R848), are of great interest as potential cancer immunotherapies because of their ability to activate Toll-like receptor 7 (TLR7) and/or TLR8 on innate immune cells. Nevertheless, intravenous administration of IMDs causes severe immune-related toxicities, and attempts to improve their tissue-selective exposure while minimizing acute systemic inflammation have proven difficult. Here, using a library of R848 “bottlebrush prodrugs” (BPDs) that differ only by their R848 release kinetics, we explore how the timing of R848 exposure affects immune stimulation in vitro and in vivo. These studies led to the discovery of R848-BPDs that exhibit optimal activation kinetics to achieve potent stimulation of myeloid cells in tumors and substantial reductions in tumor growth following systemic administration in mouse syngeneic tumor models without any observable systemic toxicity. These results suggest that release kinetics can be tuned at the molecular level to provide safe yet effective systemically administered immunostimulant prodrugs for next-generation cancer immunotherapies.

## Teaser

Molecularly tuned linkers in bottlebrush prodrugs allow safe, systemic delivery of immune agonists to boost anti-tumor responses.

## INTRODUCTION

Therapies rooted in modulating the immune system have played a crucial role in the treatment of infectious diseases and, more recently, have paved the way for a paradigm shift toward redefining cancer treatment. Cancer immunotherapies, such as antibodies blocking programmed cell death protein 1 (PD-1) and programmed cell death-ligand 1 (PD-L1) interactions, have demonstrated clinical activity in diverse oncological indications (*1*). Most patients, however, still do not respond to currently available immunotherapies, possibly because of an inability to generate potent cytotoxic T lymphocyte (CTL) responses against tumor neoantigens as well as the immunosuppressive and tolerizing effects of the tumor microenvironment (*2*–*4*). In preclinical models of immunotherapy-resistant tumors, antigens derived from dying tumor cells are often not processed efficiently by myeloid cells such as dendritic cells (DCs), monocytes, and macrophages due to a lack of activation and costimulatory signals that are necessary for presentation of tumor-derived peptides to T cells (*5*–*8*). Thus, shifting the myeloid activation state by promoting inflammation in the tumor microenvironment through innate immune stimulation has the potential to play an important role in reversing tumor microenvironment immunosuppression (*9*–*11*).

Therapeutic activation of Toll-like receptors (TLRs) is one of the most extensively studied approaches to activate myeloid cells, trigger acute inflammation, and stimulate adaptive immunity in tumors (*12*, *13*). In particular, targeting TLR7/8 has been shown to be very effective at eliciting acute inflammation: Activation of TLR7 expressed by plasmacytoid DCs leads to production of type 1 interferons (IFNs), while TLR8 is expressed by conventional DCs, monocytes, and macrophages, and its activation triggers the production of interleukin-12 (IL-12) and other proinflammatory cytokines (*14*). A large body of work has focused on the development of synthetic ligands for TLR7/8 following the discovery that the antiviral properties of imidazoquinolines (IMDs) were mediated by activation of these TLRs (*15*). One of the first compounds in this class, imiquimod (R837), showed promising results in clinical trials for topical treatment of skin malignancies such as basal cell carcinoma and received Food and Drug Administration (FDA) approval in 2004 (*16*, *17*). This success led to the development of more potent IMDs such as resiquimod (R848) and 852-A for systemic treatment of metastatic cancers. Intravenous administration of these compounds in patients with solid tumors (colon, breast, ovarian, and cervical cancers) showed promising results in terms of disease stabilization in a proportion of patients and increased levels of type I IFNs and proinflammatory cytokines such as tumor necrosis factor–α (TNF-α) and IFN-γ, indicating potent immune activation (*18*, *19*); however, patients receiving these compounds experienced severe side effects because of systemic cytokine induction, ultimately leading to premature discontinuation of clinical trials. The prevalence of severe immune-related adverse events (irAEs) due to systemic exposure has presented a major bottleneck in expanding the use of IMDs and other innate immune stimulators (*20*). Thus, strategies to focus the action of IMDs in the tumor microenvironment are needed to unlock the potential of these TLR agonists for metastatic disease.

One strategy to bias the biodistribution of IMDs toward tumors involves their physical encapsulation in micro- and nanoparticulate carriers (*21*); however, to date, the reported formulations have had relatively short (<48 hours) release half-lives that result in systemic toxicity (*22*–*25*). IMDs have also been chemically conjugated to spherical “nanogel” scaffolds (~50 nm diameter) (*26*) or aggregates of linear polymers (~700 nm diameter) (*27*) to enhance their innate immune cell uptake and retention in lymph nodes following subcutaneous injection. Similarly, they have been conjugated to polyethylene glycol (PEG) to form large vesicles (~200 nm diameter) designed to release active TLR7 agonists in endosomes in response to β-glucuronidase and esterase-mediated release (*28*). Nevertheless, while these strategies have suggested a benefit of using nanoscale particle structures to improve the activity of TLR7 agonists following subcutaneous dosing as vaccine adjuvants, they have not been tested in the context of intravenous administration, the preferred approach for treatment of metastatic cancers where systemic toxicities are more challenging to overcome. In the setting of systemic administration, self-aggregating nanoparticles (~20 nm) based on peptides linked to a charge-modifying group and IMDs conjugated to a hydrophobic block showed no therapeutic efficacy in the absence of a tumor neoantigen when injected intravenously in the treatment of syngeneic mouse tumors (*29*). This finding highlights the challenges associated with obtaining effective tumor-focused innate immune activation upon systemic treatment with IMDs. The field to date lacks a thorough understanding of how the kinetics of systemic versus tumor exposure to IMDs affects the interplay between safety and efficacy, which has led to limited efficacy of TLR agonists as monotherapies in preclinical research and has hindered the clinical progress of IMDs.

We hypothesized that IMD prodrugs with appropriate activation kinetics could hold the key to enabling tumor-specific immune activation. Specifically, by aligning the timing of tumor accumulation with prodrug activation in the tumor microenvironment following systemic administration, it could be possible to substantially expand the therapeutic index of these TLR agonists. Isolating activation kinetics as a key variable, however, requires a prodrug platform capable of generating multiple physically equivalent TLR agonist prodrugs that only vary by their activation kinetics, which is traditionally very challenging and has, so far, not been explored in the context of immune stimulation. We have created “bottlebrush prodrugs” (BPDs), which are a class of polymers that feature drug molecules covalently linked along their rigid backbones with PEG chains that extend from the backbone to shield the drugs from surface exposure (*30*–*32*). These structures offer unique advantages for controlled delivery, particularly for studying the impact of release kinetics on biological functions, as they can be easily manufactured with a variety of prodrug linkers while maintaining identical sizes, shapes, biodistribution, serum pharmacokinetics (PKs), and other key physical properties. Given the enormous interest in controlled immune stimulation, we surmised that the BPD platform would offer a unique avenue to isolate IMD prodrug activation kinetics as a variable.

Here, we report the design, synthesis, and detailed physical and immunological characterization of a family of “R848-BPDs” that only differ by their rate of release of R848. These R848-BPDs have antibody-like sizes (~10 nm) and consistent, narrow size distributions that do not vary as a function of prodrug linker. By carefully tuning the molecular structures of these linkers, R848 release was varied over an order of magnitude, which enabled the correlation of R848 prodrug activation kinetics with a range of safety and efficacy biomarkers, leading to the discovery of optimal R848-BPDs that were both safe for systemic administration and resulted in potent tumor inhibition in multiple tumor models with no signs of systemic toxicity. We show that the antitumor efficacy of R848-BPDs was mediated by sustained myeloid activation and reprogramming in the tumor microenvironment and draining lymph nodes. Together, this work provides a new category of safe and effective TLR agonist prodrugs for potential clinical translation and reveals that tissue exposure kinetics can be leveraged to optimize immune stimulation, which may apply to other small-molecule innate immunomodulators such as stimulator of interferon genes (STING) agonists, retinoic acid-inducible gene I (RIG-I) agonists, and other TLR agonists in the future.

## RESULTS

### Synthesis and characterization of a library of R848-BPDs with varying linkers

**Fig. 1.**
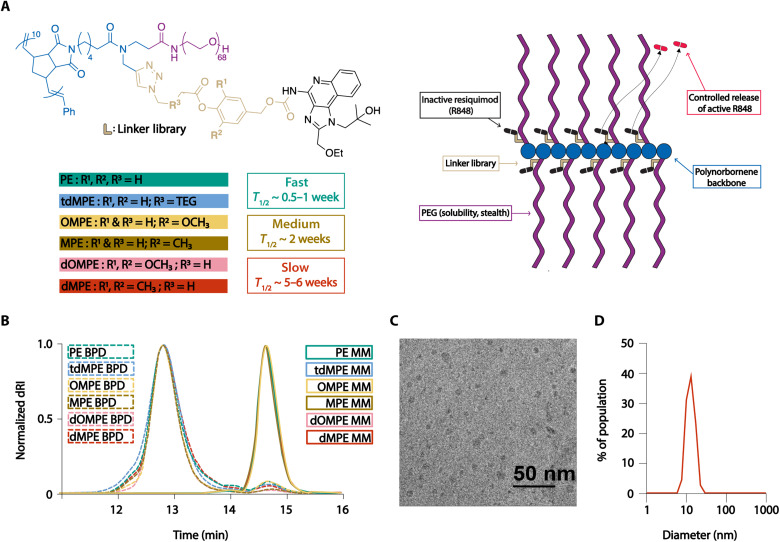
Development and characterization of the R848-BPD library. (**A**) Schematic and chemical structure of the R848-BPD library composed of “fast,” “medium,” and “slow” prodrug activation kinetics. (**B**) Size exclusion chromatography traces of R848-MMs and R848-BPDs show that all of the R848-BPDs have consistent sizes independent of their linker composition. (**C**) Example cryo-TEM image of slow dMPE R848-BPD. Scale bar, 50 nm. (**D**) Hydrodynamic diameter of medium MPE R848-BPD as measured by DLS.

The R848-BPD synthesis workflow is summarized schematically in Fig. 1A. Macromonomers (MMs) bearing an exo-norbornene imide polymerizable group bound to 3-kDa PEG and one of six azido-R848 ester-based prodrugs were prepared through copper-catalyzed azide-alkyne cycloaddition "click" chemistry (figs. S1 to S7). Each of these six MMs (10 equivalents) was subjected to ring-opening metathesis polymerization using a Grubbs third-generation initiator (G3; 1 equivalent) to provide six R848-BPDs with theoretical backbone degrees of polymerization of 10. The resulting BPDs showed expected overlapping profiles by size exclusion chromatography and had identical sizes and morphologies as assessed by dynamic light scattering (DLS) and cryo–transmission electron microscopy (cryo-TEM) (Fig. 1, B to D; table S1; and fig. S8); thus, their sizes are the same, and any differences in their biological function can be attributed to their prodrug activation kinetics. The small-molecule prodrug precursors and R848-MMs were also characterized using ^1^H and ^13^C nuclear magnetic resonance spectroscopy and mass spectrometry where appropriate (figs. S1 to S7).

### R848-BPDs display tunable release characteristics ranging from a few days to several weeks

**Fig. 2.**
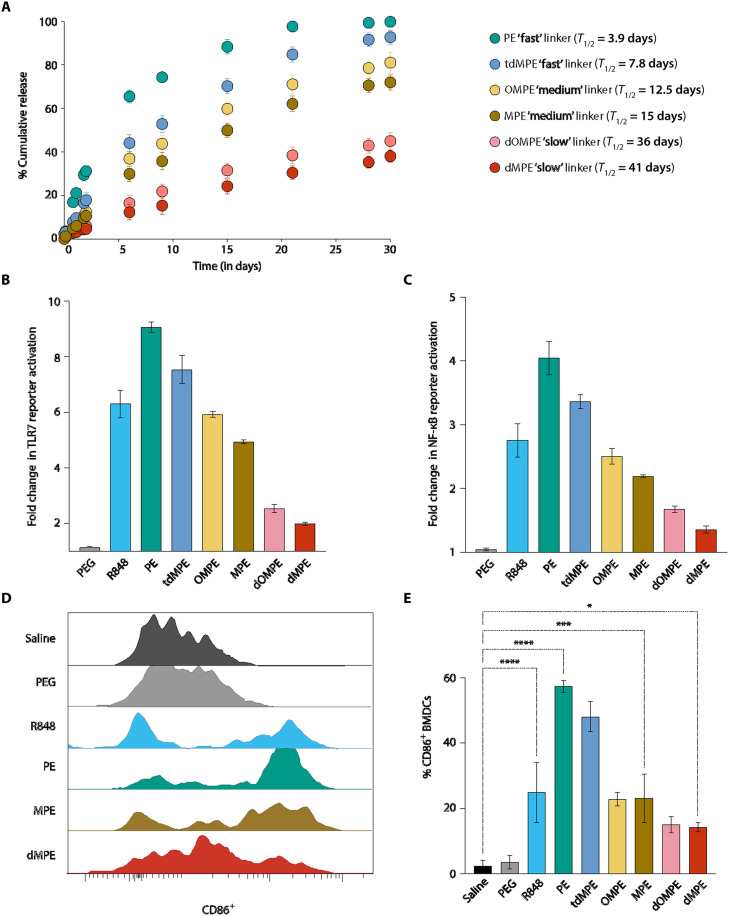
In vitro release studies and immune activation analysis. (**A**) Release of free R848 from R848-MMs of varied linker structure in neutral PBS buffer. (**B**) R848 activity in R848-BPDs measuring fold change (relative to saline) in TLR7 reporter activation in HEK-human-TLR7 reporter cells 48 hours after incubation, (**C**) NF-kB reporter activation (relative to saline) in mouse NF-κB reporter cells 48 hours after activation, and (**D**) representative histograms and (**E**) quantification of % CD86 expression in mouse bone marrow–derived DCs (BMDCs) upon treatment with saline, a PEG bottlebrush lacking R848, free R848, or R848-BPDs for 48 hours. Data are presented as mean values ± SEM with *n* = 3 independent samples for each group tested. Statistical comparisons in (E) were tested using one-way analysis of variance (ANOVA) followed by Dunnett’s multiple-comparisons test. *P < 0.05, ***P < 0.001, and ****P < 0.0001.

Given that the R848-BPDs described here have very similar sizes, shapes, compositions, and active drug mass fractions (~10%), their safety and efficacy should be a function of the R848 aryl ester linker cleavage rate, which is a function of hydrolytic and esterase cleavage activity in vivo (*30*). The aryl ester–based linkers were molecularly tuned to achieve release half-lives that span an order of magnitude in neutral buffer and accelerated release in the presence of model esterases, because these enzymes are expressed in endolysosomes and are often overexpressed in the tumor microenvironment (Fig. 2A and fig. S9, A to C) (*28*, *33*, *34*). To assess the rate of hydrolytic cleavage under model conditions, each R848-MM was dissolved in pH 7.4 phosphate-buffered saline (PBS) buffer at 37°C; samples were taken over time to quantify the amount of free R848 by liquid chromatography–mass spectrometry (LC-MS). Variations in R848 release half-life (*T*_1/2_) values from ~4 to ~40 days were observed (Fig. 2A), which correlated with the local steric and electronic environment of each aryl ester linker as defined by the substituents R^1^ and R^2^ as well as the composition of the linear carbonyl component (R^3^). For example, the fastest release was observed when R^1^ and R^2^ = H, the smallest substituents investigated (sample PE; *T*_1/2_ ~ 3.9 days; table S2). Increasing the steric hindrance and electron density of R^1^ and R^2^ markedly slows hydrolysis: When one (MPE) or two (dMPE) *ortho-*methyl groups are introduced, *T*_1/2_ increases to 15 and 41 days, respectively (table S2). Similar effects were observed for less-bulky yet more electron-donating methoxy groups: When one (OMPE) or two (dOMPE) *ortho-*methoxy groups were introduced, *T*_1/2_ values were 12.5 and 36 days, respectively (Fig. 2A and table S2). Last, the steric and electronic effects of methyl substitution at R^1^ and R^2^ could be mitigated through the introduction of a more hydrophilic triethylene glycol at R^3^ (tdMPE; *T*_1/2_ ~ 7.8 days; table S2). Together, these six R848-BPDs are divided into “slow” (MPE and OMPE), “medium” (dMPE and dOMPE), and “fast” (PE and tdMPE) release in subsequent discussion based on these measured hydrolysis kinetics. Furthermore, we conducted release studies using the “medium” and “slow” BPDs in the presence of a model esterase (*T. lanuginosus *lipase). As expected, the presence of esterase substantially increased the release rate of R848 from the BPDs, and the rates of release followed the same trend as observed for the background hydrolysis reaction (i.e., medium releases faster than slow; fig. S9C). Last, we note that, while ester cleavage is rate limiting in this system, release of free R848 requires subsequent 1,6-elimination of a *para-*quinone methide derivative and expulsion of one molecule of CO_2_ from the resulting carbonic acid. Under the conditions used for these release studies, we have never observed either the phenolic or carbonic acid intermediates, suggesting that these steps are very fast compared to ester cleavage. Moreover, *para*-quinone methides are hydrolyzed rapidly under aqueous conditions to generate benign alcohols as products (*35*).

Next, we sought to correlate these MM prodrug activation kinetics with the activity of each R848-BPD in vitro using TLR7 and nuclear factor κB (NF-κB) reporter cell lines (both human and mouse). In support of our hypothesis, the R848-BPDs showed linker-dependent activation of both cell types compared to saline control that directly correlated with the measured half-lives of MM prodrug activation (*36*). As shown in Fig. 2B, incubation of fast R848-BPDs with human TLR7 reporter cells for 48 hours triggered greater TLR activation than free R848, perhaps because of greater cell uptake of the BPD into endosomes where TLR7 is expressed. Notably, the medium and slow R848-BPDs displayed similar or lower activation compared to R848, as expected if the TLR agonist is inactive until released from the BPD scaffold (Fig. 2B and fig. S10A). Furthermore, the BPD backbone with no R848 (“PEG”) showed minimal in vitro activation relative to saline (Fig. 2B and fig. S10A). Last, we note that there were no direct cytotoxic effects of R848-BPDs on human embryonic kidney (HEK) 293 cells when incubated in vitro for up to 48 hours (fig. S10B). Similar patterns of cellular activation were measured using NF-κB reporter cells (Fig. 2C). When R848-BPDs were incubated with primary mouse bone marrow–derived DCs (BMDCs) or control conditions for 48 hours, up-regulation of activation marker CD86, as determined by flow cytometry (*37*), also correlated with linker composition. For example, fast, medium, and slow R848-BPDs up-regulated CD86 expression in ~60, ~25, and ~10% of BMDCs, respectively (Fig. 2, D and E). Together, these results demonstrate that the release kinetics of R848-BPDs can be tuned through molecular design and that these kinetics directly correlate with TLR activation in vitro, yielding increased (for fast), similar (for medium), and decreased (for slow) activation compared to R848 alone.

### R848-BPDs with optimal linkers have greater maximum tolerated doses and broad therapeutic windows compared to free R848

**Fig. 3.**
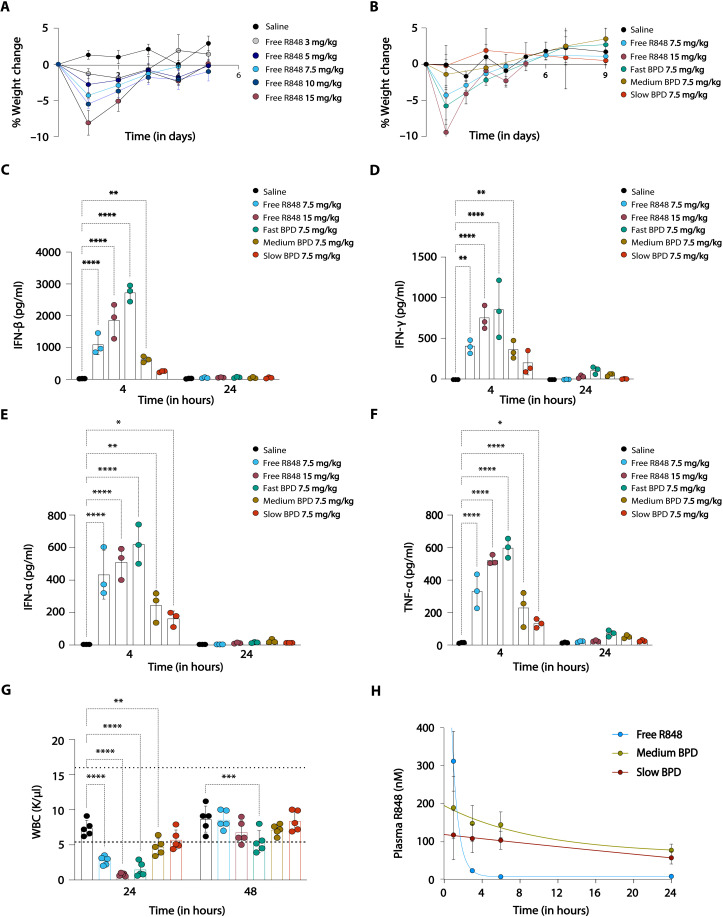
MTD and toxicity assessments of free R848 and R848-BPDs. (**A**) Weight loss measurements for C57BL/6 mice (*n* = 3 per group) following intravenous administration of increasing concentrations of free R848 to define the MTD as 7.5 mg/kg. (**B**) Weight loss measurements for C57BL/6 mice (*n* = 3 per group) following intravenous administration of free R848 (7.5 and 15 mg/kg) and R848-BPDs (7.5 mg/kg). (**C** to **F**) Serum cytokine measurements taken at 4 and 24 hours postinjection of free R848 (7.5 and 15 mg/kg) and R848-BPDs (7.5 mg/kg). (**G**) WBC count taken at 24 and 48 hours postinjection of free R848 (7.5 and 15 mg/kg) and R848-BPDs (7.5 mg/kg). (**H**) Pharmacokinetic (PK) analysis of plasma R848 at 1, 3, 6, and 24 hours postinjection of free R848 (7.5 mg/kg) and medium and slow R848-BPDs (7.5 mg/kg) in C57BL/6 mice (*n* = 5 per group). Data are presented as mean values ± SEM with *n* = 3 or 5 independent samples for each group tested. Statistical comparisons in (C) to (F) and (G) were tested using two-way ANOVA followed by Dunnett’s multiple-comparisons test. *P < 0.05, **P < 0.01, ***P < 0.001, and ****P < 0.0001.

The maximum tolerable dose (MTD) of free R848 was determined in healthy C57BL/6J mice by monitoring weight loss and clinical signs (body condition score measurements) over a period of 1 week following a single dose (*38*). Mice dosed at 10 and 15 mg/kg demonstrated visible signs of restricted movement and lethargy in addition to weight loss greater than 5% of the original body weight (Fig. 3A); thus, 7.5 mg/kg was established as the MTD. We then evaluated three different R848-BPDs (“fast BPD,” PE linker; “medium BPD,” MPE linker; “slow BPD,” dMPE linker) at doses of 75, 150, 300, 600, and 750 mg/kg, which, given the ~10% R848 loading of the BPDs, correspond to 1, 2, 4, 8, and 10× the MTD (7.5 mg/kg) of free R848, respectively (Fig. 3B and figs. S11 to S13). Notably, while the “fast PE” BPD had a similar MTD compared to free R848 when normalized by the mass of R848, the “medium MPE” and “slow dMP”’ BPDs exhibited MTDs at four and eight times higher R848 concentrations, respectively (figs. S11 to S13). Serum was collected 4, 24, and 48 hours postinjection and was assayed for cytokines commonly released downstream of TLR7/8 activation; linker-dependent increases in type I IFNs (both IFN-α and IFN-β) and proinflammatory cytokines such as IFN-γ, IL-6, monocyte chemoattractant protein 1 (MCP-1), and TNF-α were observed (Fig. 3, C to F). While the fast PE BPDs induced even higher levels of systemic cytokine production (greater than or equal to two times MTD of free R848), both the medium MPE and slow dMPE BPDs induced lower serum cytokine levels when given at equivalent R848 dosing levels (Fig. 3, C to F). Because these BPDs have identical physical properties and only differ by their release rate, these differences must arise from R848 exposure kinetics and not differences in, e.g., distribution/accumulation. Moreover, this reduced toxicity of medium and slow BPDs was not simply reflecting delayed activation of cytokine production by the slowed release kinetics, as cytokine levels remained low at 24 and 48 hours (Fig. 3, C to F, and fig. S14). Whole blood was collected in the different groups and subjected to a blood panel analysis wherein the white blood cell (WBC) count served as a proxy for drug concentration in the blood, because R848 induces lymphocytes to leave blood transiently in a dose-dependent manner (*39*). Free R848 and fast PE showed a lowered WBC count, whereas medium and slow BPDs remained within limits of the normal range (Fig. 3G). Acute toxicities were also assessed via liver enzyme biomarkers (alanine aminotransferase and aspartate aminotransferase) and blood urea nitrogen as surrogates for liver and kidney function, respectively. In all cases, the medium and slow BPDs consistently showed normal levels of the relevant biomarkers (as opposed to free R848) even in the early time points (acute phase), suggesting that they are safer than the fast BPD or R848 alone due to their delayed release kinetics (fig. S14). To confirm that the medium and slow BPDs led to reduced systemic R848 exposure, we analyzed the serum PKs of R848 following dosing of free R848 or the R848-BPDs. The medium and slow BPDs showed lower initial free drug concentrations in serum compared to free R848, which display a peak at 30 min followed by clearance in a few hours (Fig. 3H and table S3).

### R848-BPDs demonstrate tumor accumulation and uptake by innate immune cells in the tumor and tumor-draining lymph nodes

**Fig. 4.**
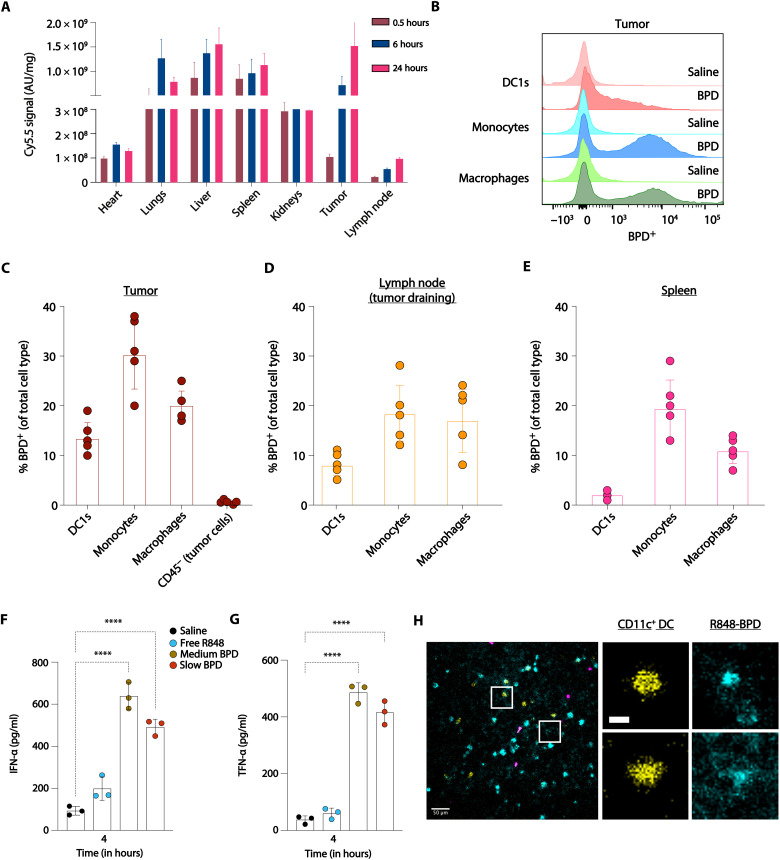
Organ and cellular biodistribution and pharmacodynamics of R848-BPDs. (**A**) Organ biodistribution in digested tissues (means ± SEM) of Cy5.5 fluorescently labeled medium R848-BPDs (7.5 mg/kg) injected in C57BL/6 mice (*n* = 5 per group) bearing syngeneic MC38 colon carcinoma tumors (~25 mm^2^). (**B**) Representative flow cytometry histograms and (**C** to **E**) cellular biodistribution data of Cy5.5-labeled medium R848-BPDs taken 24 hours postinjection in C57BL/6 mice (*n* = 5 per group) bearing syngeneic MC38 colon carcinoma tumors (~25 mm^2^), indicating uptake by myeloid cells (DC1s, monocytes, and macrophages) in the tumor, tumor-draining lymph node, and spleen. (**F** and **G**) Tumor cytokine measurements taken at 4 hours postinjection of free R848, medium, and slow R848-BPDs (7.5 mg/kg) in C57BL/6 mice (*n* = 3 per group) bearing syngeneic MC38 colon carcinoma tumors (~25 mm^2^). (**H**) Image from intravital imaging showing R848-BPD (in cyan) colocalized with CD11c^+^ DCs (yellow) and other broader myeloid populations (magenta). Scale bar, 50 μm. Insets highlighting colocalization of R848-BPD (cyan) on CD11c^+^ DCs (yellow) in separate channels. Scale bar, 10 μm. Statistical comparisons in (F) and (G) were tested using an ANOVA followed by Dunnett’s multiple-comparisons test. ****P < 0.0001. AU, arbitrary units.

On the basis of its promising pharmacodynamic activity, we next evaluated the biodistribution of fluorescently labeled R848-BPDs prepared with the medium release rate MPE linker following intravenous injection in mice bearing syngeneic MC38 colon carcinoma tumors. BPDs, by virtue of their small size and nonspherical shape, have been shown to display effective accumulation and penetration into subcutaneous and orthotopic murine tumors, with liver, spleen, and lungs as other major clearance tissues (*30*). Here, the R848-BPD performed similarly, with the highest levels of tissue accumulation in tumors and the liver (Fig. 4A). The BPD continued to increase in accumulation in the tumor through 24 hours, and similar accumulation was observed in the tumor-draining lymph node albeit at much lower levels (Fig. 4A). Flow cytometry analysis of the tumor and tumor-draining lymph nodes revealed uptake of the R848-BPDs by innate immune cells, including conventional type 1 DCs (cDC1s), monocytes, and macrophages (Fig. 4, B and C). Approximately 20% of macrophages in the tumor were positive for R848-BPDs, along with 30% of monocytes and 15% of cDC1s (Fig. 4, B to E). When we treated tumor-bearing mice with free R848, slow R848-BPDs, or medium R848-BPDs, we found that the substantial tumor accumulation of BPDs correlated with the generation of high levels of proinflammatory cytokines including type I IFN and TNF-α in the tumor microenvironment, which were not induced to statistically significant levels over saline-treated tumors by free R848 (Fig. 4, F and G). Last, we verified this cell uptake in the tumor microenvironment via intravital fluorescence imaging, showing R848-BPDs (cyan) colocalized with CD11c^+^ DCs (yellow) and other myeloid cells (magenta) (Fig. 4H).

### Systemically administered R848-BPDs elicit therapeutic responses both as monotherapies and in combination with checkpoint blockade therapy in mouse models of colon carcinoma

**Fig. 5.**
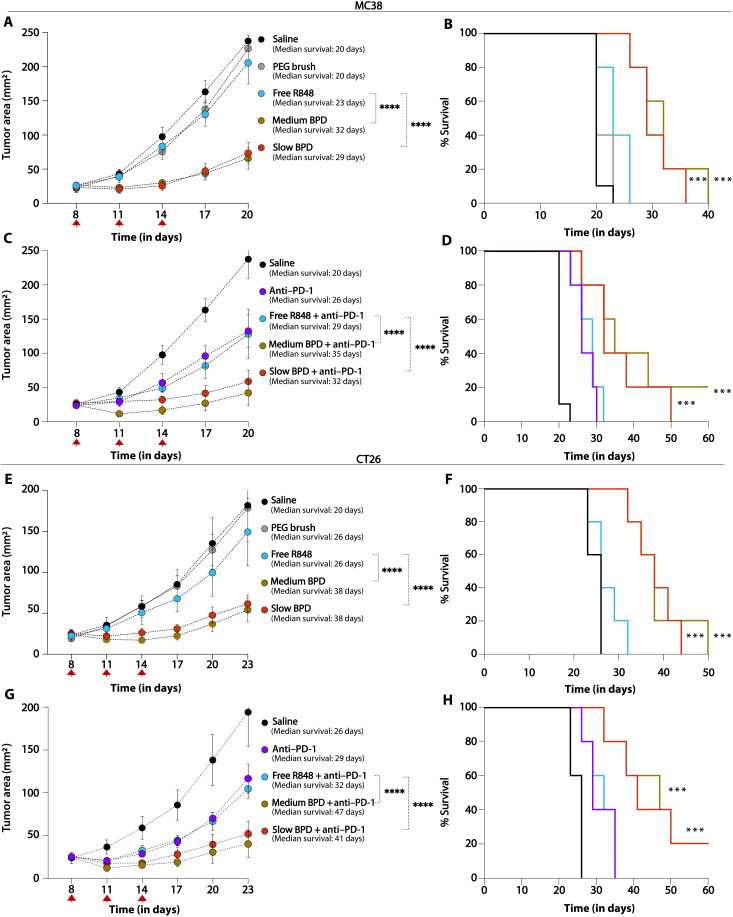
In vivo efficacy studies with medium and slow R848-BPDs in MC38 and CT26 colon carcinoma models demonstrated lower tumor burden and improved survival rates compared to control groups. (**A**) Tumor size (means ± SEM) and (**B**) Kaplan-Meier survival curves for C57BL/6 mice (*n* = 5 per group) bearing syngeneic MC38 colon carcinoma tumors injected retro-orbitally with R848-BPDs (medium and slow) along with controls. (**C**) Tumor size (means ± SEM) and (**D**) Kaplan-Meier survival curves for C57BL/6 mice (*n* = 5 per group) bearing syngeneic MC38 colon carcinoma tumors injected retro-orbitally with R848-BPDs (medium and slow) in combination with anti–PD-1 checkpoint blockade along with controls. (**E**) Tumor size (means ± SEM) and (**F**) Kaplan-Meier survival curves for BALB/c mice (*n* = 10 per group) bearing syngeneic CT26 colon carcinoma tumors injected retro-orbitally with R848-BPDs (medium and slow) along with controls. (**G**) Tumor size (means ± SEM) and (**H**) Kaplan-Meier survival curves for BALB/c mice (*n* = 10 per group) bearing syngeneic CT26 colon carcinoma tumors injected retro-orbitally with R848-BPDs (medium and slow) in combination with anti–PD-1 checkpoint blockade along with controls. Statistical comparisons among tumor areas in (A) to (H) were performed using two-way ANOVA with Dunnett’s multiple-comparisons test, and survival curves in (A) to (H) were compared using a log-rank (Mantel-Cox) test. ***P < 0.001 and ****P < 0.0001.

Given these promising biodistribution results, the therapeutic efficacy of R848-BPDs was evaluated in mice bearing subcutaneous MC38 tumors (*n* = 5). For these studies, we focused on the medium and slow BPDs given their promising safety profile compared to the fast BPD. These two BPDs along with free R848 were administered intravenously at 7.5 mg/kg to the mice once every 3 days for three total doses. Notably, the R848-BPDs displayed a substantial slowing of tumor progression (*P* < 0.0001) and improved overall survival compared to mice given free R848 (Fig. 5, A and B) with no signs of toxicity (fig. S15A). Together with the safety results described above, these data further highlight the importance of R848 release kinetics in achieving simultaneous safety and efficacy in vivo.

As noted above, improved innate immune stimulation by TLR7/8 activation could boost responses to immunotherapies such as immune checkpoint blockade (*40*). Thus, following these monotherapy studies, we next performed combination multidose in vivo efficacy studies where mice bearing MC38 tumors were treated with R848-BPDs (MPE or dMPE) in combination with anti–PD-1 (200 μg). Animals treated with R848-BPDs combined with anti–PD-1 showed better tumor control (*P* < 0.0001) and longer-term survival compared to mice given free R848 in combination with anti–PD-1 or anti–PD-1 alone (Fig. 5, C and D).

To validate the generality of these treatment responses, we evaluated the medium and slow R848-BPDs in BALB/c mice bearing CT-26 colon carcinoma tumors (*n* = 10) with the same dosing pattern. Here, the R848-BPDs both delayed tumor outgrowth (*P* < 0.0001) and significantly increased median survival compared to untreated tumors with no signs of toxicity, while free R848 had no significant impact on tumor growth or overall survival (Fig. 5, E and F, and fig. S15B). Last, combining R848-BPDs with anti–PD-1 in the CT-26 model further boosted the response, extending survival and leading to complete responses in 20% of the animals (Fig. 5, G and H).

### R848-BPDs exert antitumor effects via sustained activation of innate immune cell subsets

**Fig. 6.**
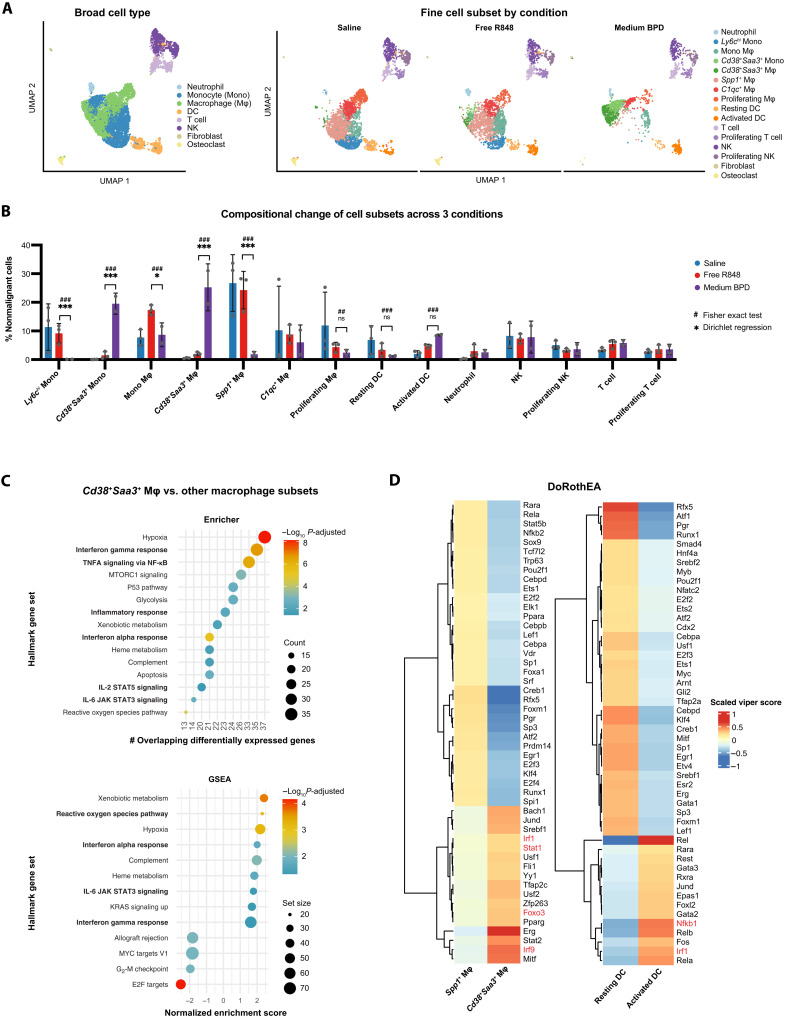
scRNA-seq of MC38 tumors 24 hours posttreatment with saline, free R848, and medium R848-BPD. (**A**) Uniform manifold approximation and projection (UMAP) visualization of nonmalignant cells colored by eight broad cell types (left) and by 16 fine cell subsets separated by experimental conditions (right). NK, natural killer. (**B**) Difference in nonmalignant cell proportional composition across the three experimental conditions. Adjusted *P* values are only shown for cell subset comparisons (medium BPD versus free R848) that are significant by at least one statistical test: Fisher exact test (shown in #) or Dirichlet regression analysis (shown in *). (**C**) Enricher (top) and GSEA (bottom) pathway enrichments for the differentially expressed genes in *Cd38^+^Saa3^+^* macrophages compared to the rest of the macrophage subtypes, showing up-regulations of inflammatory response, IFN, and reactive oxygen species pathways (in boldface). (**D**) DoRothEA transcription factor (TF) inference for *Spp1^+^* macrophages versus *Cd38^+^Saa3^+^* macrophages and for DCs versus activated DCs, suggesting inflammation-related TF activities (colored in red) in *Cd38^+^Saa3^+^* macrophages and activated DCs. Only TF-target interactions with a high confidence level were selected for this analysis. ns, not significant. *P < 0.05, ***P < 0.001, ^##^*P* < 0.01, and ^###^*P* < 0.001.

Having shown that R848-BPDs display promising capability to control tumor growth and improve survival in murine tumor models, we next sought to examine whether their mechanism of action is due to immune stimulation via TLR activation because R848 canonically activates TLR7/8 expressed by myeloid cells. To determine which cell types contribute to the observed antitumor effects of the R848-BPDs, we performed single-cell RNA sequencing (scRNA-seq) on tumors 24 hours after dosing with either saline (*n* = 3), free R848 (*n* = 3), or medium R848-BPDs (*n* = 2). After removing MC38 tumor cells (fig. S16A), we analyzed 8017 nonmalignant cells, resolving eight broad cell types and 16 fine cell subsets across three conditions (Fig. 6A and fig. S16B). First, we tested for changes in the relative frequencies of cell subsets across conditions. To account for compositional dependencies among cell subsets within each tumor, we conducted both Fisher exact tests and multivariate Dirichlet regression analysis (*41*). This analysis revealed distinct innate cell subsets enriched by the R848-BPDs: *Cd38^+^Saa3^+^* monocytes and *Cd38^+^Saa3^+^* macrophages [Dirichlet adjusted P value (*P*_adj_) = 7.55 × 10^−6^ and Fisher *P*_adj_ = 2.33 × 10^−101^, as well as Dirichlet *P*_adj_ = 9.28 × 10^−6^ and Fisher *P*_adj_ = 1.61 × 10^−124^, respectively], in tandem with decreases in *Ly6c*^hi^ monocytes and *Spp1^+^* macrophages (Dirichlet *P*_adj_ = 1.33 × 10^−5^ and Fisher *P*_adj_ = 9.29 × 10^−53^, as well as Dirichlet *P*_adj_ = 4.12 × 10^−6^ and Fisher *P*_adj_ = 1.55 × 10^−119^, respectively) when comparing R848-BPDs to free R848 (Fig. 6B and table S4). Furthermore, when we performed differential expression analyses within each subset, we found that R848-BPD therapy induced distinct expression profiles in macrophages, monocytes, and cDC1s, but not tumor-infiltrating lymphocytes (fig. S16C and table S4). Gene set enrichment analyses (GSEA) over differentially expressed genes showed that *Cd38^+^Saa3^+^* macrophages had higher enrichment scores for pathways related to inflammatory response, IFN, and reactive oxygen species compared to all other macrophage subsets (Fig. 6C). Moreover, transcription factor (TF) inference analysis using DoRothEA confirmed that *Cd38^+^Saa3^+^* macrophages had higher activities of inflammation-related TFs, such as Irf1, Irf9, Stat1, and Foxo3, compared to the *Spp1^+^* macrophages previously reported to be immunosuppressive with protumorigenic roles (Fig. 6D) (*42*, *43*). Relatedly, as a nicotinamide adenine dinucleotide (NAD)–consuming enzyme, CD38 has been shown to be up-regulated in M1 macrophages in both mice and humans upon acute inflammation, suggesting an antitumor proinflammatory effect of R848-BPDs in macrophages (*44*).

Similarly, we identified an activated DC population, exhibiting high expression of *Ccr7*, *Ccl22*, *Ccl5*, *Cxcl16*, and *Il12b* (Fig. 6B and fig. S16, B and D) and strong inflammation-related TF activation, including Irf1 and Nfkb1 (Fig. 6E), akin to previously reported tumor-infiltrating CCR7^+^ DCs that secrete IL-12 and activate T cells to increase antitumor activity (*45*). While resting and activated DC populations were present in all experimental conditions, activated DCs were modestly enriched upon R848-BPD treatment (Fisher *P*_adj_ = 6.96 × 10^−8^ when comparing R848-BPDs to free R848; not significant by multivariate Dirichlet regression analysis). Collectively, our data suggest that R848-BPDs stimulate monocytes, macrophages, and DCs to yield a more proinflammatory state, initiating antitumor activity.

**Fig. 7.**
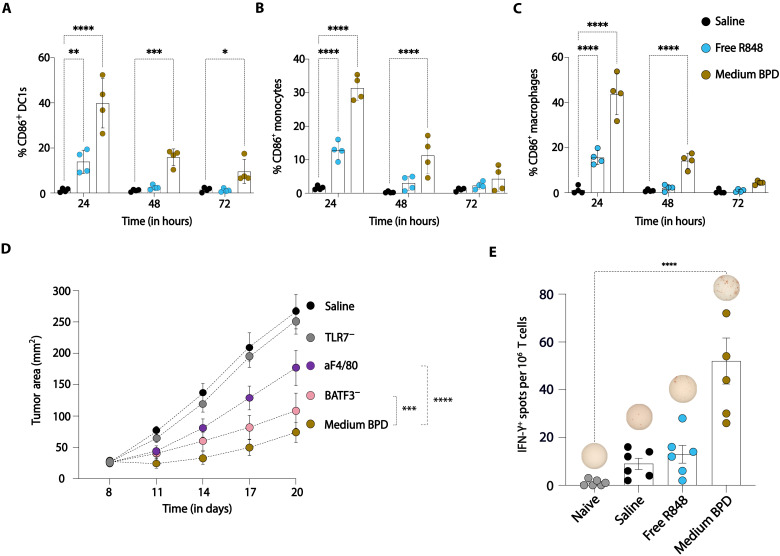
Myeloid cell activation is a key requirement for T cell priming and antitumor efficacy of R848-BPDs. (**A** to **C**) Immunophenotyping of tumor-draining lymph nodes 24, 48, and 72 hours after R848-BPD dosing of C57BL/6 mice (*n* = 4 per group) bearing syngeneic MC38 tumors (~30 mm^2^). (**D**) Mice (*n* = 5 per group) bearing MC38 tumors (~25 mm^2^) were treated with medium BPD (7.5 mg/kg on days 8, 11, and 14), and tumor growth was followed longitudinally. Treatment experiments in wild-type mice treated with depleting antibodies against F4/80, BATF3 knockout mice, and TLR7^−/−^ mice are shown. (**E**) C57Bl/6 mice bearing MC38 tumors (*n* = 6 per group) were injected with R848-BPDs along with controls, and T cells were isolated on day 7 posttreatment and cocultured with irradiated MC38 cells for IFN-γ enzyme-linked immunosorbent spot (ELISpot) analysis. Statistical comparisons were tested using two-way ANOVA with Dunnett’s multiple-comparisons test. *P < 0.05, **P < 0.01, ***P < 0.001, and ****P < 0.0001.

To assess whether the changes induced in the TME extended to the tumor-draining lymph nodes, we carried out immunophenotyping studies at 24, 48, and 72 hours after R848-BPD dosing. CD86 expression levels measured for up to 72 hours pointed to sustained activation of cDC1s, monocytes, and macrophages when compared with free R848 and saline-treated controls (Fig. 7, A to C, and fig. S17). To gain further insight into the pathways and cell types most critical for the effectiveness of R848-BPD therapy, we analyzed therapeutic responses in mice lacking TLR7 or deficient in key DC/myeloid cell populations. As expected, there was a complete abrogation of antitumor efficacy in TLR7 knockout mice, indicating that TLR7 activation is necessary for the efficacy of the R848-BPDs although IMDs have been shown to activate other innate immune pathways (Fig. 7D) (*46*). We tested the relative importance of macrophages to the therapeutic response by treating animals in the presence of depleting antibodies against F4/80 (fig. S18). As shown in Fig. 7D, therapeutic efficacy was substantially reduced under this depletion condition (*P* < 0.0001).

Last, an important potential mechanism of action for R848-BPD therapy would be activation of cross-presenting DC populations, which capture tumor antigens and migrate to tumor-draining lymph nodes to activate tumor-specific CD8^+^ T cells. Enzyme-linked immunosorbent spot (ELISpot) analysis of IFN-γ production was performed on T cells isolated from the spleen of mice 7 days after treatment with one dose of either saline, free R848, or R848-BPDs. Upon culturing with irradiated MC38 cells, we observed that even a single dose of R848-BPDs was able to amplify the endogenous T cell response against MC38 tumors (Fig. 7E). Consistent with this finding, treatment of mice deficient in Batf3-dependent cross-presenting DCs also led to a significant reduction in antitumor efficacy (Fig. 7D). Thus, we have demonstrated that our R848-BPD approach can efficiently activate myeloid cells to promote T cell responses against tumors to boost efficacy while minimizing irAEs commonly associated with systemic administration of TLR7/8 agonists.

## DISCUSSION

Following imiquimod (R837) receiving FDA approval as a topical treatment for actinic keratosis and basal cell carcinoma, there was a substantial push toward advancing more potent IMDs such as resiquimod (R848) into clinical studies using systemic administration in the context of melanoma and other solid malignancies (*47*–*50*). A notable observation in these clinical trials was the significant levels of inflammation that correlated with objective responses in primary and metastatic lesions in multiple tumor types; however, these antitumor responses occurred concomitant with severe grade 3 and 4 irAEs, which halted the clinical progress of these compounds (*51*–*53*). This outcome was attributed to unacceptably high inflammatory cytokine levels (such as TNF-α) in the blood, with multiple studies commenting on the narrow therapeutic index available for systemic delivery (*21*, *54*).

We hypothesized that a prodrug system that preferentially releases IMDs after they have accumulated in the tumor would be able to address this issue. Testing this hypothesis requires the synthesis of a series of prodrugs that only differ by their release kinetics while having otherwise identical physical properties, which is traditionally very difficult. The unique structure of our BPDs overcomes this challenge, allowing us to synthesize a small library R848-BPDs and test their biological properties with the goal of maximizing tumor accumulation while minimizing free IMD exposure in off-target organs to enable safe, systemic delivery of IMDs. We found that BPDs with sufficiently slow R848 release kinetics in vitro (MM *T*_1/2_ > ~12 days) showed lower early acute peak levels of R848 in the peripheral blood following dosing, thereby blunting early systemic cytokine levels compared to free R848 dosing. At later times, BPDs show sustained low levels of the drug in circulation, which do not trigger systemic inflammation in the blood but may be important for the enhanced efficacy of these IMD prodrugs. This finding potentially indicates a threshold concentration of R848 in serum below which there is minimal systemic inflammation and allowed us to then focus on testing these BPDs for their antitumor potential. We have already established that BPDs preferentially accumulate in tumor tissues with the liver as the main route of clearance (*55*). We leveraged this predictable biodistribution profile to our advantage: It is known that Kupffer cells in the liver have low proinflammatory cytokine production upon TLR7 engagement, a blunted response thought to be related to their role in maintaining tolerance (*56*, *57*). As a result, our IMD-BPDs accumulating in liver tissue did not induce immune toxicities as evidenced by minimal changes in alanine aminotransferase (ALT) levels upon systemic administration; thus, we were able to focus on optimizing tumor versus blood drug release through linker design. By adding functional groups to our phenyl ester–based linker structures that connect R848 covalently to BPDs, we were able to find R848-BPDs that avoid systemic toxicities generated by premature exposure of drug in the blood. This system can be further enhanced by other tumor-specific linker strategies now that we have demonstrated a proof-of-concept approach to minimize systemic toxicities.

Nonlinear dose-toxicity relationships confound MTD studies for IMDs such as R848 and are thus difficult to precisely define (*58*). We determined the MTD of free R848 in healthy C57BL/6J mice by monitoring for clinical signs and weight loss over a period of 1 week. Our preliminary decision was based on less than 5% of total body weight loss and no change in the body condition score of the mice. The R848-BPD library was also evaluated according to the above two parameters, and we observed linker-dependent increases in MTD values. Beyond weight loss and body condition score, cytokine levels in the blood were also assessed as a second layer of toxicity monitoring. Last, whole blood analysis and toxicity panels were evaluated to obtain a final round of biomarkers to correlate systemic side effects with serum concentrations of R848. We were able to successfully tune the levels of these markers through linker design allowing for controlled immune activation. Furthermore, fluorescently labeled R848-BPDs demonstrated increased tumor accumulation over time, significant uptake in myeloid populations in the tumor and draining lymph nodes, and activation of inflammatory cytokines in the tumor microenvironment. By directing uptake of BPDs into antigen-presenting cells (APCs) rather than other cells within the tumor environment, we were able to enhance activation of APCs and downstream T cell priming while also improving their safety profiles. This effect resulted in significantly stronger tumor growth inhibition and improved survival rates compared to mice given free R848 alone. Reprogramming subsets of macrophages and cDC1s played a crucial role in tumor control as depleting these cells abrogated antitumor efficacy demonstrated by R848-BPDs.

We note that this work comprises the first example of using bottlebrush polymer prodrugs as immune stimulants. The concept of using molecular design to tune release and to understand in detail the impact of release kinetics on immune response has not been explored and is uniquely enabled by our BPD platform. Furthermore, the bottlebrush conformation has been shown to improve tissue penetration and cell uptake (*59*). These are very small prodrug constructs that are in a size range not accessible to many other systems. Last, we note that the bottlebrush configuration shields the payload from burst release in serum. We hypothesize that future work toward optimizing the dimensions of our R848-BPDs and attaching targeting moieties (such as antibodies against proteins expressed on tumors) can further increase their uptake in the tumor microenvironment (*60*, *61*). This approach is of particular relevance given that TLR7 antibody drug conjugates (TLR7-ADCs) targeting human epidermal growth factor receptor 2 have shown promising antitumor inflammatory responses in mouse models and early clinical trials (*62*, *63*); however, the benefits of tumor antigen targeting and Fcγ receptor–mediated phagocytosis come with significant issues such as highly limited drug loading, antidrug antibodies and other immunotoxicities that were associated with decreases in drug exposure in clinical studies (*64*).

Last, we showed that maximal therapeutic activity from R848-BPD therapy requires repeat dosing to drive the antitumor immune response. To achieve optimal efficacy, we hypothesize that defining optimal dosing and timing intervals will be critical. One key factor for defining an optimal dosing strategy is TLR tolerance: TLR tolerance is defined as a transient state of refractoriness of TLRs to subsequent activation after initial dosing. This hyporesponsiveness has evolved to avoid the induction of autoimmunity through repeated TLR agonism; however, it has important implications for dosing schedules involving IMDs such as R848 that aim to generate antitumor immune responses. Although TLR7 tolerance has been observed in multiple studies, the mechanism of induction and avoidance remains undetermined (*65*, *66*). Using insights gained from tuning release rates via drug-linker composition, our future work involves understanding the appropriate dosing scheme to circumvent TLR7 tolerance and testing our optimized therapeutic strategy in more rigorous genetically engineered mouse models.

In summary, we have developed a BPD platform with distinct advantages for delivery of immune stimulants with narrow therapeutic indexes such as IMDs, which allows for their systemic administration through improving their safety and enabling conversion of cold tumors into hot tumors via sustained myeloid cell activation. Our results establish the ability to (i) generate R848 prodrugs and R848-BPDs of precise sizes, (ii) control release of free R848 in vitro and in vivo, and (iii) demonstrate antitumor effects of R848-BPDs in multiple syngeneic mouse models of colon carcinoma. We therefore conclude that finding an optimal tumor versus blood drug release window is a promising approach for safe, systemic delivery of small-molecule immunomodulators to potentiate antitumor responses to existing cancer immunotherapies.

## MATERIALS AND METHODS

### General procedure for synthesis of R848-MMs

One of six R848-N_3_ compounds: R848-PE-N_3_ (5a), R848-TEG-dMPE-N_3_ (5f), R848-OMPE-N_3_ (5b), R848-MPE-N_3_ (5c), R848-dOMPE-N_3_ (5d), R848-dMPE-N_3_ (5e), or Cy5.5-N_3_ and PEG-Alkyne-MM (*67*) were added to a 20-ml scintillation vial containing a stir bar. In a nitrogen-filled glove box, the reagents were dissolved in tetrahydrofuran (THF; 1 ml of THF per 100 mg of PEG-alkyne-MM) followed by addition of ~3 equivalents of copper (i) acetate. The reaction was stirred for 1 hour until consumption of PEG-alkyne-MM was observed by LC-MS analysis. The crude product was purified by preparative gel permeation chromatography. Fractions containing the product were concentrated by rotary evaporation, and the resulting residue was redissolved in THF and dried over sodium sulfate. Last, the solid polymer was washed with cold diethyl ether three times to afford the desired R848-MM or Cy5.5-MM as white and green solids, respectively.

### General procedure for synthesis of R848-BPDs

R848-dMPE-MM (2.00 g, 9.9 equivalents) and Cy5.5-MM (20.1 mg, 0.1 equivalents) were added to a 40-ml vial containing a Teflon-coated stir bar. In a nitrogen-filled glove box, the G3 solution was prepared: G3 (49.8 mg in 2.49 ml of THF = 0.02 g/ml^−1^). An aliquot of G3 (1.75 ml, 1 equivalent) was added to a stirred solution of MM (3.07 ml; THF) as a single stream. The final volume in the reaction vial (3.07 ml + 1.75 ml = 4.82 ml) afforded an MM concentration of 0.1 M. The reaction was allowed to proceed for 25 min before removal from the glove box and quenching with ethyl vinyl ether (0.300 ml). The material was diluted with nanopure H_2_O (~9.5 ml; 1:1 dilution) and then transferred to dialysis tubing (RC, 8-kDa molecular weight cutoff) for dialysis against nanopure H_2_O (10 liter, 3 × 2 to 3 hours per cycle). The contents of the dialysis tubing were then transferred to clean 20-ml vials and lyophilized (48 hours) to afford a dry powder (2.1 g, 90%).

### In vitro R848 release assay in PBS

Approximately 2 mg of a given MM (PE, tdMPE, OMPE, MPE, dOMPE, or dMPE) were weighed into a clean 4-ml vial. Four milliliters of PBS (pH = 7.4) containing 4-bromobenzyl alcohol (100 μM; internal standard) was added into each vial. All vials were sealed and placed in an incubation oven set at 37°C. At each time point, a vial for a given MM was removed from the incubation oven, and 50-μl aliquots were taken in LC-MS vials. A total of 200 μl of dimethylsulfoxide was added to each vial, and the resulting solution was briefly vortexed before filtering through a 0.45-μm nylon syringe filter. Analysis by LC-MS provided insight into the amount of R848 released at a given time point. Quantifications were made by integration of the released R848 peak and disappearing MM peak [LC conditions: 5 to 95% MeCN/H_2_O (0 to 7 min), 95 to 100% MeCN/H_2_O (7 to 8 min), and ZORBAX StableBond-C18 rapid resolution HT column].

### Cells

Human TLR7, mouse TLR7 HEK293, and RAW-Blue reporter cells were purchased from InvivoGen and were cultured following the vendor’s instructions. Murine BMDCs were harvested using a previous protocol (*68*). Briefly, bone marrow harvested cells from 8-week-old female C57BL/6J mice were cultured in RPMI 1640 containing 10% fetal bovine serum (FBS), penicillin and streptomycin (P/S; 100 U ml^−1^), 50 μM β-mercaptoethanol, Flt3L (600 ng/ml), and granulocyte-macrophage colony-stimulating factor (5 ng/ml). The medium was changed on day 5 and on day 9; nonadherent cells were harvested, counted, and plated for the assay. MC38 cells were provided by J. Schlom (National Cancer Institute) and cultured in Dulbecco’s modified Eagle’s medium (GE HealthCare Life Sciences) supplemented with 10% FBS and P/S (100 U ml^−1^). CT-26 cells were purchased from American Type Culture Collection and cultured in RPMI 1640 with 10% FBS, penicillin (100 U/ml), and streptomycin (100 μg/ml).

### In vitro innate immune stimulation

Human TLR7, mouse TLR7 HEK293, or RAW-Blue reporter cells (InvivoGen) were seeded at 3 × 10^4^ cells/96-well plate for 24 hours and then treated with R848, R848-BPDs, PBS, or dimethyl sulfoxide for 48 hours. To assess innate immune stimulation, secreted alkaline phosphatase was measured from supernatants using QuantiBlue reagent (InvivoGen) to confirm dose-dependent activation of the target TLR receptor. R848-BPDs were confirmed to have low endotoxin levels (<0.1 Endotoxin Units per dose) by the Endosafe Nexgen-PTS system (Charles River).

Alternatively, the immune stimulatory potential of R848 and R848-BPDs in BMDCs was assessed as well. BMDCs were seeded in a 96-well plate (Corning) at 200,000 cells per well. After 24 hours of culture, the medium was replaced with fresh medium or fresh medium with 75 μl of blank polymer (PEG), R848, R848-BPDs or PBS. After 48 hours of incubation, induction of DC maturation by the R848-BPDs was assessed by flow cytometry analysis of the expression of the costimulatory receptor CD86.

### Mice

All in vivo experiments were performed in the following mouse strains: 8-week-old female C57BL/6J mice (the Jackson Laboratory), 8-week-old female BALB/c mice (the Jackson Laboratory), 8-week-old BATF3^−^ mice (the Jackson Laboratory, strain no. 013755), and 8-week-old TLR7^−^ mice (the Jackson Laboratory, strain no. 008380). Experiments were performed in specific pathogen–free animal facilities at the MIT Koch Institute for Integrative Cancer Research. Mice were housed under standard 12-hour light–12-hour dark conditions with ad libitum access to water and chow. All mouse studies were performed according to institutional and National Institutes of Health guidelines for humane animal use and in accordance with the Association for Assessment and Accreditation of Laboratory Animal Care. Protocols were approved by the Institutional Animal Care and Use Committee at MIT.

### Dose escalation and toxicity analysis to identify MTD in vivo

The MTD of free R848 was determined in healthy C57BL/6J mice by intravenous injection at five different doses (3, 5, 7.5, 10, and 15 mg/kg) followed by monitoring for clinical signs and weight loss over a period of 1 week. Body condition score parameters such as visible signs of restricted movement and lethargy were also noted for the mice.

Next, we carried out dose escalation studies to identify the MTD for each BPD, starting from a dose of 7.5 mg/kg. MTD values for R848-BPDs were quantified in accordance with MIT Committee on Animal Care policies. Healthy C57LB/6 mice were administered each R848-BPD sample at five different R848 doses (7.5, 15, 30, 50, and 75 mg/kg) by intravenous injection (*n* = 3 animals per group). The MTD was determined by monitoring for weight loss and clinical signs for up to 2 weeks.

In parallel, separate groups of mice were set up where serum samples were collected by cheek bleeds 4, 24, and 48 hours after one injection (at MTD) of R848 and R848-BPDs and frozen at 20°C until analysis. Samples were diluted 1:1 with assay buffer and assayed using the LEGENDplex mouse antivirus response panel (BioLegend) according to the manufacturer’s instructions. The cytometric bead array readout was performed using a BD FACS LSRFortessa cytometer and analyzed using LEGENDplex v8.0 software.

Last, a third arm of the acute dosing analysis included blood chemistry analysis (complete blood count), and serum samples were also sent to IDEXX Reference Laboratories for analysis of liver enzyme levels (ALT) and blood urea nitrogen. For subsequent tumor imaging and therapy studies, the dose of R848 and R848-BPDs for safe administration was set as the MTD for R848 (i.e., the MTD that results in <5% weight loss in >80% of animals). These studies outlined a tolerable dosing regimen for tumor therapy.

### PK analysis in vivo

Free R848, R848-MPE-BPDs, and R848-dMPE-BPDs were administered intravenously (at MTD dose) to C57LB/6 mice (*n* = 5 animals per group). Blood PK was assessed by drawing blood for LC–tandem MS analysis to quantify free R848 levels at 1, 3, 6, and 24 hours.

Plasma samples were extracted by mixing 5-μl plasma with 45-μl extraction mix (acetonitrile/methanol 75:25 with 0.1% formic acid) containing 50 nM imiquimod as an internal standard. Samples were vortexed at 4°C for 10 min and cleared by centrifugation at 4°C at 17,000*g* for 10 min. Supernatants were transferred to LC-MS vials for analysis. Working stocks of resiquimod were made by diluting resiquimod in extraction mix containing 50 nM imiquimod as internal standard. A matrix-matched calibration curve was made by mixing 5-μl working stock resiquimod with 45-μl extracted control plasma. Analysis was conducted on a Q Exactive bench top Orbitrap mass spectrometer equipped with an Ion Max source and a HESI II probe, which coupled to a Dionex Ulitmate 3000 HPLC system (both Thermo Fisher Scientific, San Jose, CA). Five microliters of sample was injected onto a Kinetex C18 50 × 2.1 mm analytical column (2.6-μm particle size; Phenomenex). The column oven and autosampler tray were held at 30° and 4°C, respectively. The following conditions were used to achieve chromatographic separation: Buffer A was 0.1% formic acid; buffer B was 0.1% formic acid in acetonitrile. The flow rate was 0.4 ml/min. The chromatographic gradient was run as follows: 0 to 3.5 min, linear gradient 5 to 80% B; 3.5 to 3.6 min, linear gradient 80 to 98% B; 3.6 to 4.5 min, the gradient was held at 98% B; 4.5 to 4.6 min, linear gradient 98 to 5% B; 4.6 to 6 min, the gradient was held at 5% B. The mass spectrometer was operated in positive mode, and data acquisition was performed using targeted selected ion monitoring scans centering on 241.14477 and 314.18155 mass/charge ratio to detect imiquimod and resiquimod, respectively. The resolution was set at 70,000, the AGC target was 1 × 10^5^, and the maximum injection time was 200 ms. Absolute quantification of resiquimod was performed using XCalibur QuanBrowser 2.2 (Thermo Fisher Scientific), using a 5–parts per million mass tolerance and referencing a matrix-matched calibration curve containing concentrations of resiquimod ranging from 1 nM to 100 μM and 50 nM imiquimod as internal standard. Curve fitting was achieved using a quadratic log-log fit, with an *R* value of 0.9992.

### MC38 syngeneic mouse model of colon adenocarcinoma

MC38 is derived from a colon tumor in a C57BL/6 mouse following long-term exposure to 1,2-dimethylhydrazine dihydrochloride. MC38 has a favorable response profile to innate immune agonists, suggesting a tumor microenvironment amenable to myeloid activation (*69*). Briefly, MC38 colon carcinoma cells were suspended at 0.5 × 10^6^ cells in 100 μl of PBS and inoculated subcutaneously to induce tumors in C57Bl/6 mice.

### Organ and cellular BD analysis in vivo

For tumor BD studies, 0.5 × 10^6^ MC38 cancer cells were inoculated subcutaneously in the hind flank of C57BL/6 mice (*n* = 5 animals per group). When tumors reached ~25 mm^2^ in size, the animals received an intravenous injection of R848-Cy5.5-BPDs. Tumors and other organs were collected at 0.5, 6, and 24 hours, homogenized, and the total fluorescence signal was measured on a fluorescence plate reader to quantify the R848-Cy5.5-BPD in each organ. Briefly, the extracted tissues were weighed and then mechanically dissociated until homogenous in lysis buffer [100 mM Hepes (pH 7), 2 weight % of Triton X-100, and 5 mM EDTA] using disposable tissue grinder tubes (Kimble Biomasher). Subsequently, the tubes were vortexed for 30 s and centrifuged (300*g*, 2 min), and the supernatants were transferred to a black 96-well plate for quantification using a fluorescence plate reader (excitation of 675 nm, emission of 720 nm). In parallel, flow cytometry was used to quantify the cellular uptake of R848-Cy5.5-BPDs in homogenized tumor, tumor-draining lymph node, and spleen samples harvested 24 hours after intravenous injection of R848-Cy5.5-BPDs.

### Intravital imaging

Dorsal imaging window was implanted into the dorsal skin of mice (*n* = 2 animals per group) with CD11c^+^ cells expressing the fluorescent protein, Venus. The imaging window permitted stable imaging via confocal fluorescence microscopy on a Leica SP8 microscope over several days consecutively. The mice were inoculated with MC38 cancer cells expressing the fluorescent protein Cerulean. This cell line contained a transgene in which the yellow fluorescent protein is expressed under the transcriptional control of the integrin alpha X (Cd11c) promoter, which is expressed primarily by DCs. After 5 days of tumor outgrowth, large areas of the tumor were imaged using a tile scan to ascertain the number of CD11c-Venus^+^ cells in the tumor microenvironment. Mice were injected intravenously with R848-Cy5.5-BPDs; after 24 hours, the same area was imaged again.

### CT-26 syngeneic mouse model of colon carcinoma

CT-26 is an *N*-nitroso-*N*-methylurethane–induced undifferentiated colon carcinoma cell line established from BALB/c mice with aggressive colon carcinoma. The CT-26 colon carcinoma tumor model has high levels of immunosuppressive macrophages and myeloid-derived suppressor cells, providing a particularly relevant model for the development of new myeloid-based immunotherapies (*69*). Briefly, CT-26 cells were suspended at 1 × 10^6^ cells in 100 μl of PBS and inoculated subcutaneously to induce tumors in BALB/c mice.

### In vivo therapeutic efficacy

Mice were injected with 0.5 × 10^6^ or 1 × 10^6^ cells (MC38 or CT-26) subcutaneously on day 0. When tumors reached 25 mm^2^, animals were randomized into five groups and saline, blank polymer, free R848, R848-MPE-BPDs, or R848-dMPE-BPDs were administered via retro-orbital injection either as a monotherapy or in combination with anti–PD-1 (200 μg, intraperitoneally) on days 8, 11, and 14. Tumor area measurements were taken using calipers, and animal weights were assessed every 3 days starting on day 5 after tumor inoculation.

### Sample processing for scRNA-seq

Mouse MC38 tumor samples (*n* = 3 for saline group and free R848 group, and *n* = 2 for medium BPD group) were digested using the Tumor Dissociation Kit (Miltenyi Biotec) 24 hours after administration. Cells were then stained with CD45 microbeads, and CD45^+^ and CD45^−^ cells were separated using a MACS separator. CD45^+^ and CD45^−^ single cells for each mouse were loaded in a ratio of 9:1 onto Seq-Well arrays. scRNA-seq sample processing and library preparation followed the Seq-Well protocol (*70*, *71*). cDNA was loaded onto Illumina NextSeq (75-cycle NextSeq 500/550 v2.5 kit).

### scRNA-seq analysis

#### Quality control and clustering

scRNA-seq reads from each sequencing run were demultiplexed and aligned to the mm10 reference genome, as previously described (*72*). Raw feature barcode matrices for each sample were used for further analysis. Initial quality control was conducted using Seurat V4.0.0 with the following criteria: Cells with 400 < genes < 10,000 and Unique Molecular Identifier (UMI) < 50,000 were retained and cells with >15% mitochondrial reads were excluded. Genes were filtered by retaining those expressed in at least 10 cells. Initial clustering with the first 30 principal components (PCs) identified a cluster of poor-quality cells with high mitochondrial read fractions, which was subsequently excluded from downstream analyses. After identifying the top gene markers on each remaining cluster, we identified a cluster containing MC38 tumor cells based on expression of *Mtap*, *Rhox5*, *Col6a2*, and *Fam159b*. We subsequently focused further analyses on the nonmalignant clusters. After subsetting to only nonmalignant cells, we rescaled the dataset and identified 869 highly variable genes (0.2 < mean expression < 6 and 0.5 < dispersion < 20). We then reran PC analysis and used the first 11 PCs to cluster with a resolution of 1.3. By running differential gene expression analyses for each cluster relative to the rest with Wilcoxon rank sum test, we were able to identify marker genes to assign 16 fine-cell subsets; on the basis of expression of known markers, we combine clusters containing shared lineage information. For example, monocytes highly express *Ly6c2*, neutrophils highly express *S100a8/9*, and T cells highly express *Cd3d/g*.

#### Proportion analysis

To account for the dependencies between cell subset proportions in scRNA-seq data, we used both Fisher exact tests and a Dirichlet multinomial regression analysis as complementary approaches to look for shifts in cell frequencies across conditions. We performed Fisher exact tests using the number of cells from each cell subset between two conditions to test whether a cell type was enriched in one condition (with Benjamini-Hochberg multiple testing correction). To account for the proportions of all other cell types in comparison, we also used Dirichlet multinomial regression model from the Dirichlet_Reg_ R package and calculated the *P* values associated with abundance shifts (with Benjamini-Hochberg multiple testing correction) (*41*). We note that adjusted *P* values are shown for comparisons that are at least significant by one test.

#### Gene set enrichment analysis

To identify differentially expressed genes in each cell subset of interest, we used Wilcoxon rank sum test to evaluate the significance of shifts in the frequently expressed genes (minimum expression in 25% of cells in either population), filtering for a minimum log fold change in expression of 0.25 and Benjamini-Hochberg corrected *P* value of <0.05. GSEA was performed for genes with *P*_adj_ < 0.05 (Benjamini-Hochberg multiple testing correction) with the Enricher and GSEA functions in the clusterProfiler R package from Bioconductor (*73*).

#### TF enrichment analysis

To identify the TFs enriched in each cell subset of interest, we used Virtual Inference of Protein-activity by Enriched Regulon analysis (VIPER) in combination with DoRothEA to estimate the TF activities from gene expression data (*74*). We used the “mm_pancancer” reference for TF-target interactions that contain 1096 TFs targeting 17,695 genes (*43*). We only selected the TF-target interactions with high confidence (category A and B defined by DoRothEA) and defined *P*_adj_ < 0.01 (Benjamini-Hochberg multiple testing correction) as significantly enriched TF between groups.

### Immunophenotyping studies

For immunophenotyping studies, 0.5 × 10^6^ MC38 cancer cells were inoculated subcutaneously in the hind flank of C57BL/6 mice (*n* = 4 animals per group). When tumors reached ~30 mm^2^ in size, the animals received an intravenous injection of R848-Cy5.5-BPDs. Tumor-draining lymph nodes were collected at 24, 48, and 72 hours after dosing; mechanically dissociated; and analyzed by flow cytometry. Antibodies to CD103 (2E7), Ly6C (HK1.4), F4/80 (BM8), CD11b (M1/70), CD86 (GL1), MHC2 or I-A/I-E (M5/114.15.2), CD24 (30-F1), CD11c (N418), CCR7 (4B12), CD169 (3D6.112), and Ly6G (1A8) were obtained from BioLegend. Antibodies to CD45 (30-F11) and CD8α (53-6.7) were purchased from BD Biosciences. All antibodies were diluted 1:100. Viability was assessed using Zombie Aqua (1:1000; BioLegend). Flow cytometry sample acquisition was performed on an LSRFortessa cytometer (BD Biosciences), and the collected data were analyzed using FlowJo v10.5.3 software (TreeStar).

### Cellular depletion studies

To determine the relative importance of innate immune cell subsets in the efficacy of R848-BPDs, C57Bl/6 mice (*n* = 5 animals per group) were inoculated with MC38 tumor cells as above and treated with R848-BPDs in the presence of depleting antibodies against F4/80 (200 μg, intraperitoneally) every day (beginning 1 day before therapy) to ascertain the relative contribution of macrophages to therapeutic response. The role of cross-presenting DCs was assessed using *Batf3*^−/−^ mice, which lack this DC population, while TLR7^−^ mice were used to validate the dependency on TLR7 activation.

### Induction of activated T cell responses via ELISpot

To assess induction of tumor-specific T cell responses against treated tumors, we used the IFN-γ ELISpot assay of T cells cocultured with irradiated MC38 tumor cells. Effector cells were CD3^+^ T cells isolated from MC38-tumor bearing mice (*n* = 6 animals per group) 7 days posttreatment with saline, R848, or R848-BPDs. Spleens were isolated from mice, mechanically digested through 70-μm nylon cell strainers to prepare single-cell suspensions for CD3^+^ T cell isolation by the CD3^+^ T cell isolation kit (STEMCELL Technology). Isolated CD3^+^ T cells were suspended in RPMI supplemented with 10% FBS, 1% P/S, 1× nonessential amino acids (Invitrogen), 1× sodium pyruvate (Invitrogen), and 1× 2-mercaptoethanol (Invitrogen). Target MC38 cells were treated with mouse IFN-γ (50 U/ml; PeproTech) for 12 hours and then irradiated (120 Gy) on the day of the experiment followed by trypsinization into a single-cell suspension, washed two times in 1× PBS to remove residual IFN-γ, and suspended in the same media as the effectors. Targets were seeded at 25,000 cells per well while effectors were seeded at 250,000 cells per well. Plates were wrapped in foil and incubated for 24 hours and then developed according to the manufacturer’s protocol. Plates were scanned using a CTL ImmunoSpot plate reader, and the data were analyzed using CTL ImmunoSpot software.

### Statistical analysis

Statistical analysis and graphing were done with GraphPad Prism. Two-tailed Student’s *t* test was used to compare two experimental groups, and two-way analysis of variance (ANOVA) with Dunnett’s post hoc analysis was used for comparing more than two groups. Details of the statistical test and number of replicates are indicated in the figure legends with *P < 0.05, **P < 0.01, ***P < 0.001, and ****P < 0.0001. A value of *P* < 0.05 was considered statistically significant.
