# Supplementary material for: Engineering kinetics of TLR7/8 agonist release from bottlebrush prodrugs enables tumor-focused immune stimulation
Source: Sci Adv. 2023 Apr 19;9(16):eadg2239. doi: 10.1126/sciadv.adg2239 (PMC10115420; doi:10.1126/sciadv.adg2239)
Supplement: Supplementary file 1 — Supplementary Materials and Methods Figs. S1 to 18 Tables S1 to S3 Legend for table S4 [file sciadv.adg2239_sm.pdf]

Science Advances
AAAS

Supplementary Materials for

Engineering kinetics of TLR7/8 agonist release from bottlebrush prodrugs enables tumor-focused immune stimulation

Sachin H. Bhagchandani *et al.*

Corresponding author: Darrell J. Irvine, djirvine@mit.edu; Jeremiah A. Johnson, jaj2109@mit.edu

Sci. Adv. **9**, eadg2239 (2023)
DOI: 10.1126/sciadv.adg2239

The PDF file includes:

Supplementary Materials and Methods
Figs. S1 to 18
Tables S1 to S3
Legend for table S4

Other Supplementary Material for this manuscript includes the following:

Table S4

Table of Contents

Materials and Methods 4

Small Molecule Syntheses 5

Esterification 5

Reduction 7

Azidation 9

Carbonyl 11

Resiquimod 13

NMR Spectra 16

Reduction 22

Azide 28

Carbonyl 34

Resiquimod 40

Macromonomer 46

Cryo-transmission electron microscopy (Cryo-TEM) images and measurements 48

Release rate measurements in-vitro 50

In-vitro assays 51

In-vivo maximum tolerable dose data for R848-BPDs 52

In-vivo multi-dose weight loss data for R848-BPDs 54

Single-cell RNA-sequencing (scRNA-seq) 55

Gating strategy for immunophenotyping 57

Cell depletion studies 58

A

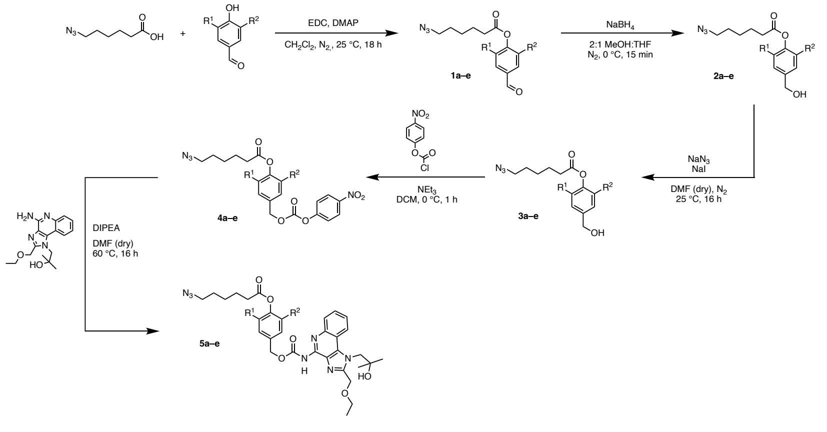

B

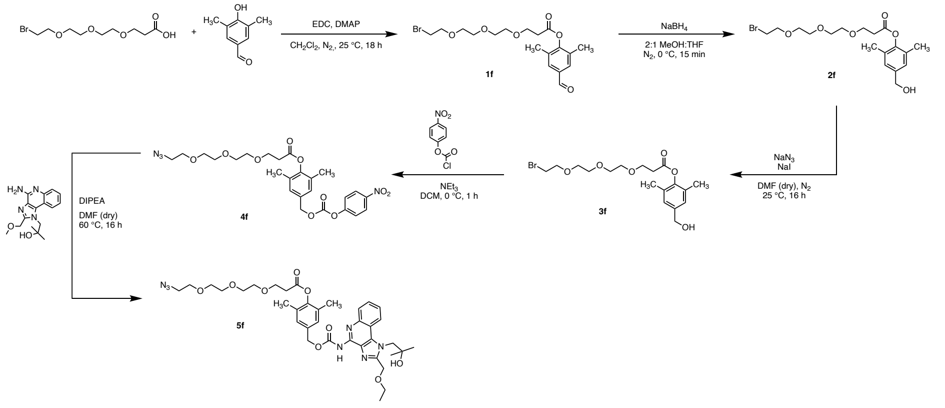

C

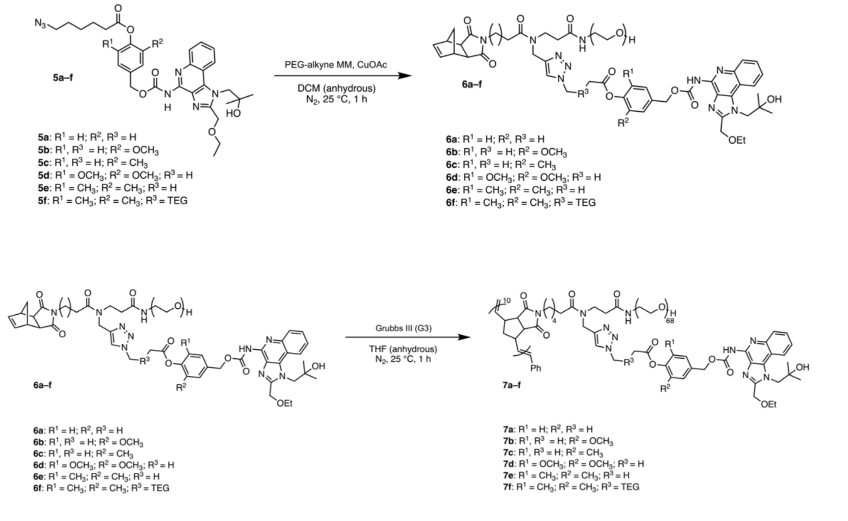

Fig. S1. Synthetic steps to **(A)** resiquimod (R848) containing small molecules 5a-e (R848-PE-N3, R848-OMPE-N_3, R848-MPE-N_3, R848-dOMPE-N_3, & R848-dMPE-N_3) **(B)** resiquimod (R848) containing small molecule 5f (R848-TEG-dMPE-N_3) **(C)** R848-MMs & R848-BPDs

Materials and Methods

Materials

Solvents used are of HPLC grade, purchased from Millipore Sigma, and used as received unless otherwise noted. Deuterated solvents were purchased from Cambridge Isotope Laboratories, Inc.

Nuclear magnetic resonance spectroscopy (NMR)

^{1}H and ^{13}C{^{1}H} NMR spectra were recorded using either a 500 MHz (three-channel) or 600 MHz (four-channel) Bruker AVANCE NEO NMR spectrometer at 25 °C. Chemical shifts are referenced to the relevant residual protonated solvent peak, and are reported as parts per million (ppm) with splitting patters designated as follows: s (singlet), d (doublet), t (triplet), q (quadruplet), p (pentet), m (multiplet), and br (broad).

Mass spectrometry (MS)

Low resolution liquid chromatography mass spectrometry (LC-MS) was performed using an Agilent 6125B mass spectrometer attached to an Agilent 1260 Infinity LC. It utilizes an electrospray (ESI) source. ChemStation acquisition and data analysis software is used. High resolution mass spectrometry (HR-MS) measurements were recorded using a JEOL AccuTOF 4G LC-plus system equipped with an ionSense DART (Direct Analysis in Real Time) source. The system operates with an accuracy of 5 ppm and a resolving power >10,000 (FWHM). msAxel was used as the acquisition and data processing software.

Size Exclusion Chromatography (SEC)

SEC characterization was done using an Agilent 1260 LC system equipped with a Wyatt T-rEX refractive index detector and Wyatt DAWN HELEOS 18 angle light scattering detector. R848-MMs and R848-BPDs were run on Agilent PL1110-6500 columns in tandem at a temperature of 60 °C and flow rate of 1 mL/min with dimethyl formamide (DMF) containing 0.025 M LiBr as the eluent.

Cryo Transmission Electron Microscopy (cryo-TEM)

R848-BPD imaging was performed using a JEOL 2100 FEG TEM. R848-BPDs were prepared at 2 mg/mL in water, and sample preparations were performed using a 930 Gatan Cryo-Plunge 3. Data collection and analysis was done using Gatan Microscopy Suite Digital Micrograph (Version 2.32.888.0)

Dynamic Light Scattering (DLS)

DLS was performed using a Wyatt Dyna Pro Plate Reader. BPD suspensions were prepared in a solution of nanopure water (MilliQ) (1mg/ml). Measurements were made in sets of 10 acquisitions. The average hydrodynamic diameters were calculated by using the DLS correlation function via a regularization fitting method (Dynamics 7.4.0.72 software package from Wyatt Technology).

Preparative gel-permeation chromatography (prep-GPC)

prep-GPC was performed on a JAI Preparative Recycling HPLC (LaboACE-LC-5060) system equipped with 2.5HR and 2HR columns in series (20 mm ID x 600 mm length) using $CHCl_3$ as the eluent.

Small Molecule Syntheses

Esterification

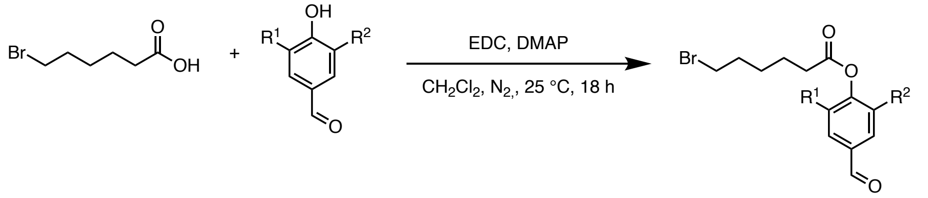

1a: R^1 = H; R^2 = H
1b: R^1 = H; R^2 = OCH_3
1c: R^1 = H; R^2 = CH_3
1d: R^1 = OCH_3; R^2 = OCH_3
1e: R^1 = CH_3; R^2 = CH_3

or

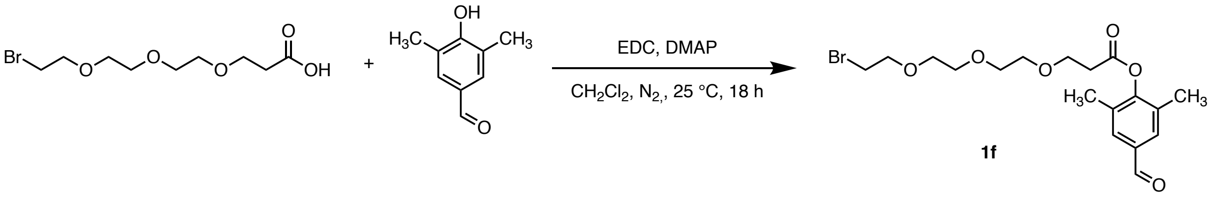

1f

General procedure. Compounds prepared using an adapted procedure from Vohidov et al.[32] To an oven-dried Schlenk flask was added 6-bromohexanoic acid (1.05 equiv) *or* 3-(2-(2-(2-bromoethoxy)ethoxy)ethoxy)propanoic acid (bromo-PEG3-acid, 1.05 equiv), EDC•HCl (1.10 equiv), and DMAP (0.10 equiv), and the solids placed under an N_2 atmosphere. Dry DCM (10 mL) was then added, and reaction left to stir at room temperature for 5 min. The appropriate benzaldehyde (1.0 equiv) was added against the flow of N_2, following which the reaction mixture was stirred at room temperature for 18 h. H_2O (10 mL) was added to quench the reaction, and the aqueous layer was washed with DCM (3 x 10 mL). The combined organics were dried using Mg_2SO_4, filtered, and the solvent removed under reduced pressure. The crude product was purified using column chromatograph (silica, 9:1 DCM:EtOAc).

1a (R^1 = H; R^2 = H): (colorless oil, 0.986 g, 3.30 mmol, 68% yield). ^{1}H NMR (500 MHz, $CDCl_3$) δ 10.00 (s, 1H), 8.06 – 7.82 (m, 2H), 7.35 – 7.17 (m, 2H), 3.45 (t, *J* = 6.7 Hz, 2H), 2.63 (t, *J* = 7.4 Hz, 2H), 1.94 (dt, *J* = 14.3, 6.8 Hz, 2H), 1.81 (p, *J* = 7.5 Hz, 2H), 1.66 – 1.54 (m, 2H). $^{13}C\{^1H\}$ NMR (126 MHz, $CDCl_3$) δ 191.0, 171.3, 155.5, 134.1, 131.3, 122.4, 34.2, 33.5, 32.4, 27.6, 24.0. DART-MS (m/z) calculated for $C_{13}H_{16}O_3Br$: 299.02773; found 299.03010 $[M+H]^+$.

1b (R^1 = H; R^2 = OCH_3): (colorless oil, 1.09 g, 3.31 mmol, 68% yield). ^{1}H NMR (500 MHz, $CDCl_3$) δ 9.94 (s, 1H), 7.51 – 7.44 (m, 2H), 7.20 (d, *J* = 7.9 Hz, 1H), 3.89 (s, 3H), 3.43 (t, *J* = 6.7 Hz, 2H), 2.63 (t, *J* = 7.4 Hz, 2H), 1.93 (dt, *J* = 14.3, 6.9 Hz, 2H), 1.80 (p, *J* = 7.4 Hz, 2H), 1.64 – 1.53 (m, 2H). $^{13}C\{^1H\}$ NMR (126 MHz, $CDCl_3$) δ 191.1, 170.9, 152.1, 145.1, 135.3, 124.8, 123.5, 110.9, 56.2, 33.8, 33.6, 32.4, 27.6, 24.1. DART-MS (m/z) calculated for $C_{14}H_{18}O_4Br$: 329.03830; found 329.04063 $[M+H]^+$.

1c (R^1 = H; R^2 = CH_3): (colorless oil, 1.20 g, 3.83 mmol, 78% yield). ^{1}H NMR (500 MHz, $CDCl_3$) δ 9.96 (s, 1H), 7.78 (d, *J* = 2.0 Hz, 1H), 7.74 (dd, *J* = 8.2, 2.1 Hz, 1H), 7.19 (d, *J* = 8.2 Hz, 1H), 3.44 (t, *J* = 6.7

Hz, 2H), 2.64 (t, *J* = 7.5 Hz, 2H), 2.26 (s, 3H), 1.94 (dt, *J* = 14.9, 6.9 Hz, 2H), 1.81 (dt, *J* = 15.4, 7.5 Hz, 2H), 1.66 – 1.53 (m, 2H). ^{13}C NMR (126 MHz, $CDCl_3$) δ 191.2, 171.0, 154.1, 134.2, 132.1, 131.4, 128.9, 122.9, 34.0, 33.4, 32.3, 27.6, 24.1, 16.3. DART-MS (m/z) calculated for $C_{14}H_{18}O_3Br$: 313.04338; found 313.04557 $[M+H]^+$.

1d (R^1 = OCH_3; R^2 = OCH_3): (white powder, 0.710 g, 1.98 mmol, 81% yield). ^{1}H NMR (500 MHz, $CDCl_3$) δ 9.90 (s, 1H), 7.14 (s, 2H), 3.89 (s, 6H), 3.44 (t, *J* = 6.7 Hz, 2H), 2.65 (t, *J* = 7.3 Hz, 2H), 1.97 – 1.88 (m, 2H), 1.81 (p, *J* = 7.4 Hz, 2H), 1.65 – 1.56 (m, 2H). $^{13}C\{^1H\}$ NMR (126 MHz, $CDCl_3$) δ 191.2, 170.7, 153.0, 134.4, 134.0, 106.2, 56.5, 56.5, 33.7, 33.6, 32.5, 27.6, 24.3. DART-MS (m/z) calculated for $C_{15}H_{22}O_5Br$: 361.06451; found 361.06420 $[M+H]^+$. DART-MS (m/z) calculated for $C_{15}H_{20}O_5Br$: 359.04886; found 359.04870 $[M+H]^+$.

1e (R^1 = CH_3; R^2 = CH_3): (white powder, 1.26 g, 3.84 mmol, 79% yield). ^{1}H NMR (500 MHz, $CDCl_3$) δ 9.92 (s, 1H), 7.61 (s, 2H), 3.45 (t, *J* = 6.6 Hz, 2H), 2.67 (t, *J* = 7.5 Hz, 2H), 1.95 (dq, *J* = 9.5, 6.8 Hz, 2H), 1.83 (p, *J* = 7.5 Hz, 2H), 1.66 – 1.57 (m, 2H). $^{13}C\{^1H\}$ NMR (126 MHz, $CDCl_3$) δ 191.5, 170.6, 153.1, 134.1, 131.5, 130.2, 33.7, 33.4, 32.3, 27.7, 24.2, 16.5. DART-MS (m/z) calculated for $C_{15}H_{20}O_3Br$: 327.05903; found 327.06058 $[M+H]^+$.

1f: (yellow oil, 0.900 g, 2.16 mmol, 80% yield). ^{1}H NMR (500 MHz, $CDCl_3$) δ 9.93 (s, 1H), 7.60 (s, 2H), 3.91 (t, *J* = 6.2 Hz, 2H), 3.81 (t, *J* = 6.3 Hz, 2H), 3.72 – 3.63 (m, 8H), 3.47 (t, *J* = 6.3 Hz, 2H), 2.91 (t, *J* = 6.2 Hz, 2H), 2.23 (s, 6H). $^{13}C\{^1H\}$ NMR (126 MHz, $CDCl_3$) δ 191.7, 169.0, 153.2, 134.2, 131.8, 130.3, 71.4, 70.81, 70.80, 70.7, 35.2, 30.5, 16.6. DART-MS (m/z) calculated for $C_{18}H_{26}O_6Br$: 417.09073; found 417.09182 $[M+H]^+$.

Reduction

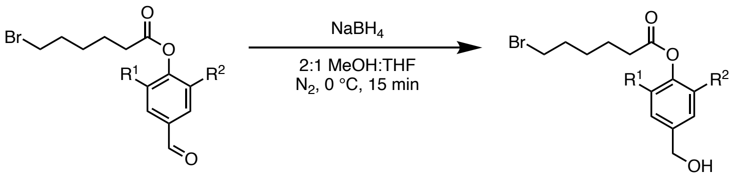

2a: R^1 = H; R^2 = H
2b: R^1 = H; R^2 = OCH_3
2c: R^1 = H; R^2 = CH_3
2d: R^1 = OCH_3; R^2 = OCH_3
2e: R^1 = CH_3; R^2 = CH_3

or

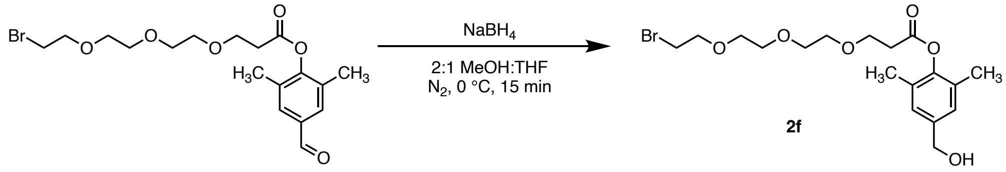

2f

General procedure. Compounds prepared using an adapted procedure from Vohidov et al.[1] To an oven-dried Schlenk flask was added the appropriate compound **1a–f** (1.0 equiv) and placed under an N_2 atmosphere. Dry THF (10 mL), and dry MeOH (10 mL) were added and the reaction vessel was immersed in an ice bath. Against the flow of N_2, $NaBH_4$ (1.5 equiv) was added in three portions as a solid, and the reaction left to stir at 0 °C for 15 min. Following this, H_2O (50 mL) was added, and the reaction mixture was extracted with DCM (3 x 20 mL). The combined organics were washed with brine (1 x 50 mL), dried with Mg_2SO_4, filtered, and the solvent removed *in vacuo*. The crude compound was purified using column chromatography (silica, 100% DCM to 1:1 DCM:EtOAc gradient).

2a (R^1 = H; R^2 = H): (colorless oil, 0.345 g, 1.15 mmol, 39% yield). ^{1}H NMR (500 MHz, $CDCl_3$) δ 7.40 – 7.32 (m, 2H), 7.13 – 6.98 (m, 2H), 4.65 (s, 2H), 3.43 (t, *J* = 6.7 Hz, 2H), 2.58 (t, *J* = 7.4 Hz, 2H), 1.96 – 1.88 (m, 2H), 1.78 (p, *J* = 7.5 Hz, 2H), 1.62 – 1.52 (m ^{13}C{^{1}H} NMR (126 MHz, $CDCl_3$) δ 172.1, 150.1, 138.6, 128.2, 121.7, 64.8, 34.2, 33.6, 32.4, 27.7, 24.1. DART-MS (m/z) calculated for $C_{13}H_{16}O_2Br$: 283.03282; found 283.03445 $[M–OH]^+$.

2b (R^1 = H; R^2 = OCH_3): (colorless oil, 0.422 g, 1.27 mmol, 44% yield). ^{1}H NMR (500 MHz, $CDCl_3$) δ 7.04 – 6.97 (m, 2H), 6.91 (dd, *J* = 8.0, 1.8 Hz, 1H), 4.67 (s, 2H), 3.83 (s, 3H), 3.44 (t, *J* = 6.7 Hz, 2H), 2.60 (t, *J* = 7.4 Hz, 2H), 1.93 (p, *J* = 7.0 Hz, 2H), 1.81 (dq, *J* = 15.3, 8.0 Hz, 2H), 1.65 – 1.54 (m, 2H). ^{13}C NMR (126 MHz, $CDCl_3$) δ 171.7, 151.3, 140.0, 139.2, 122.8, 119.1, 111.1, 65.2, 56.0, 33.9, 33.7, 32.5, 27.7, 24.3. DART-MS (m/z) calculated for $C_{14}H_{20}O_4Br$: 331.05395; found 331.05488 $[M+H]^+$.

2c (R^1 = H; R^2 = CH_3): (colorless oil, 0.633 g, 2.10 mmol, 61% yield). ^{1}H NMR (500 MHz, $CDCl_3$) δ 7.24 (d, *J* = 2.0 Hz, 1H), 7.19 (dd, *J* = 8.2, 2.2 Hz, 1H), 6.98 (dd, *J* = 8.0, 2.5 Hz, 1H), 4.64 (d, *J* = 2.4 Hz, 2H), 3.44 (td, *J* = 6.8, 2.4 Hz, 2H), 2.60 (td, *J* = 7.6, 2.6 Hz, 2H), 2.17 (d, *J* = 2.4 Hz, 3H), 1.93 (p, *J* = 6.8 Hz, 2H), 1.85 – 1.77 (m, 2H), 1.59 (tdd, *J* = 10.3, 8.2, 4.0 Hz, 2H). ^{13}C{^{1}H} NMR (126 MHz, $CDCl_3$) δ 171.8,

148.9, 138.7, 130.4, 130.0, 125.7, 122.1, 65.0, 60.5, 34.1, 33.6, 32.5, 27.8, 24.3, 16.4. DART-MS (m/z) calculated for $C_{14}H_{18}O_2Br$: 297.04847; found 297.04994 $[M–OH]^+$.

2d (R^1 = OCH_3; R^2 = OCH_3): (colorless oil, 0.833g, 2.34 mmol, 72% yield). ^1H NMR (500 MHz, $CDCl_3$) δ 6.60 (s, 2H), 4.62 (s, 2H), 3.79 (s, 6H), 3.43 (t, *J* = 6.8 Hz, 2H), 2.62 (t, *J* = 7.3 Hz, 2H), 1.96 – 1.88 (m, 2H), 1.80 (p, *J* = 7.4 Hz, 2H), 1.59 (tdd, *J* = 10.0, 8.6, 5.0 Hz, 2H). $^{13}C\{^1H\}$ NMR (126 MHz, $CDCl_3$) δ 171.5, 152.2, 139.6, 127.8, 103.3, 65.4, 56.2, 33.7, 32.5, 27.6, 24.3. DART-MS (m/z) calculated for $C_{15}H_{22}O_5Br$: 361.06451; found 361.06420 $[M+H]^+$.

2e (R^1 = CH_3; R^2 = CH_3): (colorless oil, 0.933 g, 2.83 mmol, 68% yield). ^1H NMR (500 MHz, $CDCl_3$) δ 7.07 (s, 2H), 4.61 (s, 2H), 3.44 (t, *J* = 6.7 Hz, 2H), 2.63 (t, *J* = 7.5 Hz, 2H), 2.14 (s, 6H), 1.94 (dt, *J* = 14.9, 6.9 Hz, 2H), 1.83 (p, *J* = 7.6 Hz, 2H), 1.65 – 1.55 (m, 2H). $^{13}C\{^1H\}$ NMR (126 MHz, $CDCl_3$) δ 171.3, 147.7, 138.4, 130.4, 127.5, 127.4, 127.4, 65.1, 33.9, 33.6, 32.5, 27.9, 24.4, 16.6. DART-MS (m/z) calculated for $C_{15}H_{20}O_2Br$: 311.06412; found 311.06536 $[M+H]^+$.

2f: ^1H NMR (500 MHz, $CDCl_3$): (colorless oil, 0.609 g, 1.45 mmol, 67% yield). δ 7.06 (s, 2H), 4.60 (s, 2H), 3.90 (t, *J* = 6.3 Hz, 2H), 3.80 (t, *J* = 6.3 Hz, 2H), 3.70 – 3.62 (m, 8H), 3.46 (t, *J* = 6.3 Hz, 2H), 2.87 (t, *J* = 6.3 Hz, 2H), 2.15 (s, 6H), 1.67 (s, 1H). $^{13}C\{^1H\}$ NMR (126 MHz, $CDCl_3$) δ 169.6, 147.6, 138.5, 130.5, 127.4, 71.6, 70.8, 70.8, 70.7, 70.7, 35.1, 30.5, 16.5. DART-MS (m/z) calculated for $C_{18}H_{26}O_5Br$: 401.09581; found 401.09574 $[M+H]^+$.

Azidation

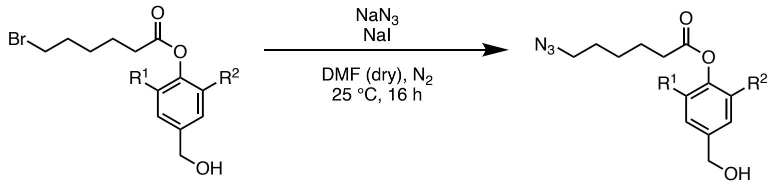

3a: R^1 = H; R^2 = H
3b: R^1 = H; R^2 = OCH_3
3c: R^1 = H; R^2 = CH_3
3d: R^1 = OCH_3; R^2 = OCH_3
3e: R^1 = CH_3; R^2 = CH_3

or

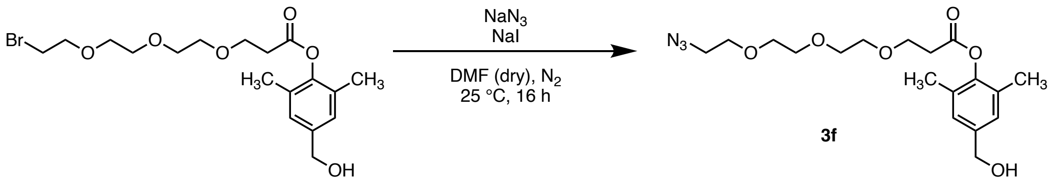

3f

General procedure. Compounds prepared using an adapted procedure from Vohidov et al.[1] To an oven-dried Schlenk flask was added NaN_3 (1.5 equiv), NaI (0.1 equiv), and the flask placed under an N_2 atmosphere. Dry DMF (5 mL) was added, and the reaction stirred at room temperature for 5 min. In a separate flask, the appropriate compound **2a–f** was dissolved in dry DMF (10 mL), and this solution was added to the reaction mixture via cannula. The reaction was left to stir at room temperature for 16 h. Following this, EtOAc (40 mL) was added, yielding a white precipitate, and the organic layer was washed with water (3 x 500 mL) and brine (1 x 500 mL). The organic layer was dried over Mg_2SO_4, filtered, and the solvent removed under reduced pressure. The crude compound was then purified using column chromatography (silica, 100% hexanes to 6:4 EtOAc:hexanes gradient).

Safety note. It is important to not use chlorinated solvents with reactions involving NaN_3. It is important to use large volumes of water to wash out unreacted NaN_3 from the organic layer. The sodium azide-containing aqueous waste should be kept at pH > 7.

3a (R^1 = H; R^2 = H): (colorless oil, 0.179 g, 0.680 mmol, 75% yield). ^{1}H NMR (500 MHz, $CDCl_3$) δ 7.39 – 7.32 (m, 2H), 7.08 – 7.02 (m, 2H), 4.65 (s, 2H), 3.30 (t, *J* = 6.8 Hz, 2H), 2.57 (t, *J* = 7.4 Hz, 2H), 1.97 (s, 1H), 1.78 (p, *J* = 7.5 Hz, 2H), 1.70 – 1.60 (m, 2H), 1.55 – 1.44 (m, 2H). $^{13}C\{^1H\}$ NMR (126 MHz, $CDCl_3$) δ 172.1, 150.1, 138.6, 128.1, 121.7, 64.7, 51.3, 34.2, 28.6, 26.3, 24.5. DART-MS (m/z) calculated for $C_{36}H_{48}N_7O_9$: 722.35080; found 722.34831 $[M+H]^+$. DART-MS (m/z) calculated for $C_{13}H_{18}N_3O_3$: 264.13427; found 264.13664 $[M+H]^+$.

3b (R^1 = H; R^2 = OCH_3): (colorless oil, 0.240 g, 0.818 mmol, 68% yield). ^{1}H NMR (500 MHz, $CDCl_3$) δ 7.03 – 6.93 (m, 2H), 6.89 (dd, *J* = 8.1, 1.9 Hz, 1H), 4.64 (s, 2H), 3.81 (s, 3H), 3.30 (t, *J* = 6.8 Hz, 2H), 2.59 (t, *J* = 7.4 Hz, 2H), 2.26 (s, 1H), 1.79 (p, *J* = 7.5 Hz, 2H), 1.72 – 1.64 (m, 2H), 1.57 – 1.44 (m, 2H).

$^{13}C\{^1H\}$ NMR (126 MHz, $CDCl_3$) δ 171.8, 151.2, 140.0, 139.1, 122.8, 119.1, 111.1, 65.1, 55.9, 51.4, 33.9, 28.7, 26.3, 24.6. DART-MS (m/z) calculated for $C_{14}H_{20}N3_7O_4$: 294.14483; found 294.14707 $[M+H]^+$.

3c (R^1 = H; R^2 = CH_3): (yellow oil, 0.444 g, 1.60 mmol, 80% yield). ^1H NMR (500 MHz, $CDCl_3$) δ 7.24 (d, *J* = 2.1 Hz, 1H), 7.19 (dd, *J* = 8.2, 2.2 Hz, 1H), 6.98 (d, *J* = 8.2 Hz, 1H), 4.64 (s, 2H), 3.31 (t, *J* = 6.8 Hz, 2H), 2.60 (t, *J* = 7.5 Hz, 2H), 2.17 (s, 3H), 1.81 (p, *J* = 7.5 Hz, 2H), 1.67 (dt, *J* = 14.6, 6.9 Hz, 3H), 1.58 – 1.46 (m, 2H). $^{13}C\{^1H\}$ NMR (126 MHz, $CDCl_3$) δ 171.8, 148.8, 138.7, 130.4, 130.0, 125.7, 122.1, 65.0, 51.6, 34.1, 28.7, 26.4, 24.7, 16.4. DART-MS (m/z) calculated for $C_{14}H_{20}N_3O_3$: 278.14992; found 278.15208 $[M+H]^+$.

3d (R^1 = OCH_3; R^2 = OCH_3): (colorless oil, 0.812 g, 2.51 mmol, 78% yield). ^1H NMR (500 MHz, $CDCl_3$) δ 6.63 (s, 2H), 4.66 (s, 2H), 3.82 (s, 6H), 3.30 (t, *J* = 6.9 Hz, 2H), 2.63 (t, *J* = 7.3 Hz, 2H), 1.81 (p, *J* = 7.4 Hz, 2H), 1.73 – 1.61 (m, 2H), 1.58 – 1.44 (m, 2H). ^{13}C NMR (126 MHz, $CDCl_3$) δ 171.4, 152.4, 139.5, 128.0, 103.4, 103.4, 65.6, 56.3, 51.5, 33.8, 28.7, 26.3, 24.7. DART-MS (m/z) calculated for $C_{15}H_{22}N_3O_5$: 324.15540; found 324.15686 $[M+H]^+$.

3e (R^1 = CH_3; R^2 = CH_3): (colorless oil, 0.711 g, 2.43 mmol, 81% yield). ^1H NMR (500 MHz, $CDCl_3$) δ 7.06 (s, 2H), 4.60 (s, 2H), 3.31 (t, *J* = 6.8 Hz, 2H), 2.63 (t, *J* = 7.5 Hz, 2H), 2.14 (s, 6H), 1.83 (p, *J* = 7.5 Hz, 2H), 1.72 – 1.63 (m, 2H), 1.58 – 1.46 (m, 2H). $^{13}C\{^1H\}$ NMR (126 MHz, $CDCl_3$) δ 171.3, 147.7, 138.5, 130.4, 127.4, 65.0, 51.3, 33.9, 28.7, 26.5, 24.7, 16.5. DART-MS (m/z) calculated for $C_{15}H_{22}N_3O_3$: 292.16557; found 292.16693 $[M+H]^+$.

3f: ^1H NMR (500 MHz, $CDCl_3$): (colorless oil, 0.459 g, 1.20 mmol, 84% yield). δ 7.06 (s, 2H), 4.61 (s, 2H), 3.90 (t, *J* = 6.3 Hz, 2H), 3.71 – 3.63 (m, 10H), 3.37 (t, *J* = 5.1 Hz, 2H), 2.88 (t, *J* = 6.3 Hz, 2H), 2.15 (s, 6H), 1.63 (s, 1H). $^{13}C\{^1H\}$ NMR (126 MHz, $CDCl_3$) δ 169.6, 147.7, 138.5, 130.5, 127.4, 70.85, 70.8, 70.7, 66.9, 65.1, 50.8, 35.1, 16.5. DART-MS (m/z) calculated for $C_{18}H_{28}N_3O_6$: 382.19726; found 382.19789 $[M+H]^+$.

<u>Carbonyl</u>

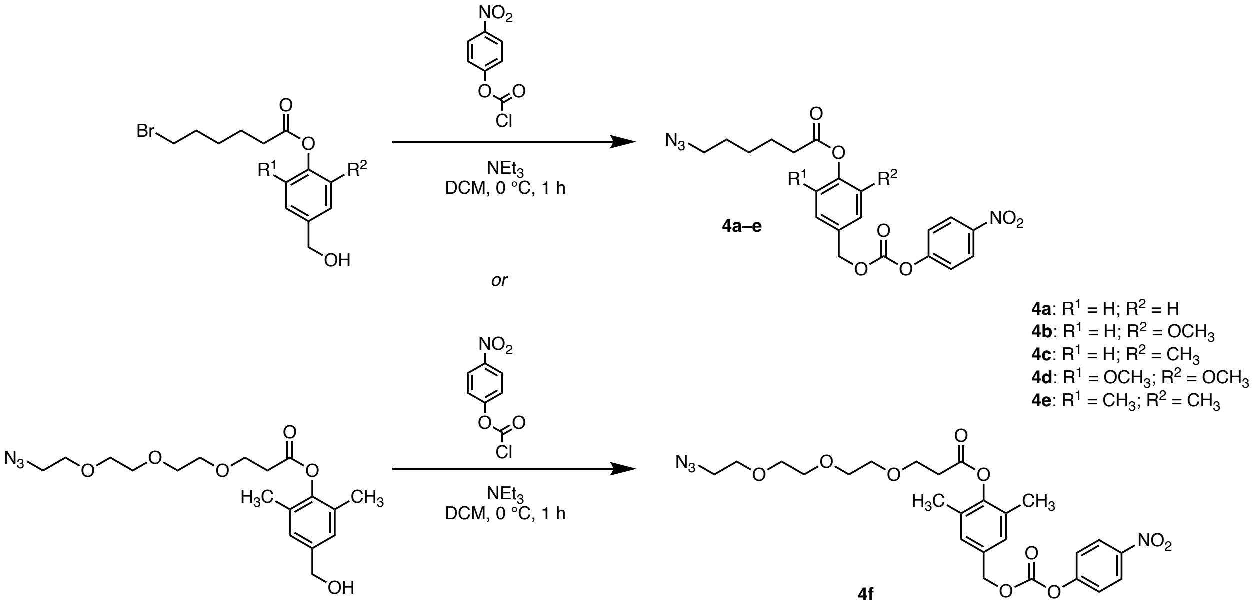

General procedure. Compounds prepared using an adapted procedure from Vohidov et al.[1] A round-bottomed flask was charged with the appropriate compound **3a–f** (1.0 equiv), dry DCM (50 mL), and the flask cooled to 0 °C. To this was added NEt_3 (3.0 equiv) and the solution left to stir at 0 °C for 5 min. Separately, a solution of 4-nitrophenylchloroformate (1.05 equiv) in DCM (20 mL) is prepared, and added slowly to the reaction mixture. The solution was left to stir for 1 h at 0 °C, after which it turned a bright yellow color. The reaction mixture was then washed with saturated aqueous $NaHCO_3$ solution (3 x 150 mL), the resulting organic layer dried with $MgSO_4$, filtered, and the DCM removed *in vacuo*. The crude compound was then purified using column chromatography (silica, 100% hexanes to 6:4 EtOAc:hexanes gradient).

4a (R^1 = H; R^2 = H): (off-white powder, 0.2038 g, 0.475 mmol, 69% yield). ^{1}H NMR (500 MHz, $CDCl_3$) δ 8.29 – 8.22 (m, 2H), 7.50 – 7.43 (m, 2H), 7.41 – 7.34 (m, 2H), 7.16 – 7.10 (m, 2H), 5.28 (s, 2H), 3.31 (t, *J* = 6.8 Hz, 2H), 2.59 (t, *J* = 7.4 Hz, 2H), 1.79 (p, *J* = 7.5 Hz, 2H), 1.71 – 1.61 (m, 2H), 1.57 – 1.45 (m, 2H). $^{13}C\{^1H\}$ NMR (126 MHz, $CDCl_3$) δ 171.9, 155.6, 152.5, 151.3, 145.5, 131.9, 130.2, 125.4, 122.1, 121.9, 70.4, 51.3, 34.2, 28.7, 26.3, 24.5. DART-MS (m/z) calculated for $C_{20}H_{19}N_4O_8$: 443.11974; found 443.12156 $[M–H+O]^-$.

4b (R^1 = H; R^2 = OCH_3): (yellow powder, 0.373 g, 0.81 mmol, 62% yield). ^{1}H NMR (500 MHz, $CDCl_3$) δ 8.30 – 8.21 (m, 2H), 7.42 – 7.34 (m, 2H), 7.09 – 6.98 (m, 3H), 5.26 (s, 2H), 3.84 (s, 3H), 3.30 (t, *J* = 6.9 Hz, 2H), 2.61 (t, *J* = 7.3 Hz, 2H), 1.80 (p, *J* = 7.3 Hz, 2H), 1.71 – 1.63 (m, 2H), 1.56 – 1.47 (m, 2H). $^{13}C\{^1H\}$ NMR (126 MHz, $CDCl_3$) δ 171.5, 155.5, 152.5, 151.4, 145.5, 140.4, 133.0, 125.4, 123.1, 121.9,

121.3, 112.9, 70.6, 56.0, 56.0, 51.3, 44.9, 33.8, 28.6, 26.2, 24.5. DART-MS (m/z) calculated for $C_{21}H_{21}N_4O_9$: 473.13030; found 473.13219 $[M–H+O]^–$.

4c (R^1 = H; R^2 = CH_3): (yellow powder, 0.453 g, 1.02 mmol, 64% yield). ^{1}H NMR (500 MHz, $CDCl_3$) δ 8.30 – 8.24 (m, 2H), 7.42 – 7.34 (m, 2H), 7.34 – 7.28 (m, 2H), 7.04 (d, *J* = 8.2 Hz, 1H), 5.25 (s, 2H), 3.32 (t, *J* = 6.8 Hz, 2H), 2.62 (t, *J* = 7.5 Hz, 2H), 2.20 (s, 3H), 1.81 (p, *J* = 7.6 Hz, 2H), 1.72 – 1.64 (m, 2H), 1.56 – 1.49 (m, 2H). $^{13}C\{^1H\}$ NMR (126 MHz, $CDCl_3$) δ 171.7, 155.6, 152.6, 150.0, 145.6, 132.0, 131.9, 130.9, 127.7, 125.4, 122.5, 121.9, 70.5, 51.3, 34.10, 34.1, 28.7, 26.4, 24.6, 16.4. DART-MS (m/z) calculated for $C_{21}H_{21}N_4O_8$: 457.13539; found 457.13701 $[M–H+O]^–$.

4d (R^1 = OCH_3; R^2 = OCH_3): (white powder, 0.668 g, 1.36 mmol, 72% yield). ^{1}H NMR (500 MHz, $CDCl_3$) δ 8.31 – 8.25 (m, 2H), 7.41 – 7.36 (m, 2H), 6.69 (s, 2H), 5.24 (s, 2H), 3.84 (s, 6H), 3.31 (t, *J* = 6.8 Hz, 2H), 2.64 (t, *J* = 7.3 Hz, 2H), 1.87 – 1.73 (m, 2H), 1.71 – 1.62 (m, 2H), 1.60 – 1.48 (m, 2H). $^{13}C\{^1H\}$ NMR (126 MHz, $CDCl_3$) δ 171.2, 155.6, 152.5, 152.5, 145.6, 132.5, 129.2, 125.5, 121.9, 105.5, 71.1, 56.3, 51.4, 33.7, 28.7, 26.2, 24.7. DART-MS (m/z) calculated for $C_{22}H_{23}N_4O_{10}$: 503.14087; found 503.14268 $[M–H+O]^–$.

4e (R^1 = CH_3; R^2 = CH_3): (pale yellow powder, 0.658 g, 1.44 mmol, 60% yield). ^{1}H NMR (500 MHz, $CDCl_3$) δ 8.30 – 8.24 (m, 2H), 7.42 – 7.34 (m, 2H), 7.15 (s, 2H), 5.21 (s, 2H), 3.32 (t, *J* = 6.8 Hz, 2H), 2.64 (t, *J* = 7.5 Hz, 2H), 2.17 (s, 6H), 1.83 (p, *J* = 7.5 Hz, 2H), 1.73 – 1.64 (m, 2H), 1.59 – 1.48 (m, 2H). $^{13}C\{^1H\}$ NMR (126 MHz, $CDCl_3$) δ 171.2, 155.7, 152.6, 148.8, 145.5, 131.7, 130.9, 129.3, 129.3, 125.4, 121.9, 70.7, 51.3, 33.9, 28.7, 26.5, 24.7, 16.5. DART-MS (m/z) calculated for $C_{22}H_{23}N_4O_8$: 471.15104; found 471.15269 $[M–H+O]^–$.

4f: (pale yellow oil, 0.45 g, 0.82 mmol, 70% yield). ^{1}H NMR (500 MHz, $CDCl_3$) δ 8.30 – 8.22 (m, 2H), 7.42 – 7.34 (m, 2H), 7.14 (s, 2H), 5.21 (s, 2H), 3.95 – 3.86 (m, 2H), 3.72 – 3.60 (m, 10H), 3.38 (t, *J* = 5.1 Hz, 2H), 2.88 (q, *J* = 6.5 Hz, 2H), 2.17 (s, 6H). $^{13}C\{^1H\}$ NMR (126 MHz, $CDCl_3$) δ 169.5, 147.5, 138.4, 130.4, 127.3, 70.7, 70.7, 70.5, 70.1, 50.7, 35.0, 16.4. DART-MS (m/z) calculated for $C_{25}H_{29}N_4O_{11}$: 561.18273; found 561.18445 $[M–H+O]^–$.

Resiquimod

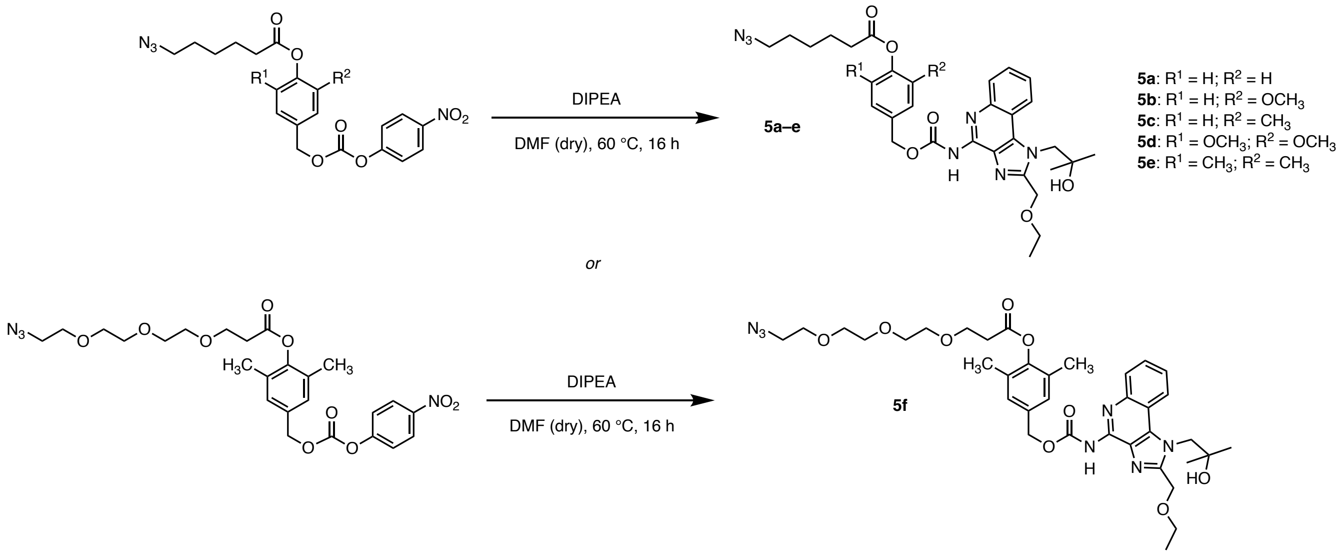

General procedure. Compounds prepared using an adapted procedure from Vohidov et al.[1] A 40 mL vial was charged with a stir bar, Resiquimod (R848, 1.0 equiv) and dry DMF (2 mL). Diisopropylethylamine (DIPEA, 2.0 equiv) was added dropwise, and the solution stirred at room temperature for 5 min. To this mixture was added the appropriate species **4a–f** (1.1 equiv) in one portion, and the reaction mixture immediately turned bright yellow. The vial was sealed and heated at 60 °C for 16 h. The reaction mixture was then cooled, and EtOAc (40 mL) added. The organic layer was washed with H_2O (3 x 100 mL) and brine (1 x 100 mL), dried over Mg_2SO_4, filtered, and the EtOAc removed *in vacuo*. The crude compound was purified using column chromatography (silica, 100% DCM to 95:5 DCM:MeOH gradient).

5a (R^1 = H; R^2 = H): (white powder, 0.2 g, 0.34 mmol, 80% yield). ^{1}H NMR (600 MHz, $CDCl_3$) δ 8.16 (dt, *J* = 8.4, 1.5 Hz, 2H), 7.61 (ddd, *J* = 8.4, 7.0, 1.3 Hz, 1H), 7.52 – 7.46 (m, 3H), 7.12 – 7.05 (m, 2H), 5.31 (s, 2H), 4.84 (d, *J* = 69.7 Hz, 4H), 3.64 (q, *J* = 7.0 Hz, 2H), 3.31 (t, *J* = 6.9 Hz, 2H), 3.23 (s, 1H), 2.58 (t, *J* = 7.4 Hz, 2H), 1.79 (p, *J* = 7.5 Hz, 2H), 1.67 (p, *J* = 7.0 Hz, 2H), 1.56 – 1.47 (m, 2H), 1.33 (s, 7H), 1.24 (t, *J* = 7.0 Hz, 3H). $^{13}C\{^1H\}$ NMR (151 MHz, $CDCl_3$) δ 172.0, 151.1, 150.7, 135.9, 133.7, 129.8, 127.9, 127.6, 124.9, 121.8, 120.2, 116.7, 71.6, 66.9, 66.8, 65.1, 56.6, 51.4, 34.3, 29.9, 28.7, 26.4, 24.5, 15.1. DART-MS (m/z) calculated for $C_{31}H_{38}N_7O_6$: 604.28781; found 604.28500 $[M+H]^+$.

5b (R^1 = H; R^2 = OCH_3): (white powder, 0.259 g, 0.4 mmol, 75% yield). ^{1}H NMR (600 MHz, $CDCl_3$) δ 8.22 – 8.12 (m, 2H), 7.63 (ddd, *J* = 8.3, 7.0, 1.3 Hz, 1H), 7.50 (ddd, *J* = 8.4, 7.0, 1.4 Hz, 1H), 7.12 – 7.03 (m, 4H), 5.31 (s, 2H), 4.86 (d, *J* = 68.1 Hz, 4H), 3.87 (s, 3H), 3.67 (q, *J* = 7.0 Hz, 2H), 3.33 (t, *J* = 6.9 Hz, 2H), 3.18 (s, 1H), 2.63 (td, *J* = 7.4, 1.6 Hz, 2H), 1.82 (q, *J* = 7.6 Hz, 2H), 1.69 (h, *J* = 7.2 Hz, 2H), 1.55 (tt, *J* = 10.1, 6.4 Hz, 2H), 1.35 (s, 7H), 1.27 (t, *J* = 7.1 Hz, 3H). $^{13}C\{^1H\}$ NMR (151 MHz, $CDCl_3$) δ 171.6, 151.3, 150.9, 139.8, 135.6, 135.0, 127.8, 124.7, 122.9, 120.9, 120.1, 116.7, 112.7, 71.6, 67.1, 66.8, 65.1, 56.6,

56.1, 51.4, 45.0, 33.9, 32.3, 28.7, 28.8, 26.4, 26.3, 24.6, 24.4, 15.1. DART-MS (m/z) calculated for $C_{32}H_{40}N_7O_7$: 634.29837; found 634.29566 $[M+H]^+$.

5c (R^1 = H; R^2 = CH_3): (white powder, 0.221 g, 0.358 mmol, 65% yield). ^{1}H NMR (600 MHz, $CDCl_3$) δ 8.18 – 8.08 (m, 2H), 7.58 (ddd, *J* = 8.3, 7.0, 1.3 Hz, 1H), 7.46 (ddd, *J* = 8.3, 7.0, 1.3 Hz, 1H), 7.36 (d, *J* = 2.3 Hz, 1H), 7.31 (dd, *J* = 8.2, 2.2 Hz, 1H), 7.00 (d, *J* = 8.2 Hz, 1H), 5.26 (s, 2H), 4.81 (d, *J* = 65.8 Hz, 4H), 3.62 (q, *J* = 7.1 Hz, 2H), 3.35 – 3.24 (m, 3H), 2.60 (t, *J* = 7.5 Hz, 2H), 2.18 (s, 3H), 1.80 (p, *J* = 7.6 Hz, 2H), 1.67 (p, *J* = 7.0 Hz, 2H), 1.55 – 1.48 (m, 2H), 1.31 (s, 7H), 1.23 (t, *J* = 7.0 Hz, 3H). $^{13}C\{^1H\}$ NMR (151 MHz, $CDCl_3$) δ 171.6, 150.7, 149.8, 149.2, 135.5, 133.7, 131.3, 130.3, 127.6, 127.1, 125.4, 124.5, 122.0, 119.9, 116.6, 71.5, 66.7, 66.6, 64.9, 56.4, 51.2, 34.0, 28.6, 27.9, 26.3, 24.5, 16.2, 14.9. DART-MS (m/z) calculated for $C_{32}H_{40}N_7O_6$: 618.30346; found 618.29979 $[M+H]^+$.

5d (R^1 = OCH_3; R^2 = OCH_3): (white powder, 0.249 g, 0.375 mmol, 73% yield). ^{1}H NMR (600 MHz, $CDCl_3$) δ 8.58 (s, 1H), 8.12 (t, *J* = 9.7 Hz, 2H), 7.55 (t, *J* = 7.8 Hz, 1H), 7.43 (t, *J* = 7.7 Hz, 1H), 6.69 (s, 2H), 5.22 (s, 2H), 4.77 (d, *J* = 68.4 Hz, 4H), 3.80 (s, 6H), 3.57 (q, *J* = 7.1 Hz, 2H), 3.27 (t, *J* = 6.9 Hz, 2H), 2.61 (t, *J* = 7.4 Hz, 2H), 1.79 (q, *J* = 7.6 Hz, 2H), 1.64 (p, *J* = 7.1 Hz, 2H), 1.57 – 1.49 (m, 2H), 1.28 (s, 7H), 1.19 (t, *J* = 7.1 Hz, 3H). $^{13}C\{^1H\}$ NMR (151 MHz, $CDCl_3$) δ 171.22, 152.2, 151.5, 150.9, 144.5, 144.0, 135.5, 134.3, 130.3, 128.5, 127.5, 127.4, 124.6, 120.0, 116.8, 105., 71.51, 67.3, 66.7, 65.0, 56.3, 56.3, 56.2, 51.4, 51.3, 33.7, 28.6, 26.1, 24.6, 15.0. DART-MS (m/z) calculated for $C_{33}H_{42}N_7O_8$: 664.30751; found 664.30894 $[M+H]^+$.

5e (R^1 = CH_3; R^2 = CH_3): (white powder, 0.239 g, 0.378 mmol, 70% yield). ^{1}H NMR (600 MHz, $CDCl_3$) δ 8.11 (d, *J* = 8.3 Hz, 2H), 7.56 (t, *J* = 7.9 Hz, 1H), 7.48 – 7.41 (m, 1H), 7.17 (s, 2H), 7.03 (s, 1H), 5.21 (s, 2H), 4.78 (d, *J* = 67.1 Hz, 4H), 3.64 – 3.52 (m, 2H), 3.44 (t, *J* = 10.5 Hz, 1H), 3.29 (t, *J* = 6.9 Hz, 3H), 2.61 (td, *J* = 7.6, 3.7 Hz, 3H), 2.12 (d, *J* = 17.7 Hz, 9H), 1.87 – 1.76 (m, 2H), 1.66 (p, *J* = 7.1 Hz, 2H), 1.51 (p, *J* = 7.8 Hz, 2H), 1.27 (d, *J* = 25.7 Hz, 7H), 1.20 (td, *J* = 7.1, 2.0 Hz, 3H). $^{13}C\{^1H\}$ NMR (151 MHz, $CDCl_3$) δ 171.3, 171.2, 150.9, 148.1, 147.5, 138.6, 138.6, 135.6, 133.5, 130.4, 130.2, 130.2, 128.8, 127.6, 127.6, 127.3, 124.5, 120.1, 116.7, 71.52, 66.8, 66.7, 65.0, 64.8, 56.4, 51.3, 33.9, 28.6, 27.9, 26.4, 24.7, 16.5, 15.0. DART-MS (m/z) calculated for $C_{33}H_{42}N_7O_6$: 632.31724; found 632.31911 $[M+H]^+$.

5f: (pale yellow powder, 0.231 g, 0.319 mmol, 68% yield). ^{1}H NMR (600 MHz, $CDCl_3$) δ 8.09 (dt, *J* = 8.7, 4.4 Hz, 2H), 7.53 (ddd, *J* = 8.3, 6.9, 1.3 Hz, 1H), 7.41 (ddd, *J* = 8.3, 6.9, 1.3 Hz, 1H), 7.15 (s, 2H), 5.19 (s, 2H), 4.75 (d, *J* = 65.6 Hz, 4H), 3.87 (t, *J* = 6.3 Hz, 2H), 3.68 – 3.61 (m, 10H), 3.60 – 3.49 (m, 3H), 3.34 (t, *J* = 5.0 Hz, 2H), 2.85 (t, *J* = 6.3 Hz, 2H), 2.13 (s, 6H), 1.27 (s, 7H), 1.17 (t, *J* = 7.0 Hz, 3H). $^{13}C\{^1H\}$ NMR (151 MHz, $CDCl_3$) δ 169.4, 150.9, 148.1, 135.4, 133.5, 130.5, 130.4, 128.7, 128.7, 127.5, 124.4,

120.0, 116.6, 71.5, 70.7, 70.6, 70.5, 70.1, 66.7, 66.7, 66.6, 65.0, 56.25, 50.7, 35.0, 27.9, 16.3, 14.9. DART-MS (m/z) calculated for $C_{36}H_{48}N_7O_9$: 722.35080; found 722.34831 $[M+H]^+$.

NMR Spectra

Esterification

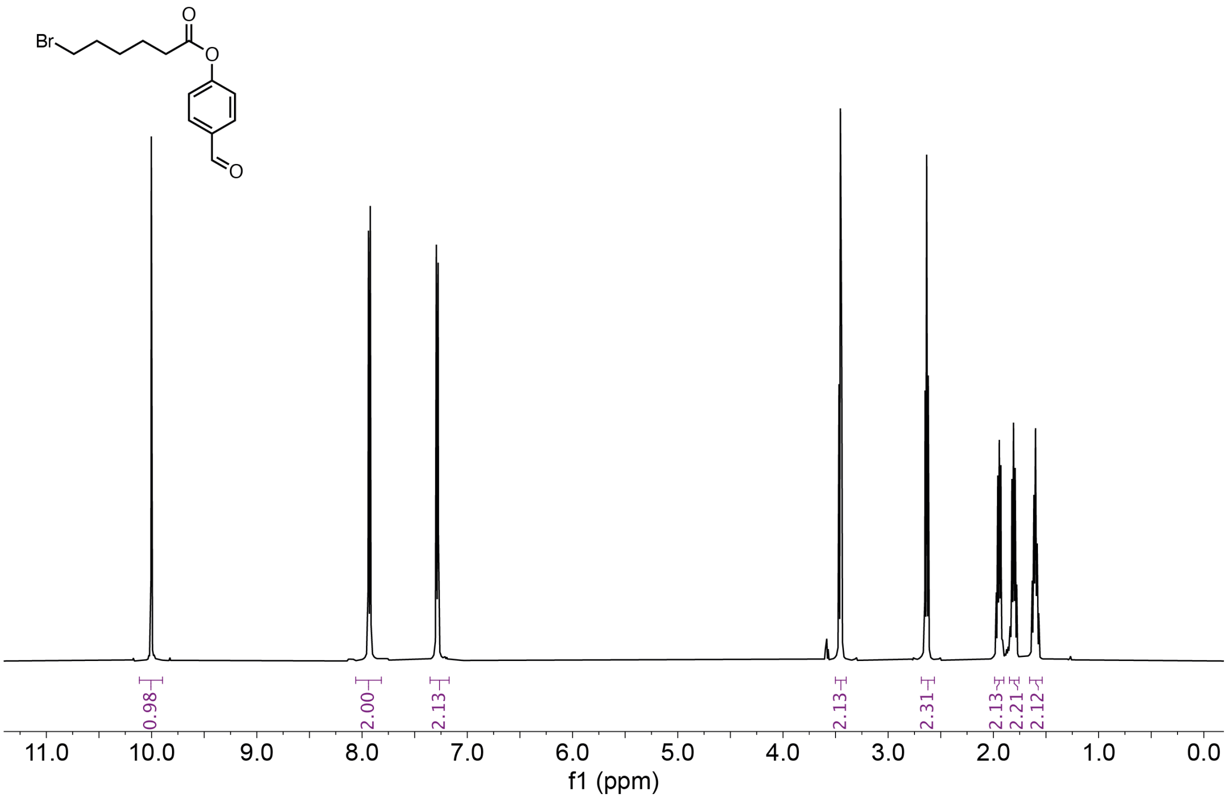

Fig. S2A-I. ^{1}H NMR spectrum of **1a** ($CDCl_3$, 500 MHz, 25 °C).

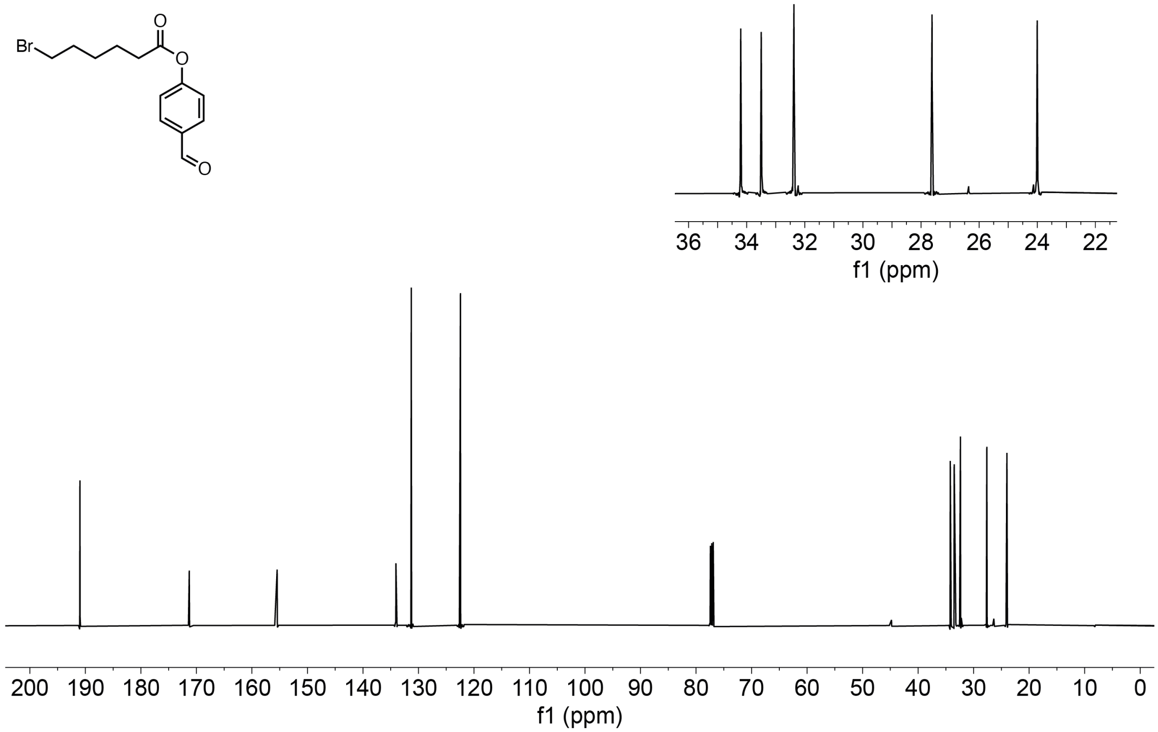

Fig. S2A-II. $^{13}C\{^{1}H\}$ NMR spectrum of **1a** ($CDCl_3$, 126 MHz, 25 °C).

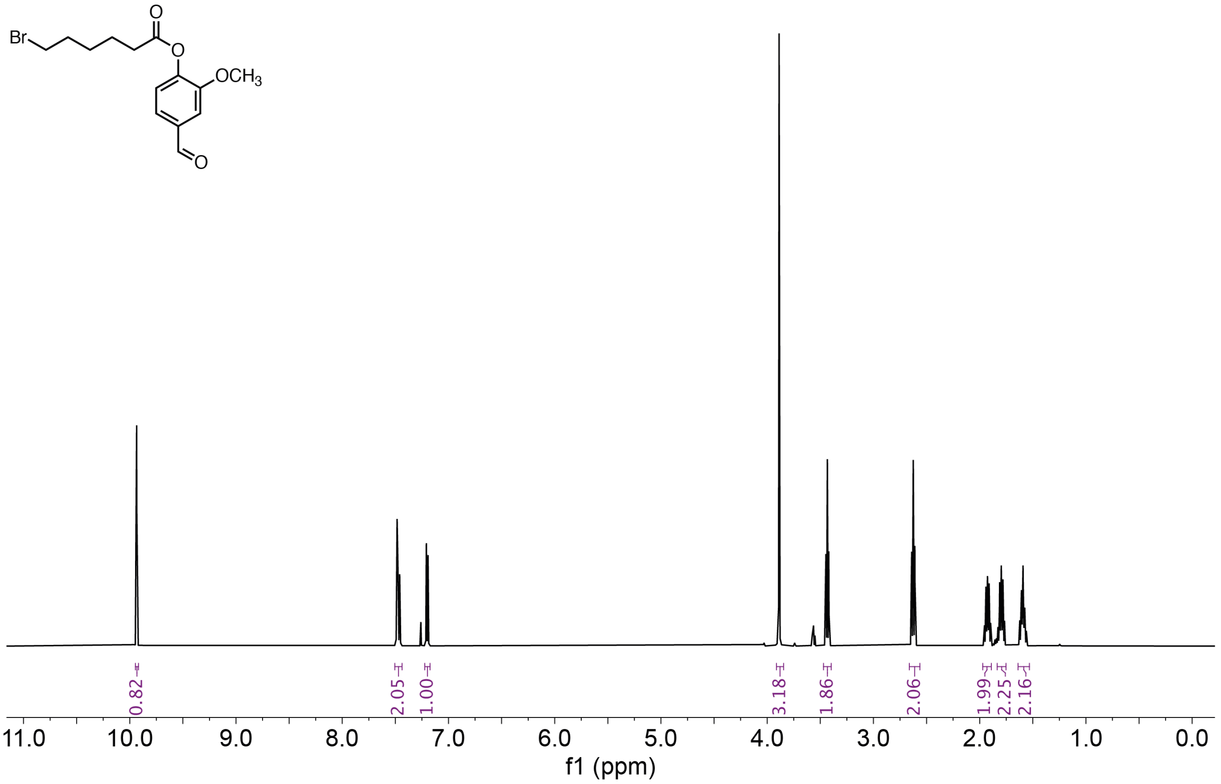

Fig. S2B-I. ^{1}H NMR spectrum of **1b** ($CDCl_3$, 500 MHz, 25 °C).

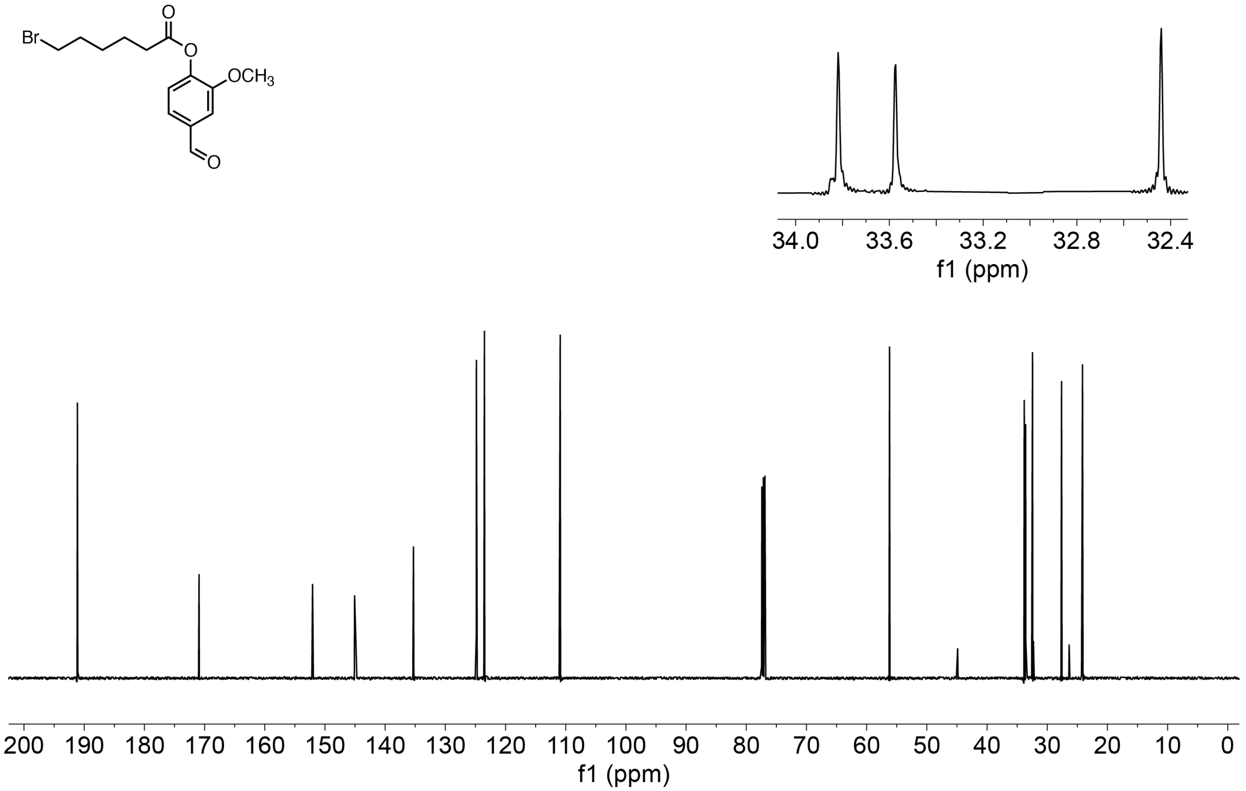

Fig. S2B-II. $^{13}C\{^{1}H\}$ NMR spectrum of **1b** ($CDCl_3$, 126 MHz, 25 °C).

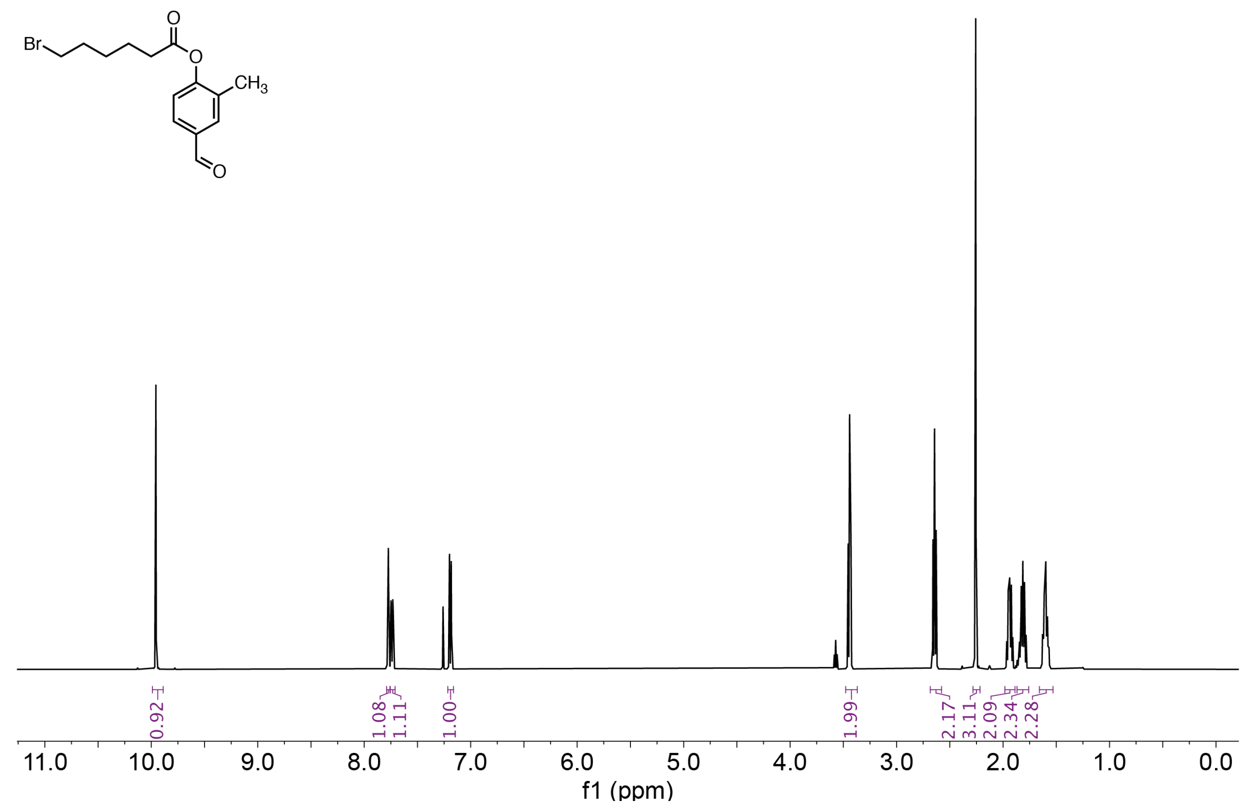

Fig. S2C-I. ^{1}H NMR spectrum of **1c** ($CDCl_3$, 500 MHz, 25 °C).

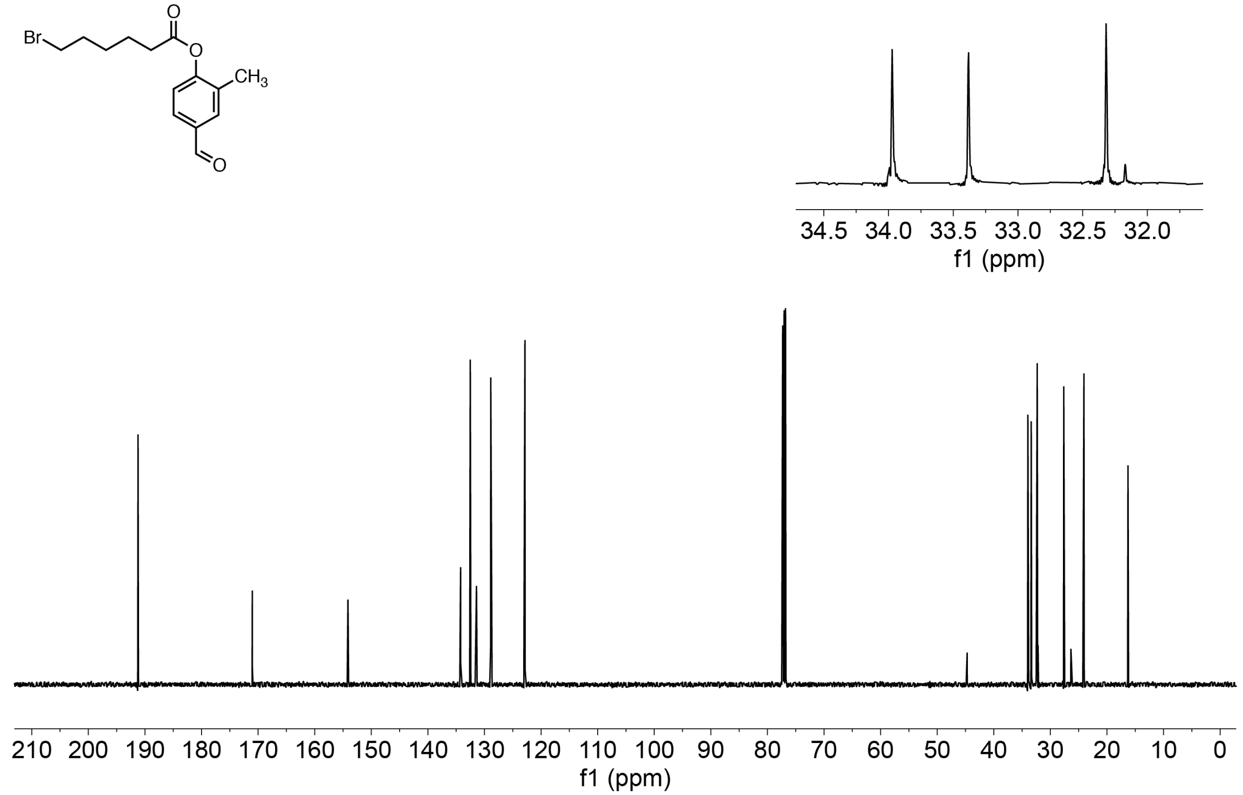

Fig. S2C-II. $^{13}C\{^{1}H\}$ NMR spectrum of **1c** ($CDCl_3$, 126 MHz, 25 °C).

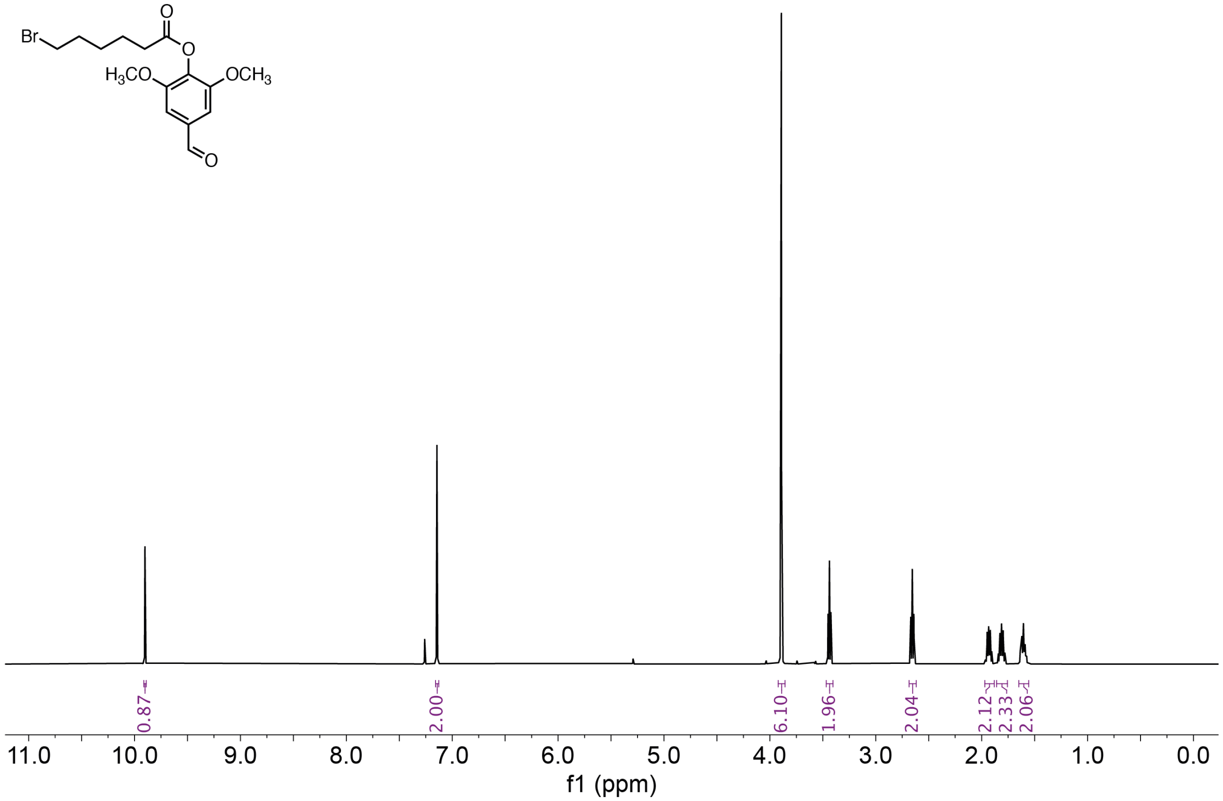

Fig. S2D-I. ^{1}H NMR spectrum of **1d** ($CDCl_3$, 500 MHz, 25 °C).

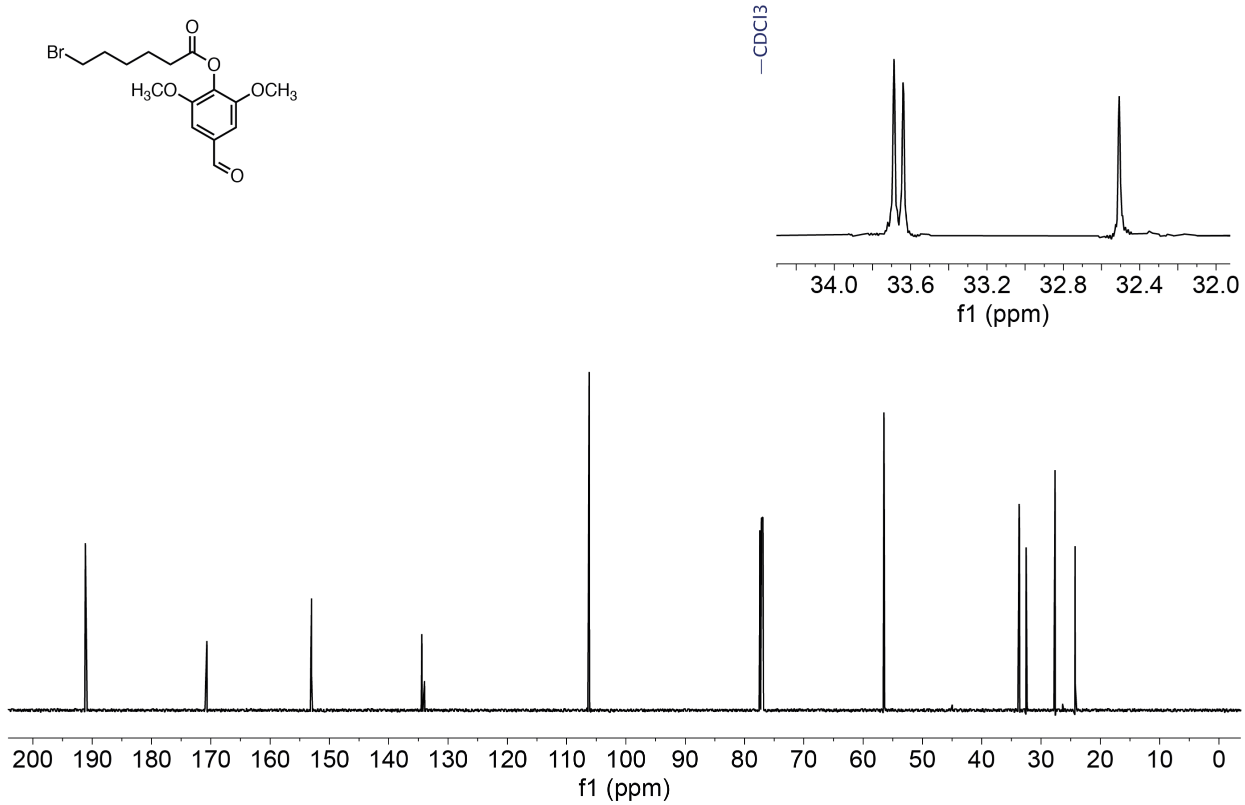

Fig. S2D-II. $^{13}C\{^{1}H\}$ NMR spectrum of **1d** ($CDCl_3$, 126 MHz, 25 °C).

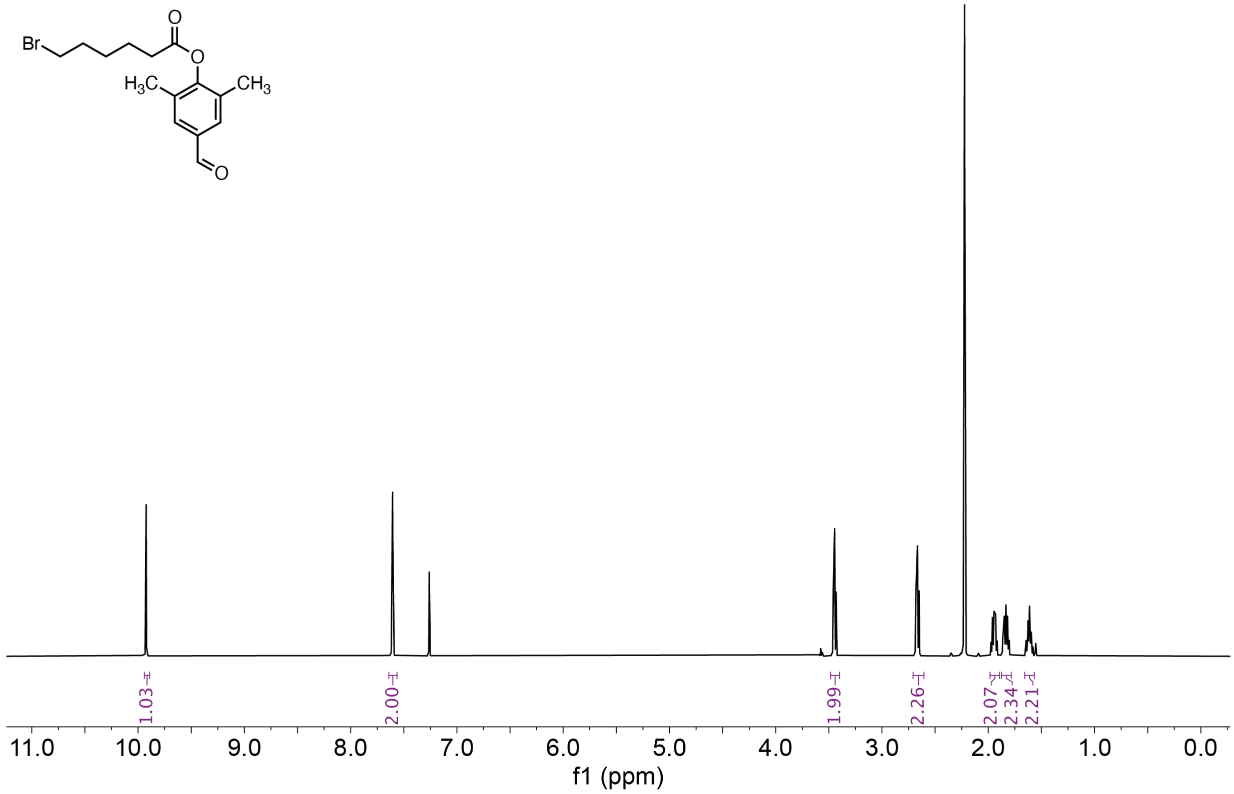

Fig. S2E-I. ^{1}H NMR spectrum of **1e** ($CDCl_3$, 500 MHz, 25 °C).

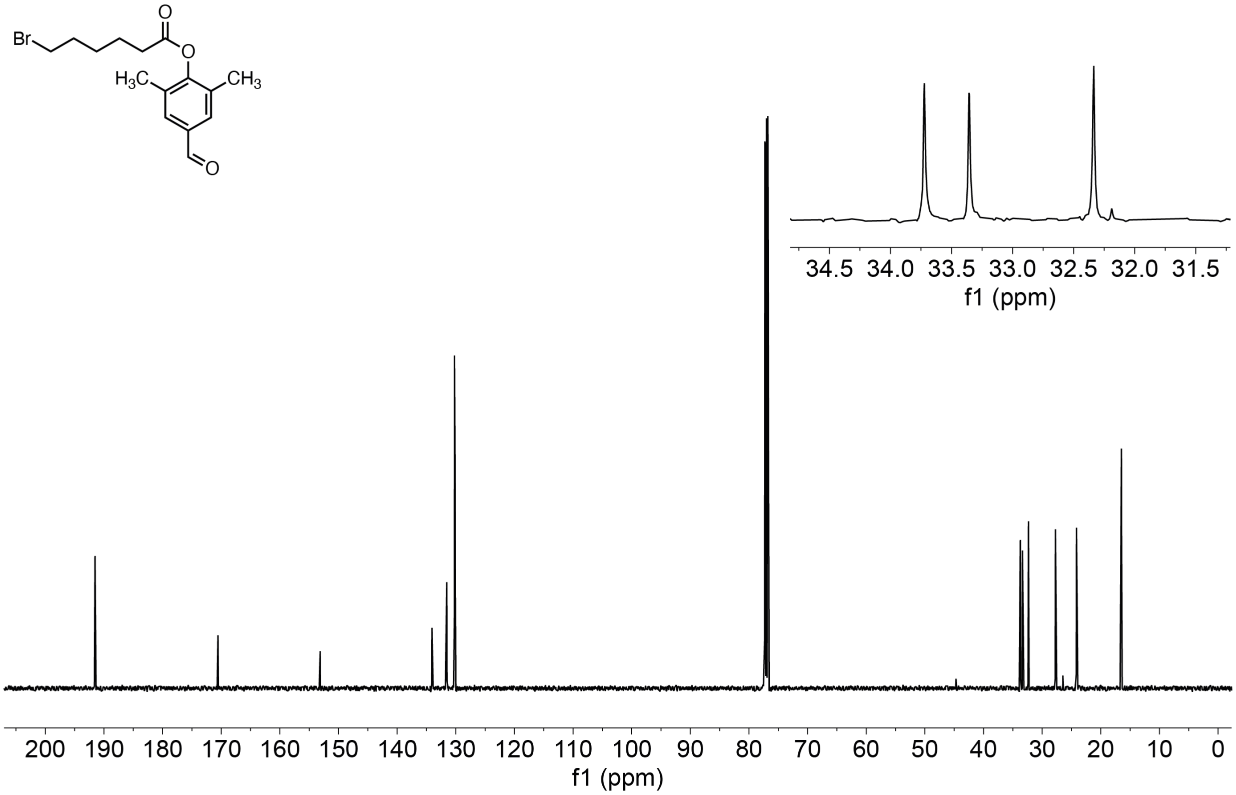

Fig. S2E-II. $^{13}C\{^{1}H\}$ NMR spectrum of **1e** ($CDCl_3$, 126 MHz, 25 °C).

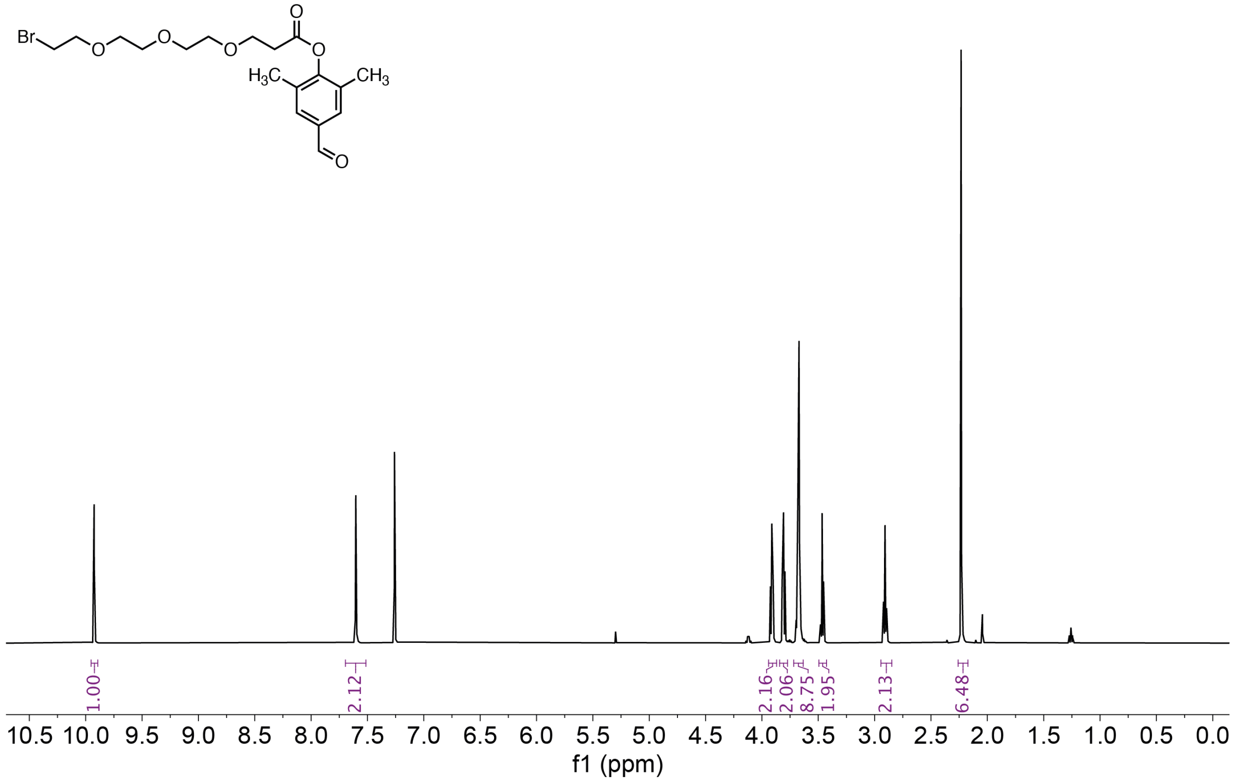

Fig. S2F-I. ^{1}H NMR spectrum of **1f** ($CDCl_3$, 500 MHz, 25 °C).

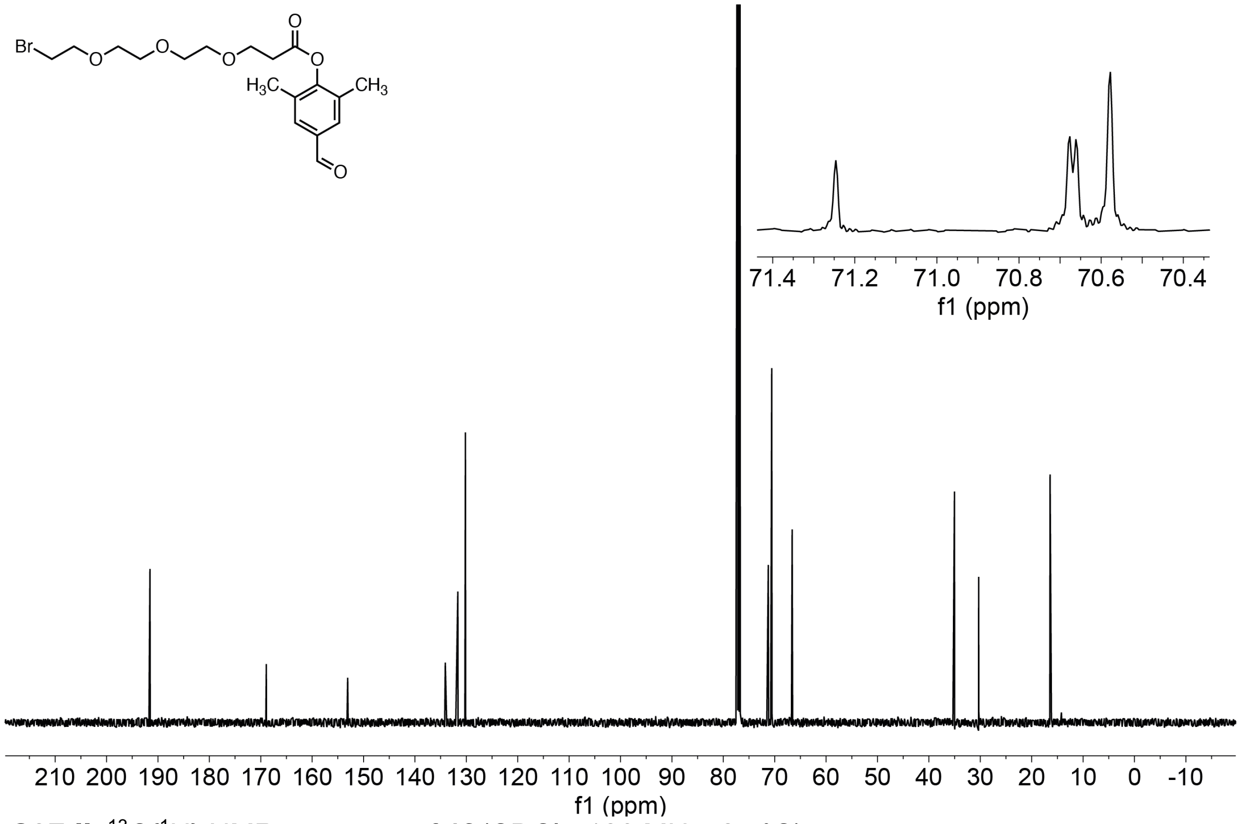

Fig. S2F-II. $^{13}C\{^{1}H\}$ NMR spectrum of **1f** ($CDCl_3$, 126 MHz, 25 °C).

Reduction

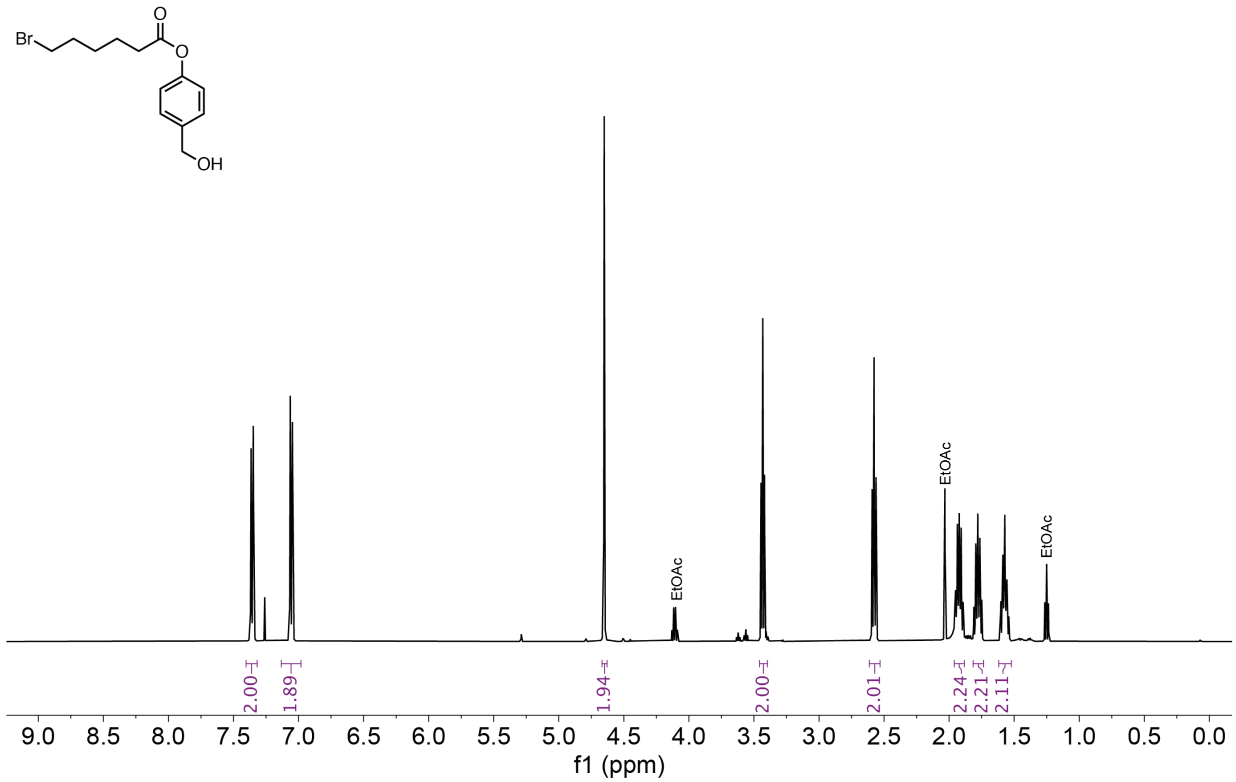

Fig. S3A-I. ^{1}H NMR spectrum of **2a** ($CDCl_3$, 500 MHz, 25 °C).

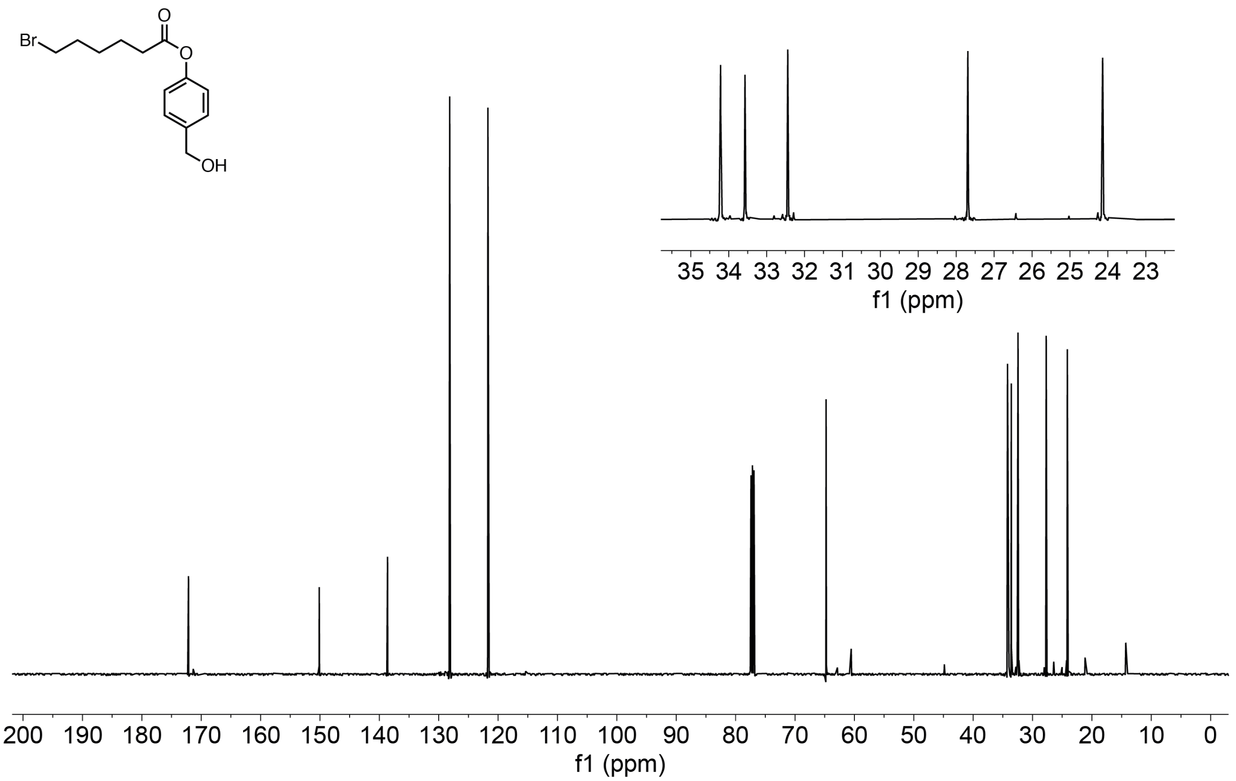

Fig. S3A-II. $^{13}C\{^{1}H\}$ NMR spectrum of **2a** ($CDCl_3$, 126 MHz, 25 °C).

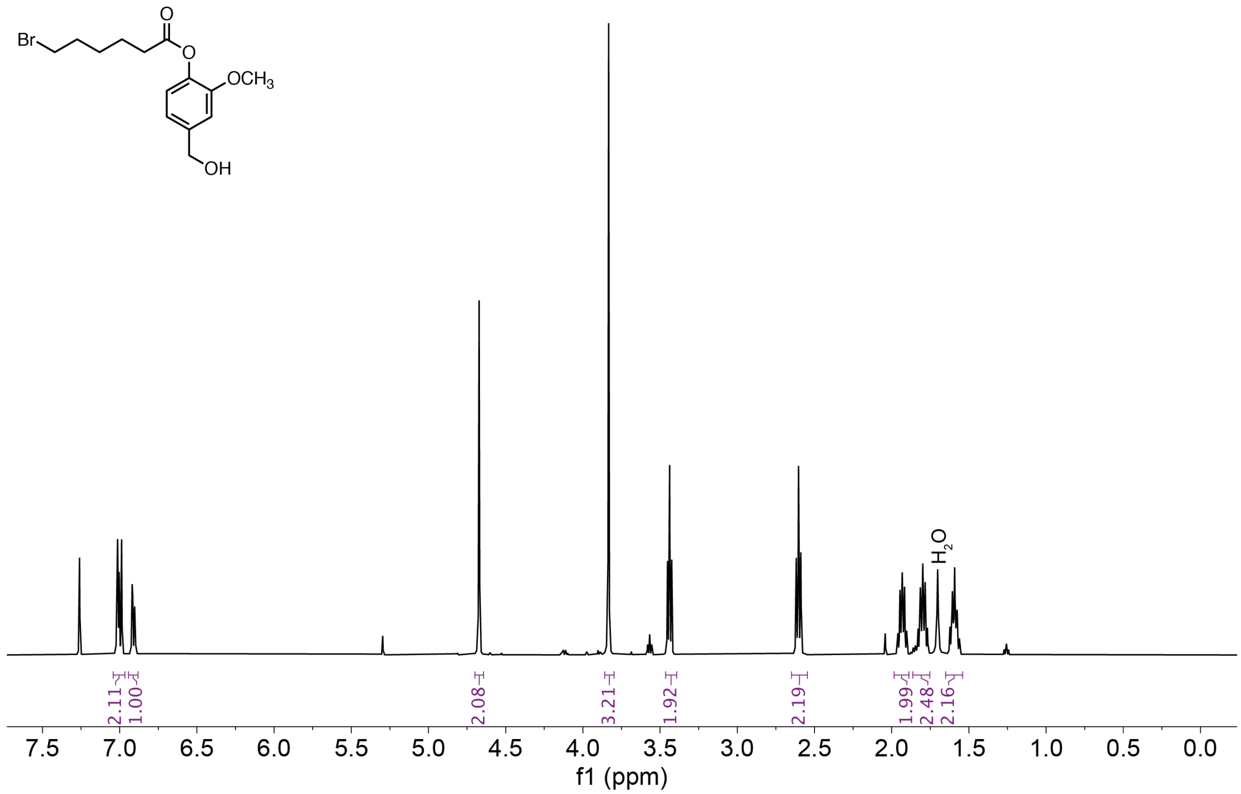

Fig. S3B-I. ^{1}H NMR spectrum of **2b** ($CDCl_3$, 500 MHz, 25 °C).

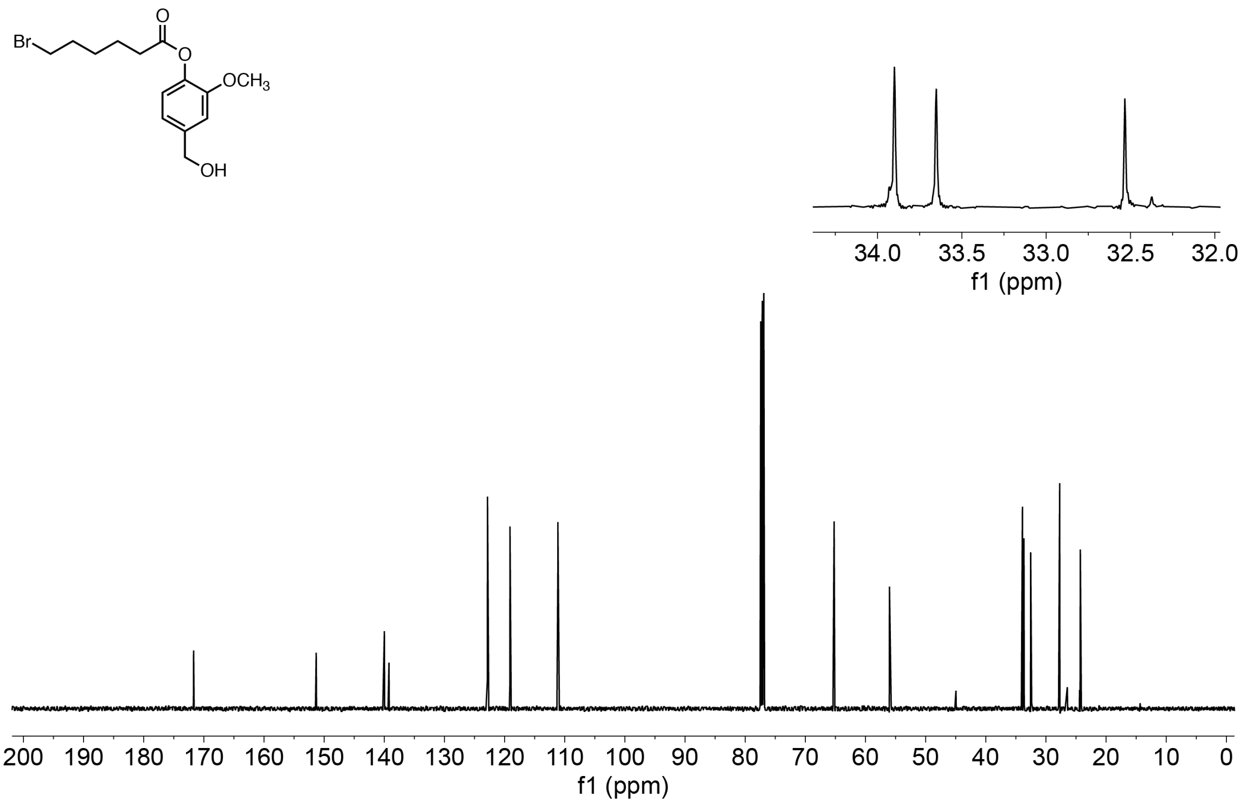

Fig. S3B-II. $^{13}C\{^{1}H\}$ NMR spectrum of **2b** ($CDCl_3$, 126 MHz, 25 °C).

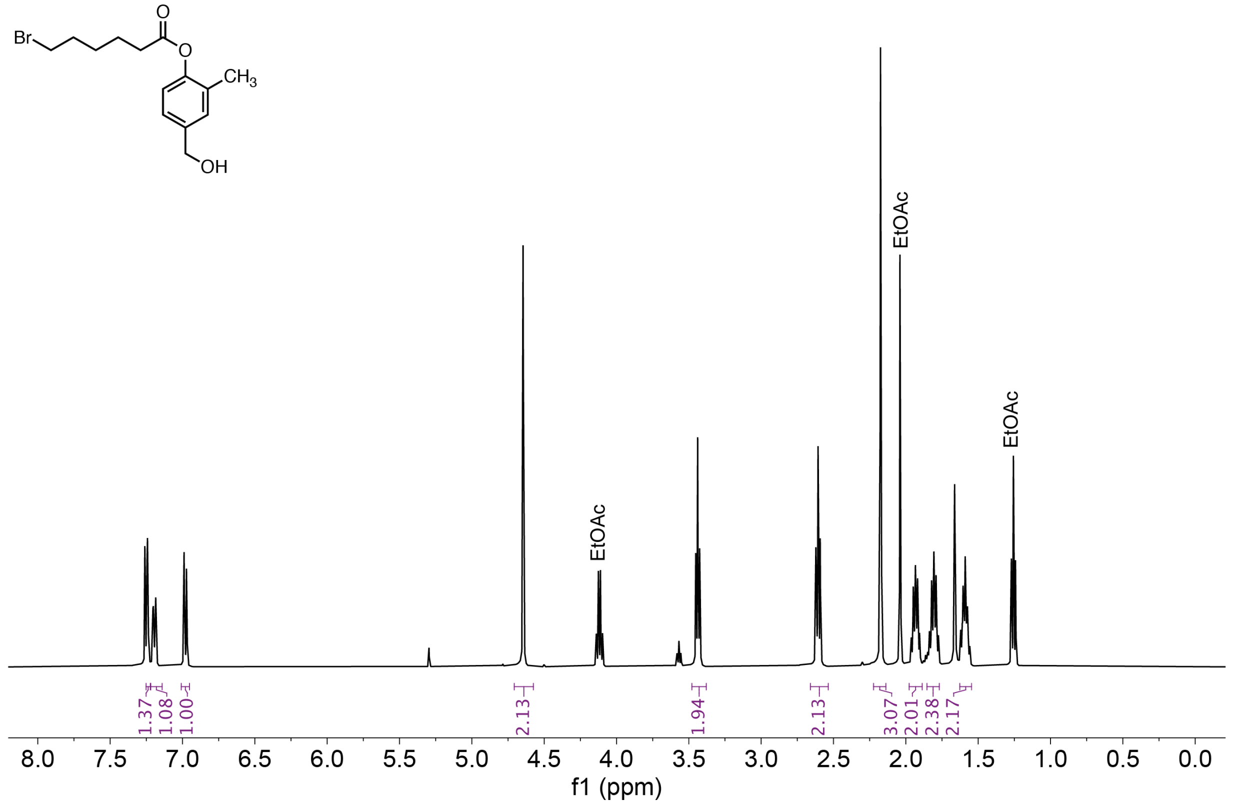

Fig. S3C-I. ^{1}H NMR spectrum of **2c** ($CDCl_3$, 500 MHz, 25 °C).

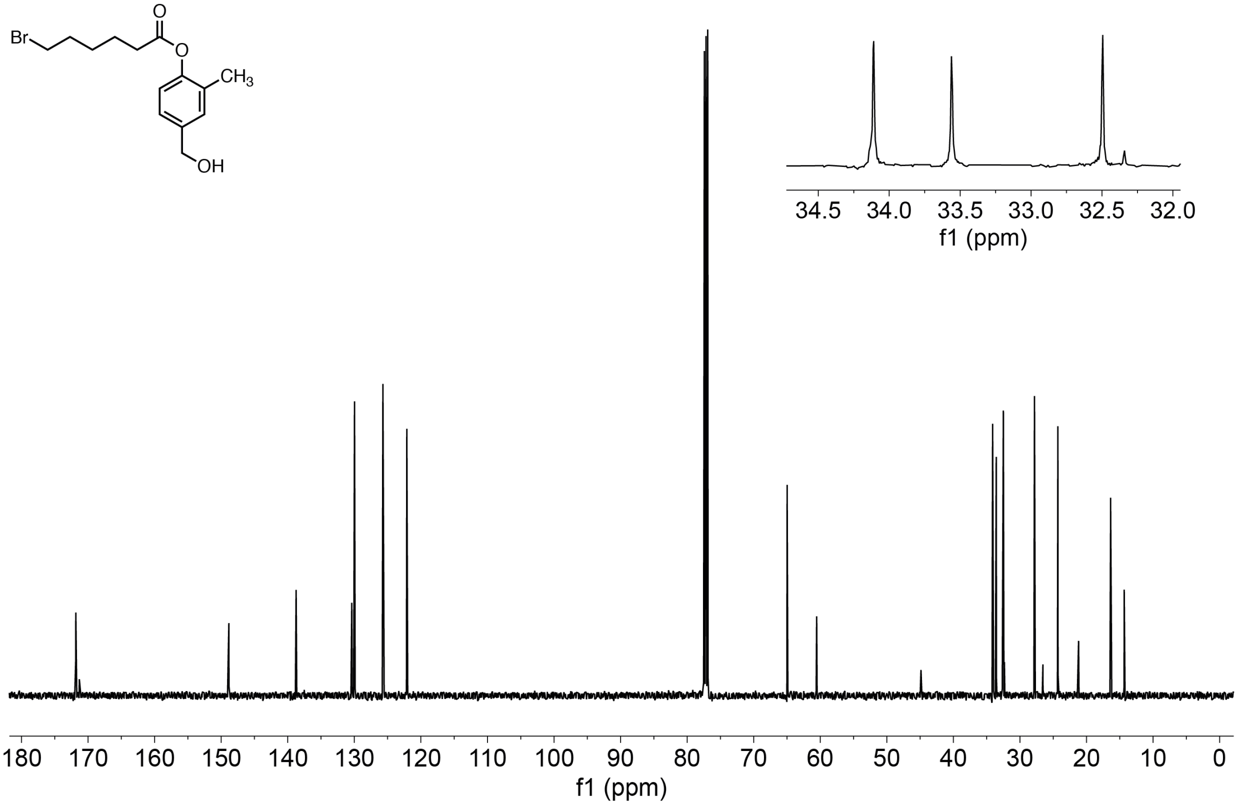

Fig. S3C-II. $^{13}C\{^{1}H\}$ NMR spectrum of **2c** ($CDCl_3$, 126 MHz, 25 °C).

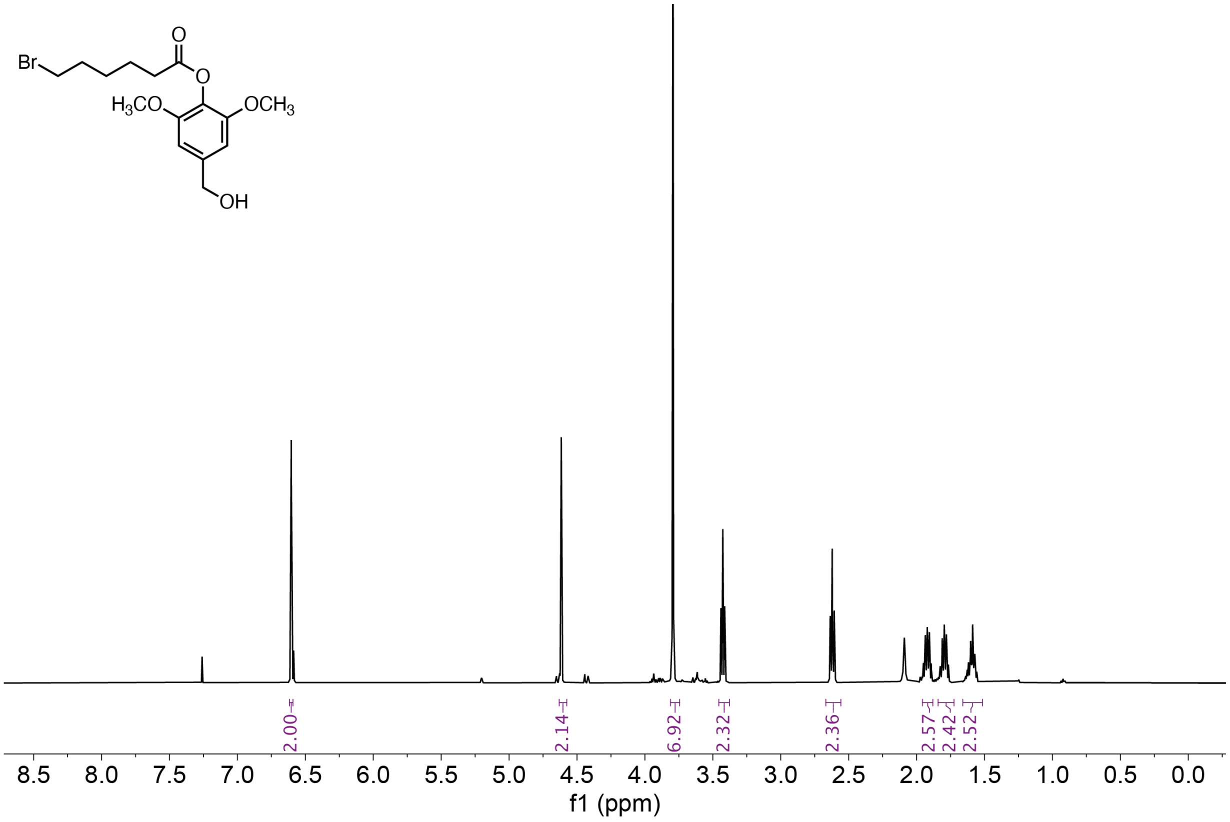

Fig. S3D-I. ^{1}H NMR spectrum of **2d** ($CDCl_3$, 500 MHz, 25 °C).

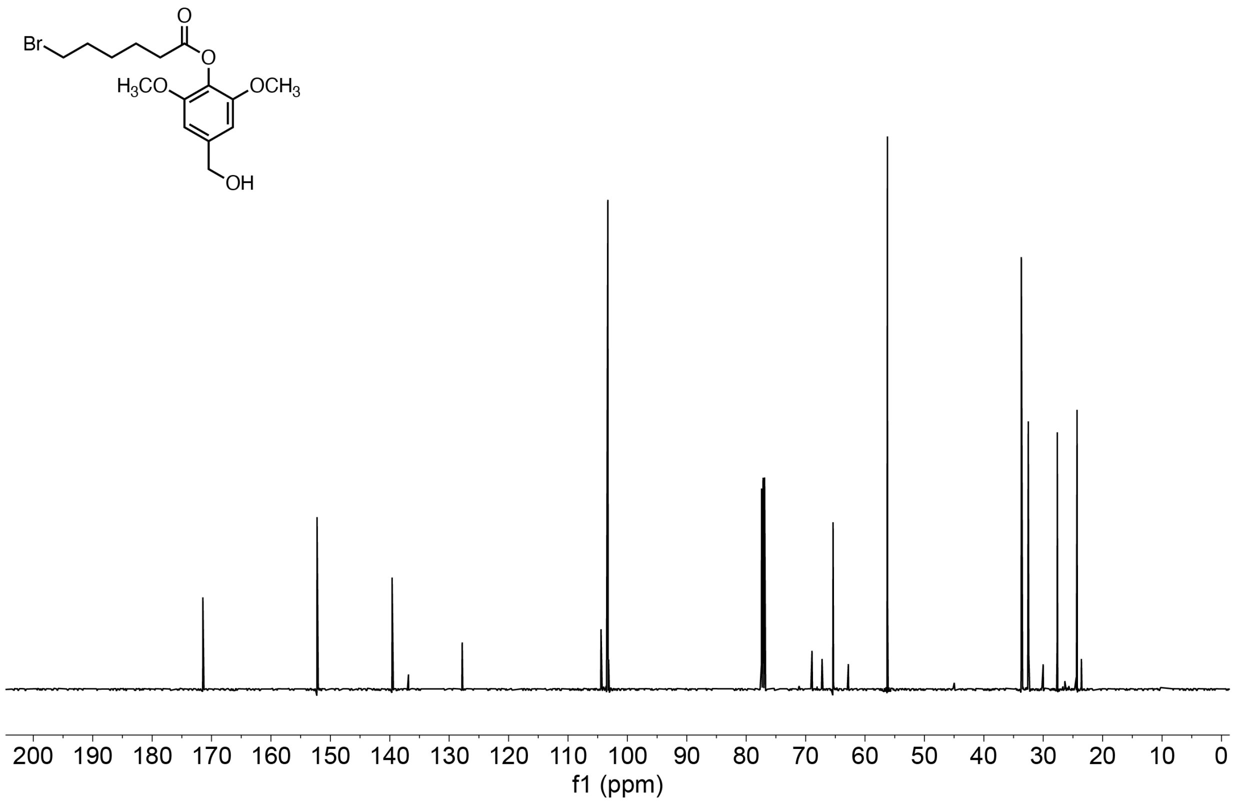

Fig. S3D-II. ^{13}C{^{1}H} NMR spectrum of **2d** ($CDCl_3$, 126 MHz, 25 °C).

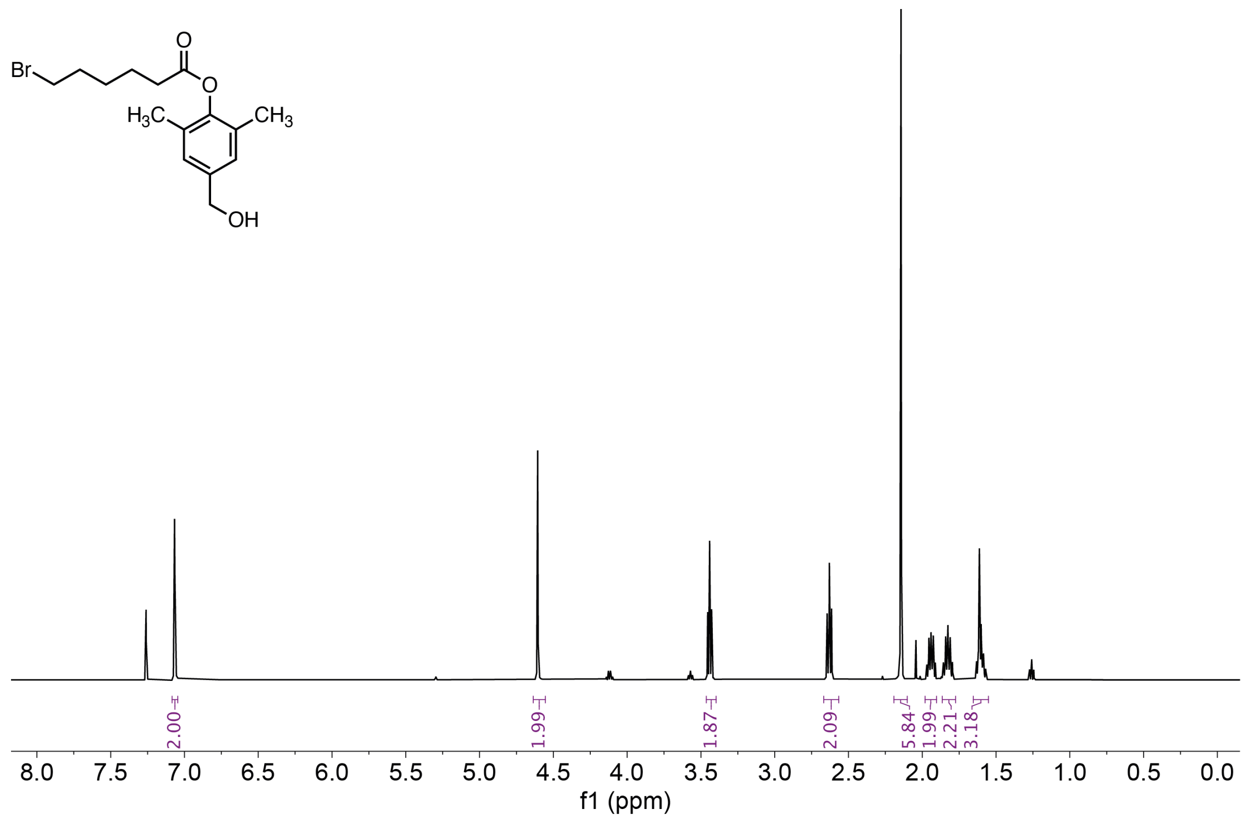

Fig. S3E-I. ^{1}H NMR spectrum of **2e** ($CDCl_3$, 500 MHz, 25 °C).

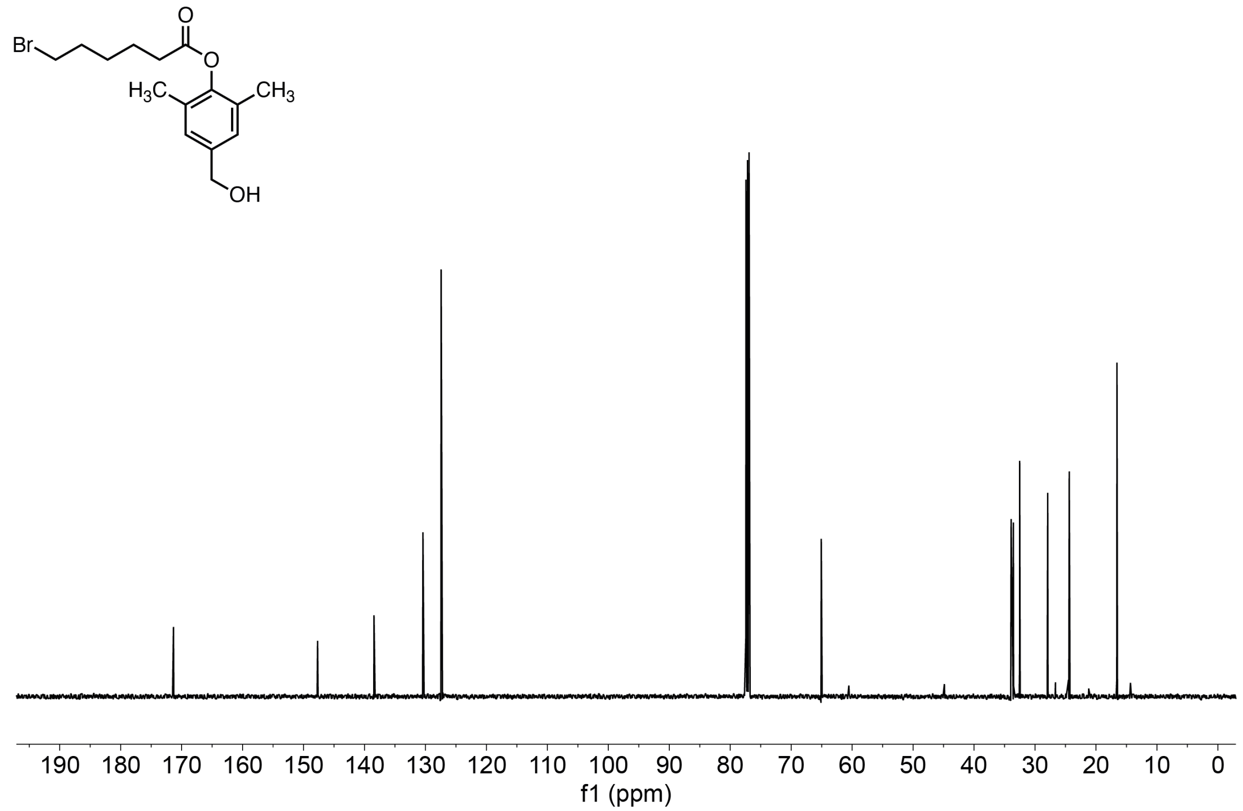

Fig. S3E-II. $^{13}C\{^{1}H\}$ NMR spectrum of **2e** ($CDCl_3$, 126 MHz, 25 °C).

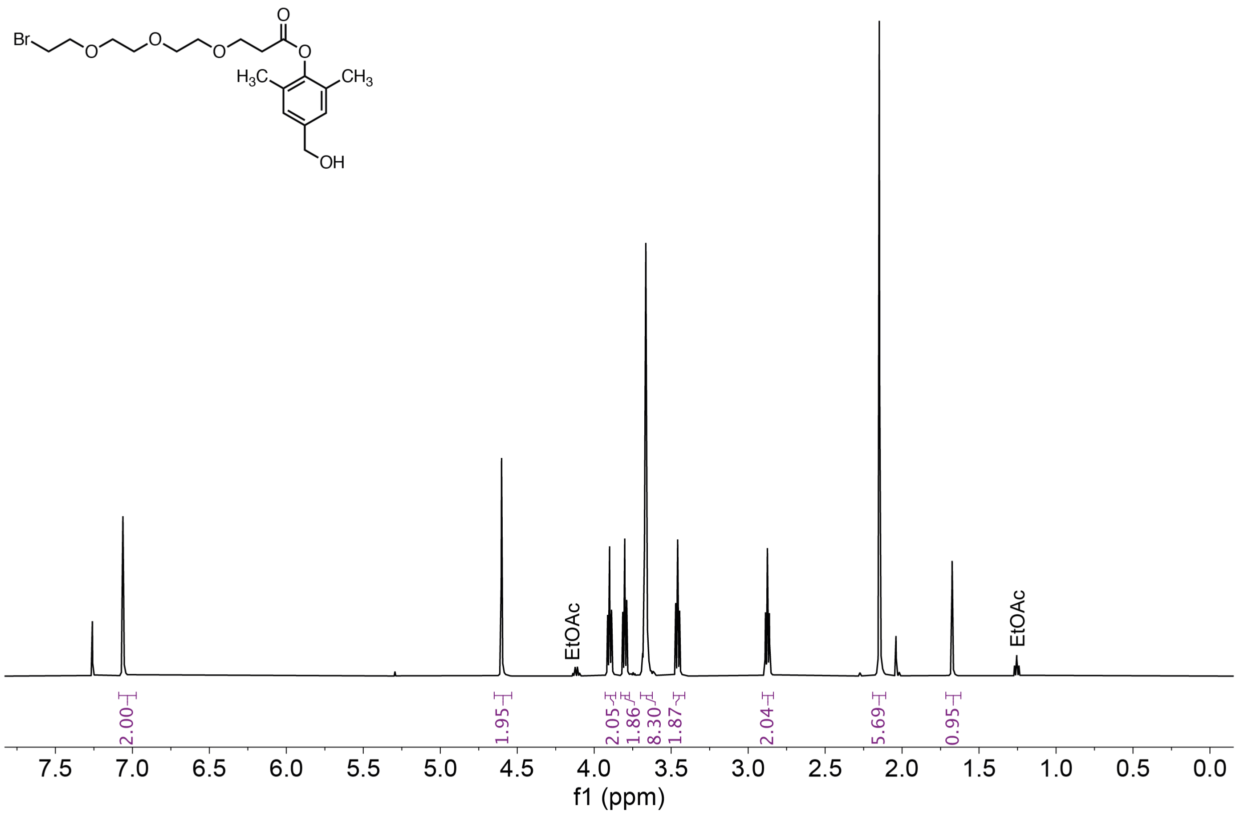

Fig. S3F-I. ^{1}H NMR spectrum of **2f** ($CDCl_3$, 500 MHz, 25 °C).

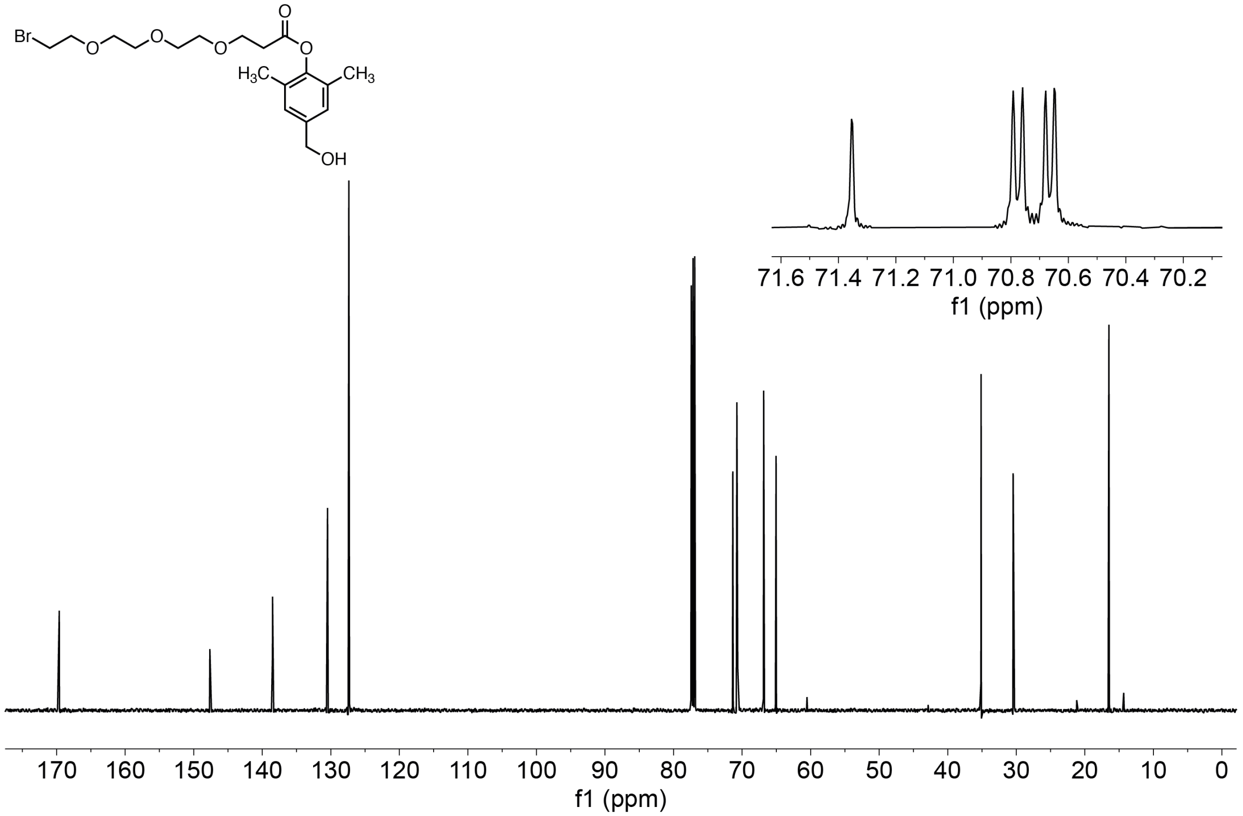

Fig. S3F-II. $^{13}C\{^{1}H\}$ NMR spectrum of **2f** ($CDCl_3$, 126 MHz, 25 °C).

Azide

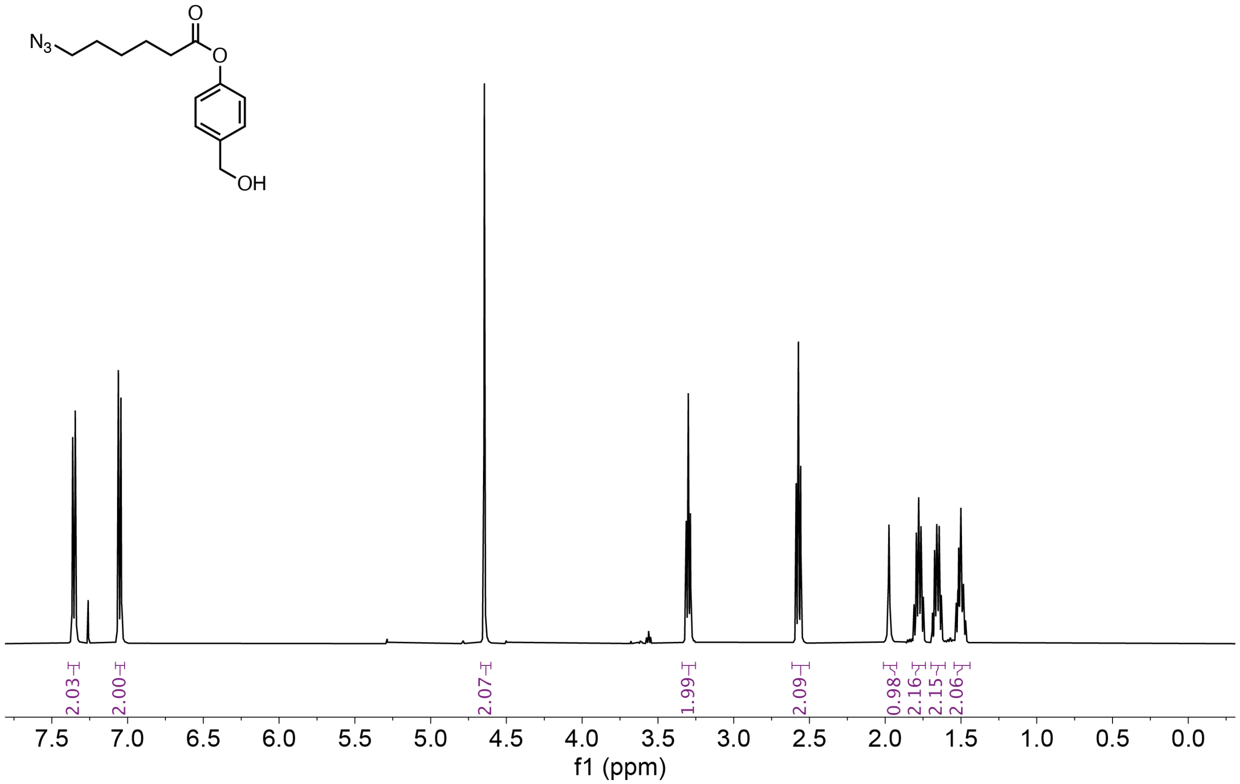

Fig. S4A-I. ^{1}H NMR spectrum of **3a** ($CDCl_3$, 500 MHz, 25 °C).

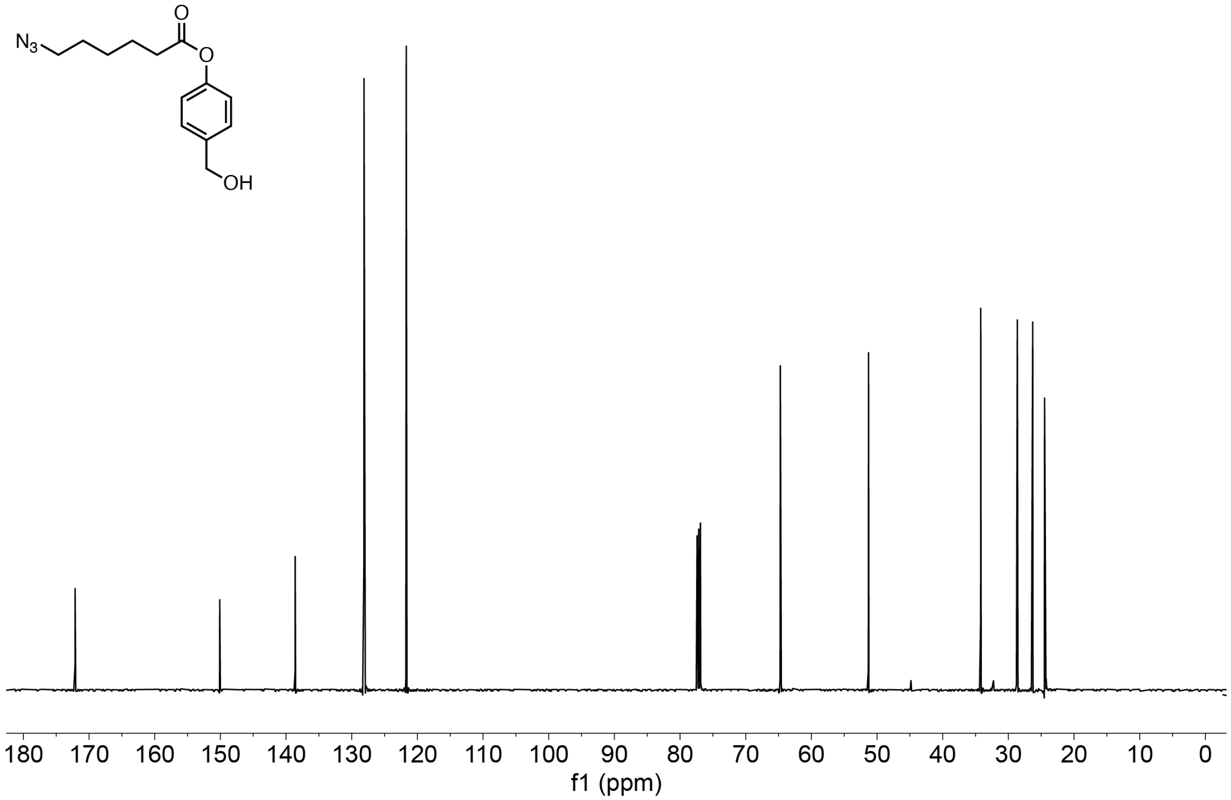

Fig. S4A-II. $^{13}C\{^{1}H\}$ NMR spectrum of **3a** ($CDCl_3$, 126 MHz, 25 °C).

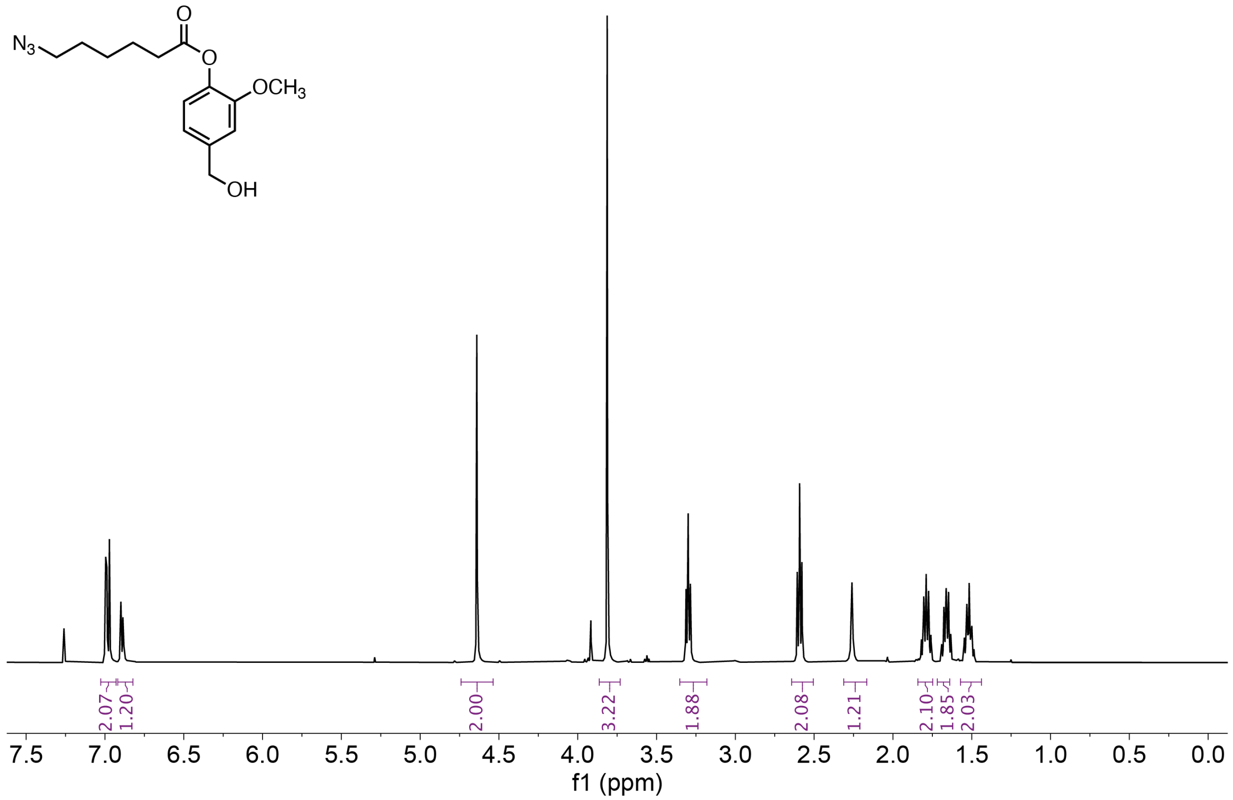

Fig. S4B-I. ^{1}H NMR spectrum of **3b** ($CDCl_3$, 500 MHz, 25 °C).

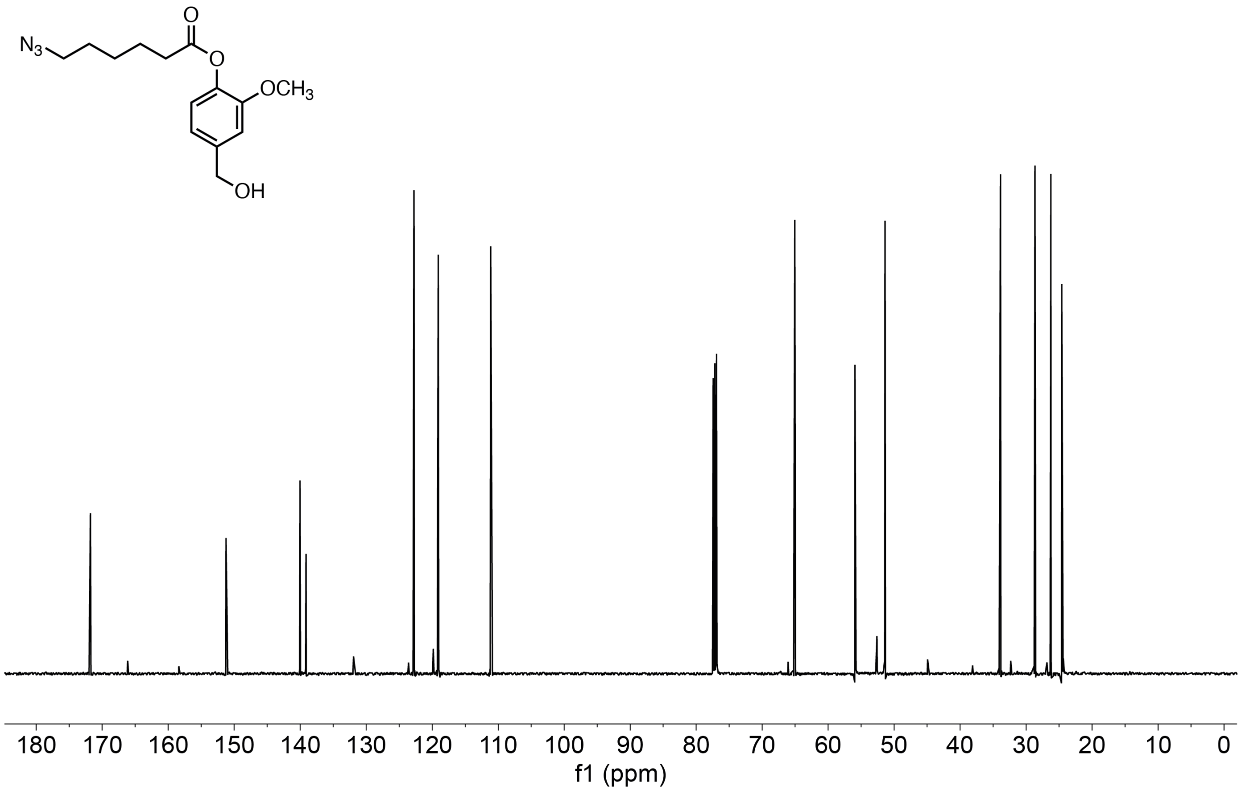

Fig. S4B-II. $^{13}C\{^{1}H\}$ NMR spectrum of **3b** ($CDCl_3$, 126 MHz, 25 °C).

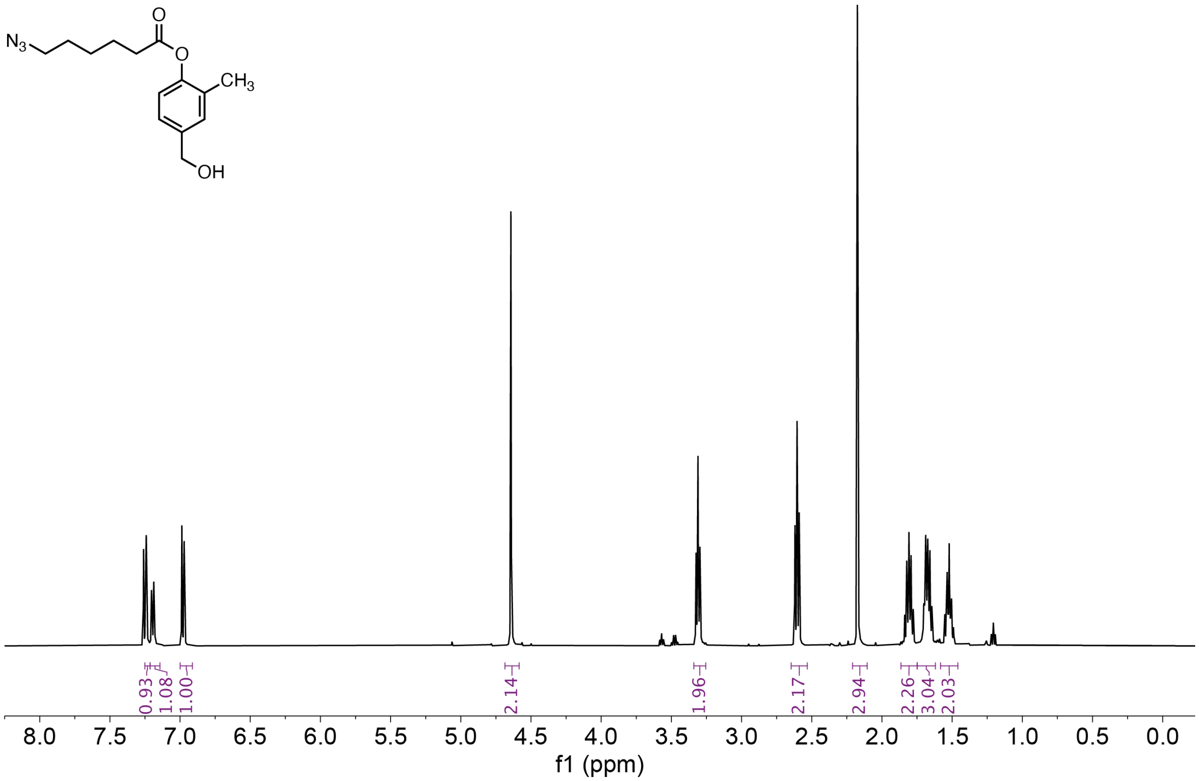

Fig. S4C-I. ^{1}H NMR spectrum of **3c** ($CDCl_3$, 500 MHz, 25 °C).

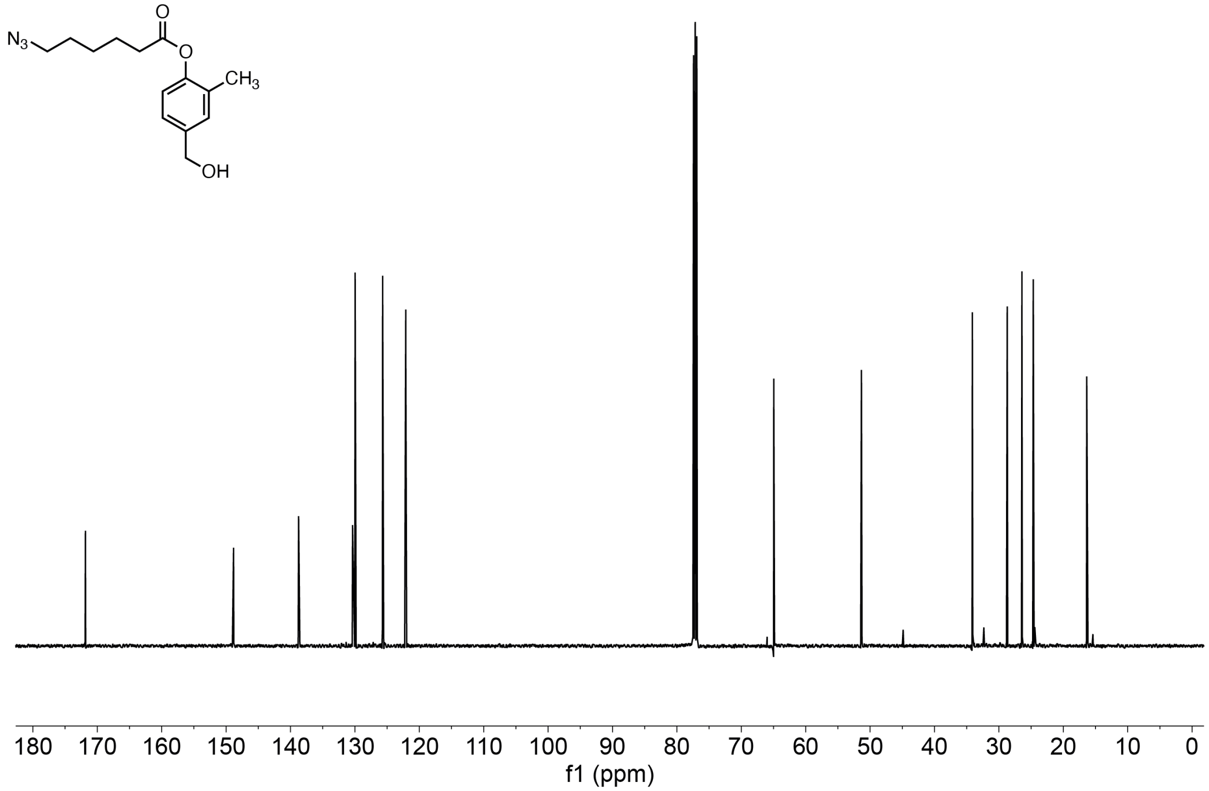

Fig. S4C-II. $^{13}C\{^{1}H\}$ NMR spectrum of **3c** ($CDCl_3$, 126 MHz, 25 °C).

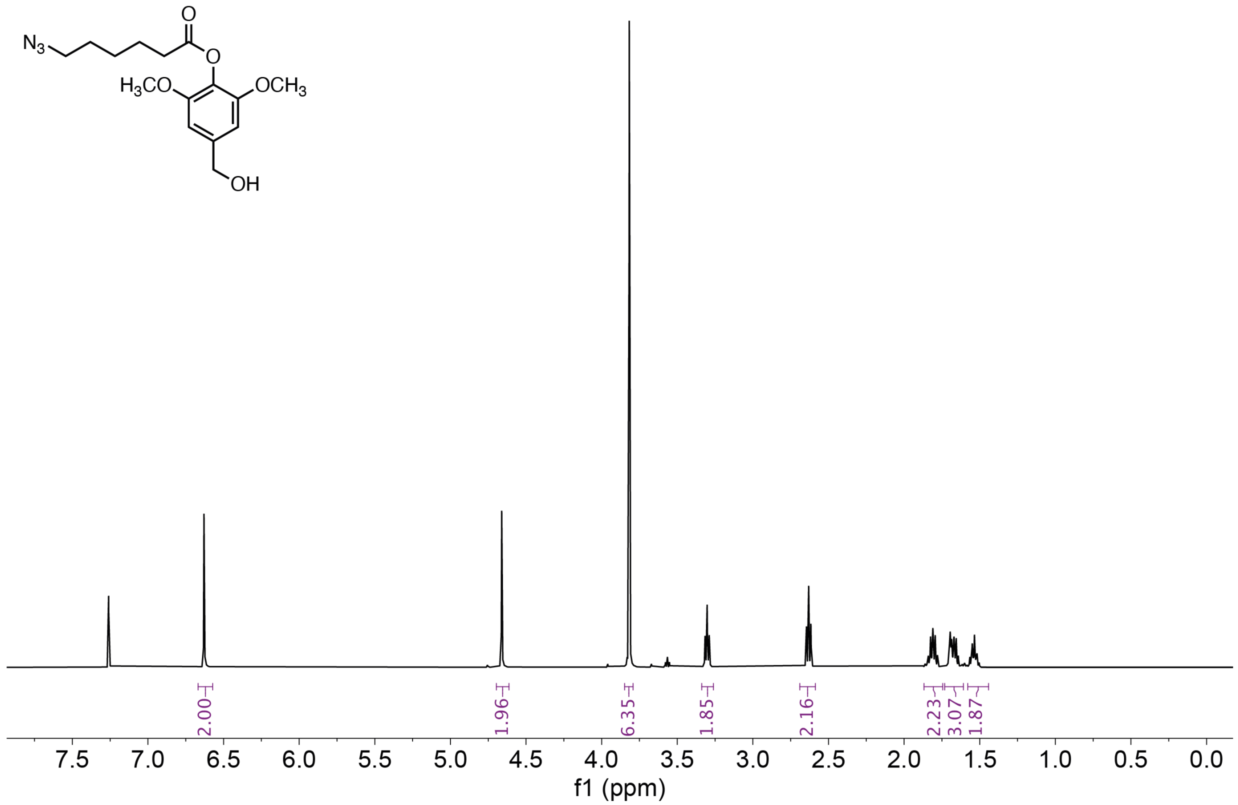

Fig. S4D-I. ^{1}H NMR spectrum of **3d** ($CDCl_3$, 500 MHz, 25 °C).

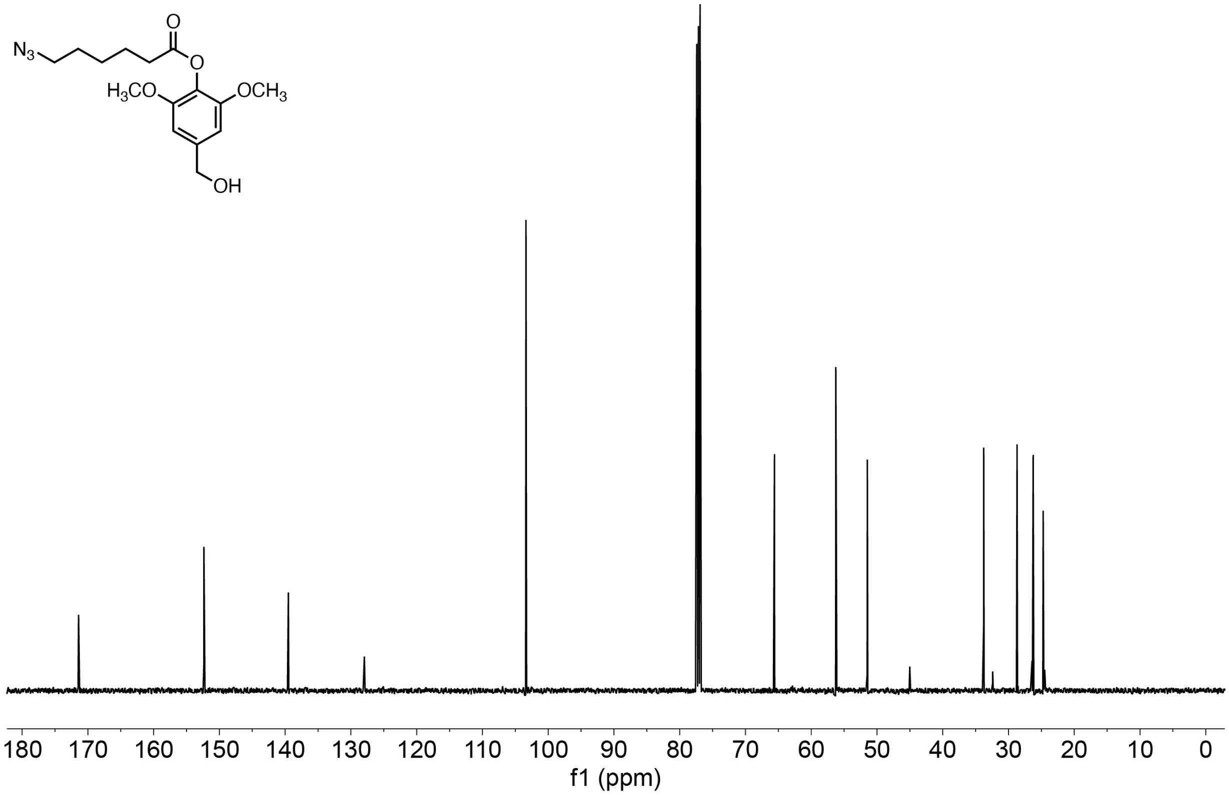

Fig. S4D-II. $^{13}C\{^{1}H\}$ NMR spectrum of **3d** ($CDCl_3$, 126 MHz, 25 °C).

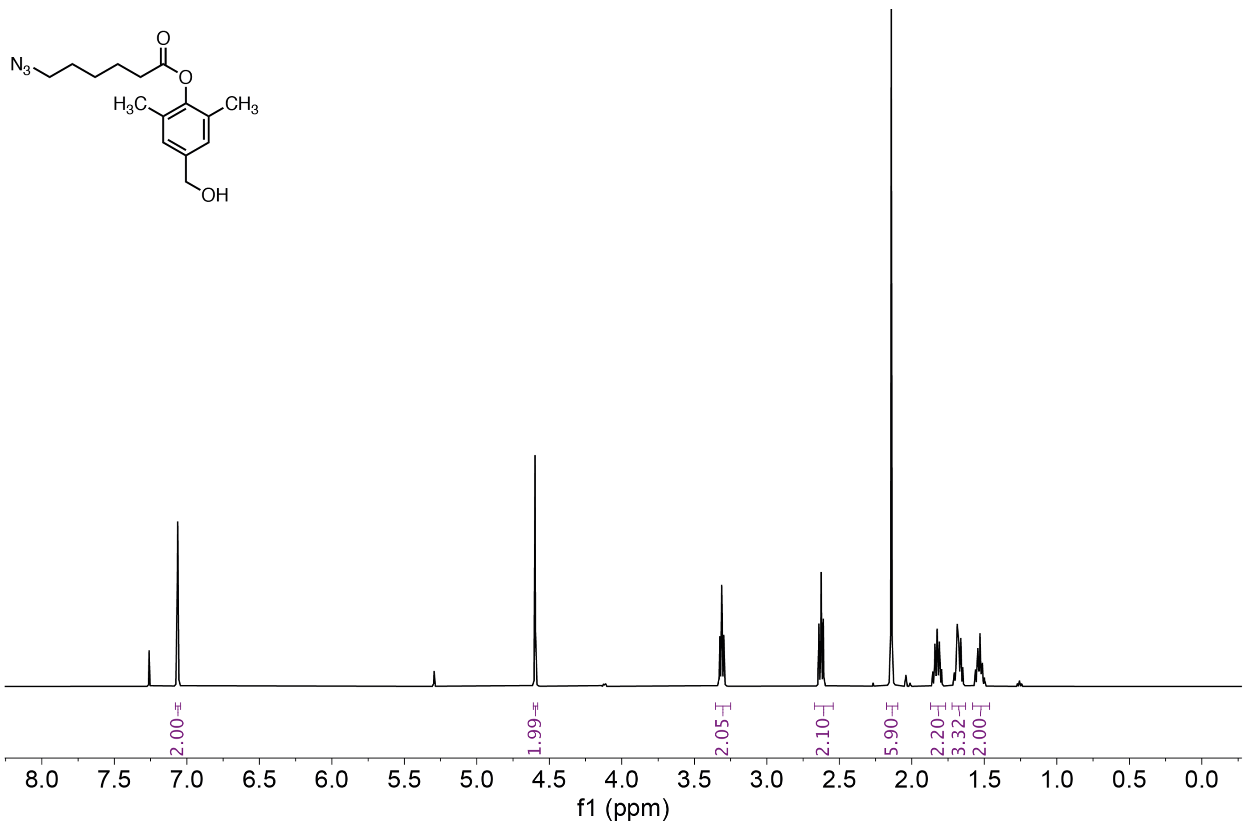

Fig. S4E-I. ^{1}H NMR spectrum of **3e** ($CDCl_3$, 500 MHz, 25 °C).

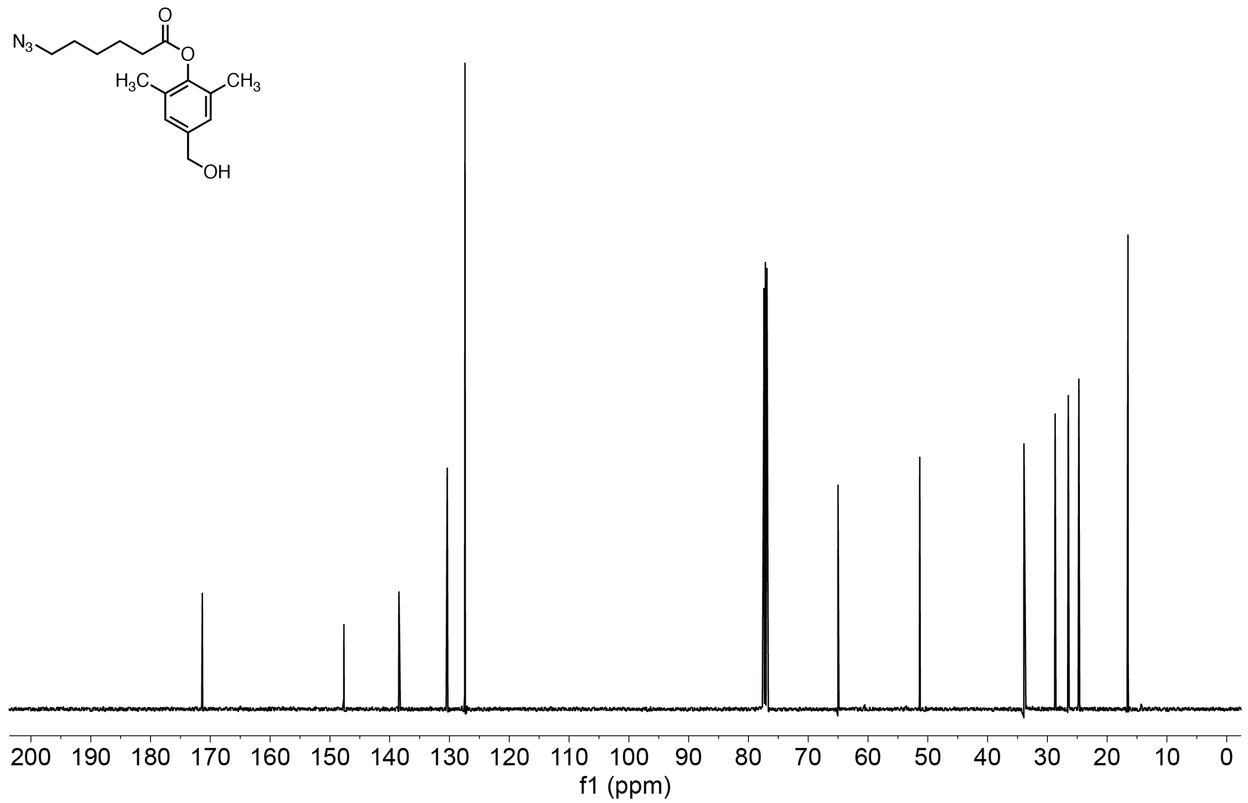

Fig. S4E-II. $^{13}C\{^{1}H\}$ NMR spectrum of **3e** ($CDCl_3$, 126 MHz, 25 °C).

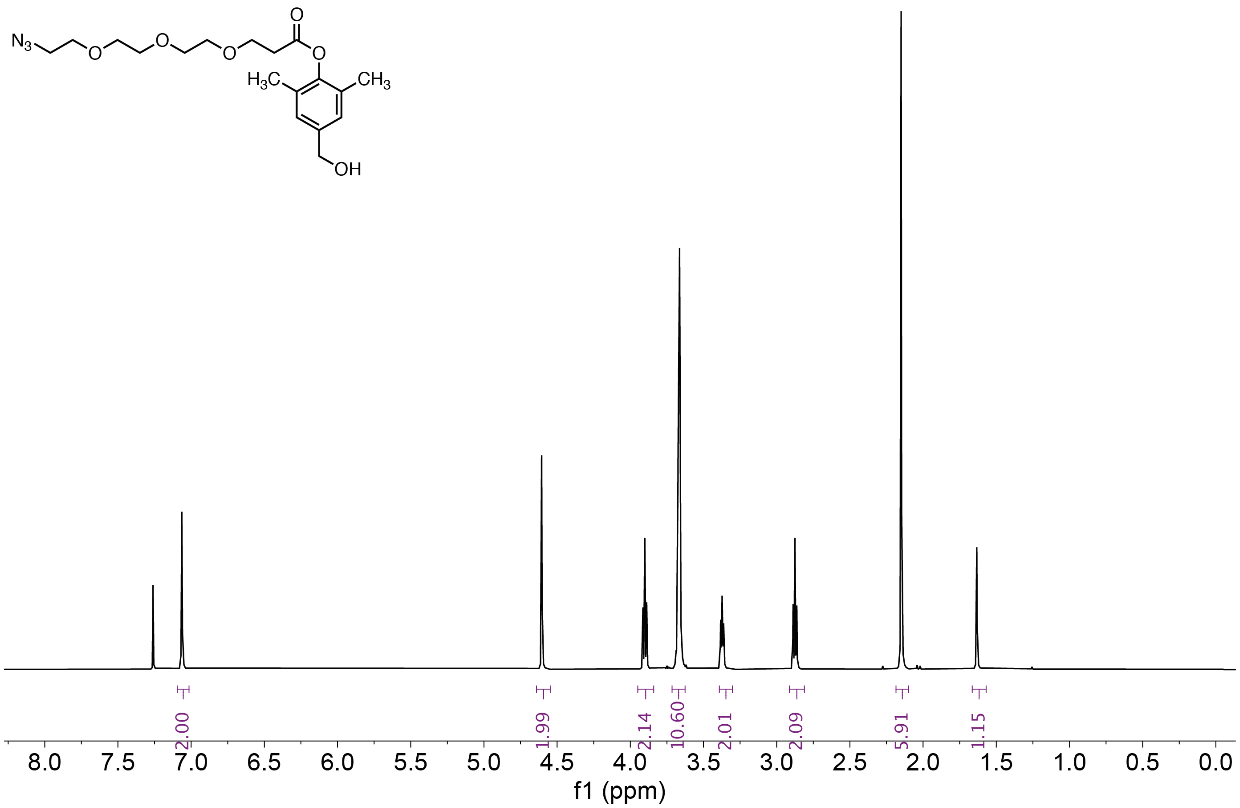

Fig. S4F-I. ^{1}H NMR spectrum of **3f** ($CDCl_3$, 500 MHz, 25 °C).

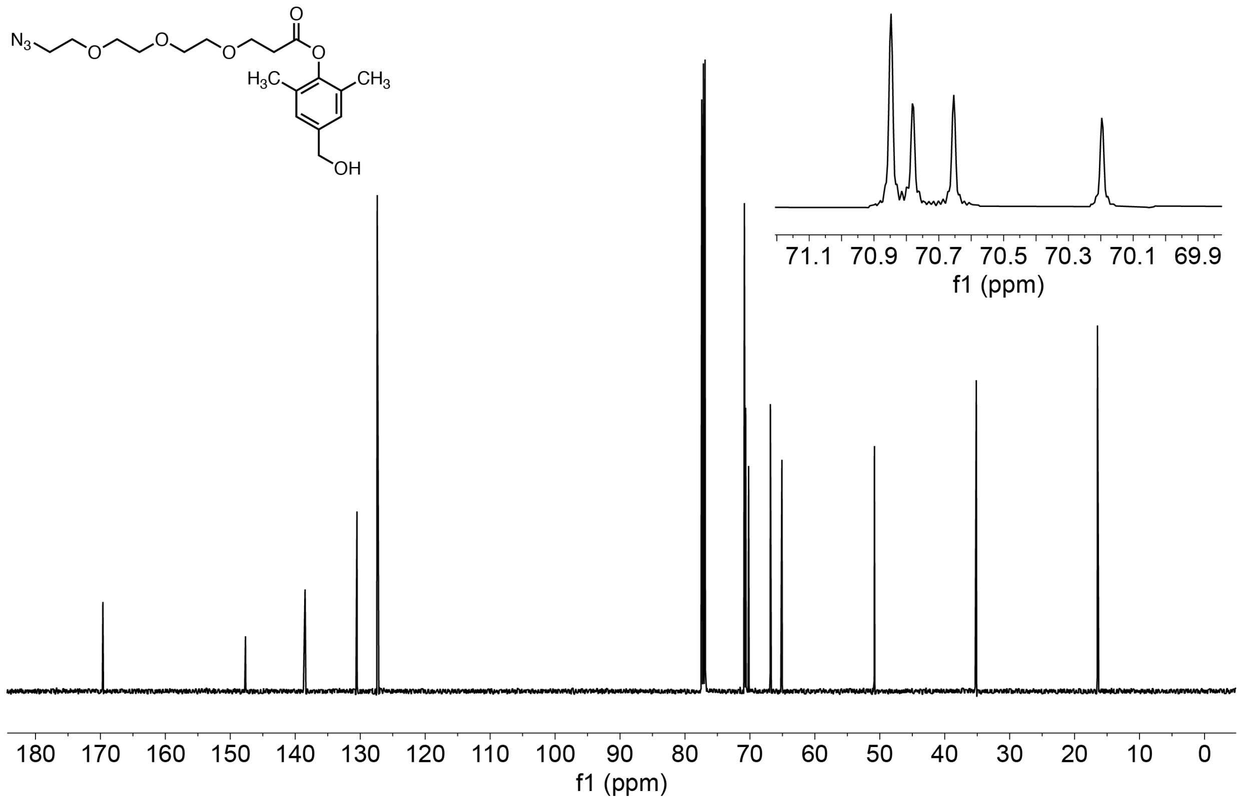

Fig. S4F-II. $^{13}C\{^{1}H\}$ NMR spectrum of **3f** ($CDCl_3$, 126 MHz, 25 °C).

Carbonyl

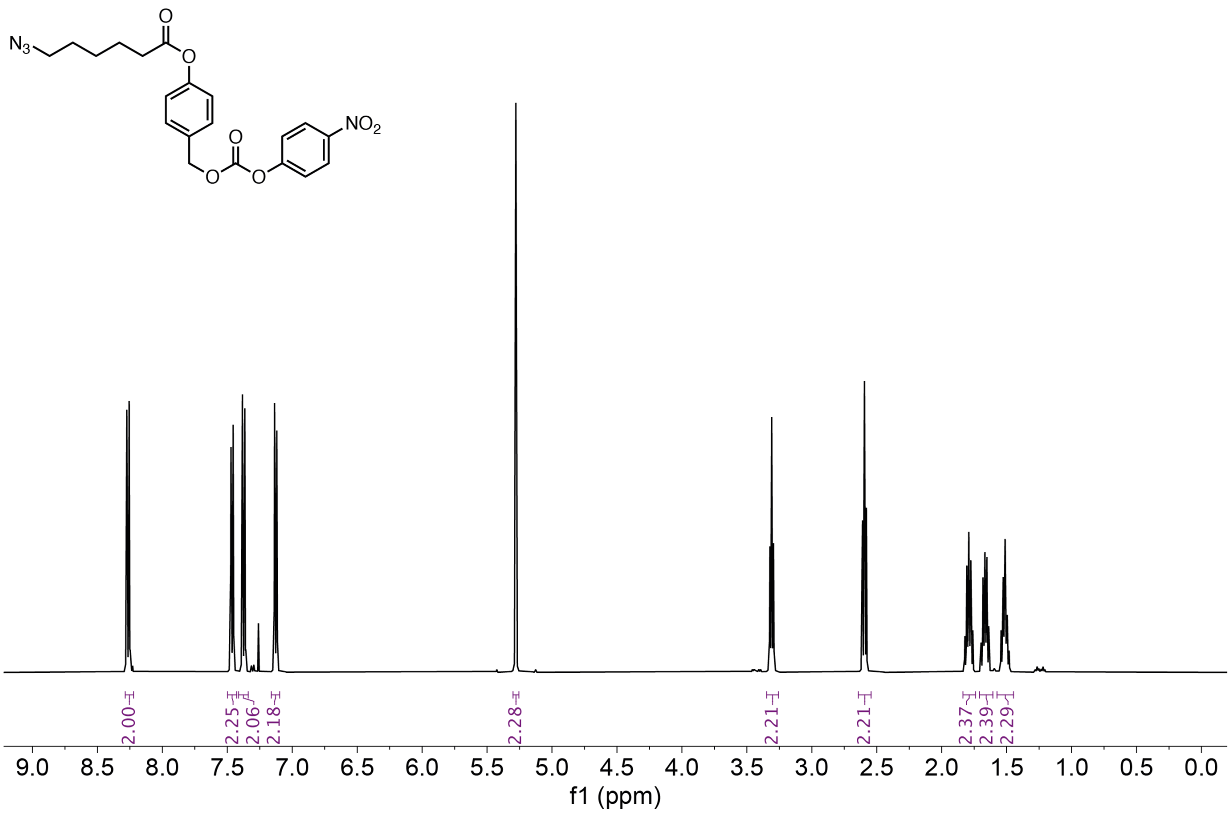

Fig. S5A-I. ^1H NMR spectrum of **4a** ($CDCl_3$, 500 MHz, 25 °C).

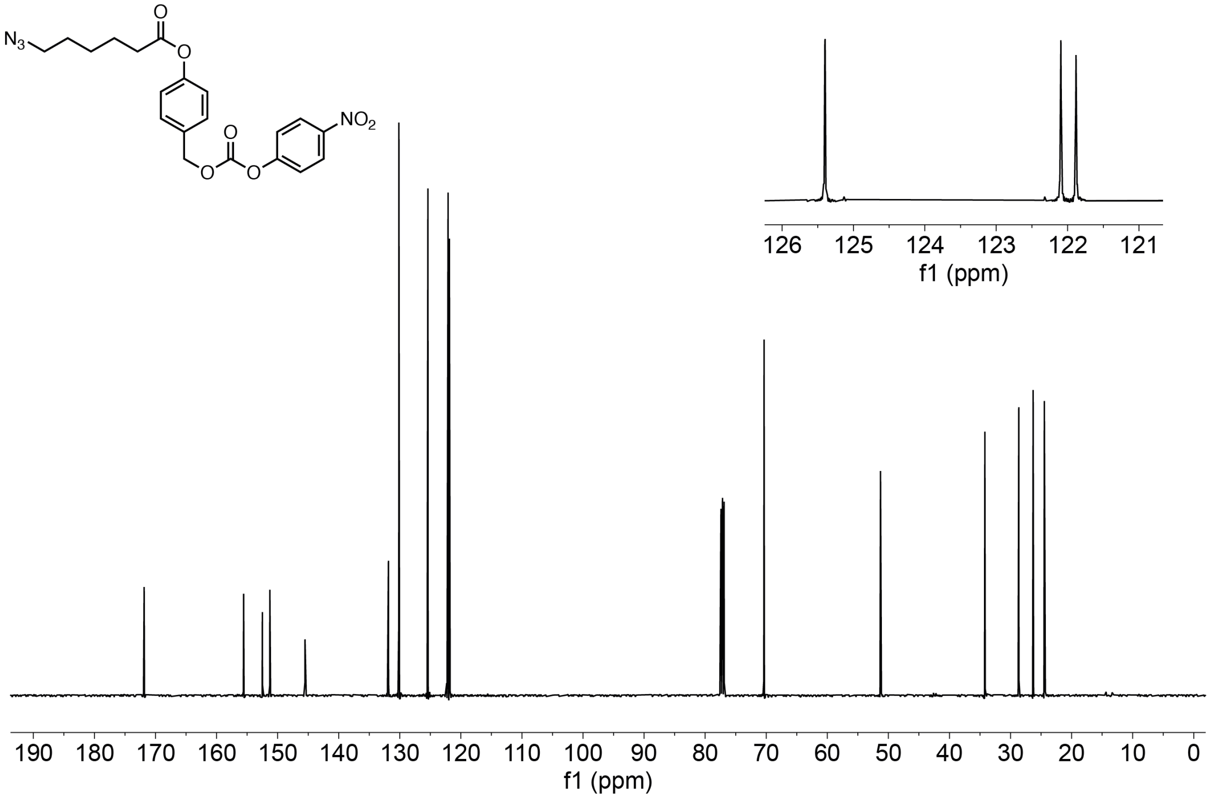

Fig. S5A-II. $^{13}C\{^1H\}$ NMR spectrum of **4a** ($CDCl_3$, 126 MHz, 25 °C).

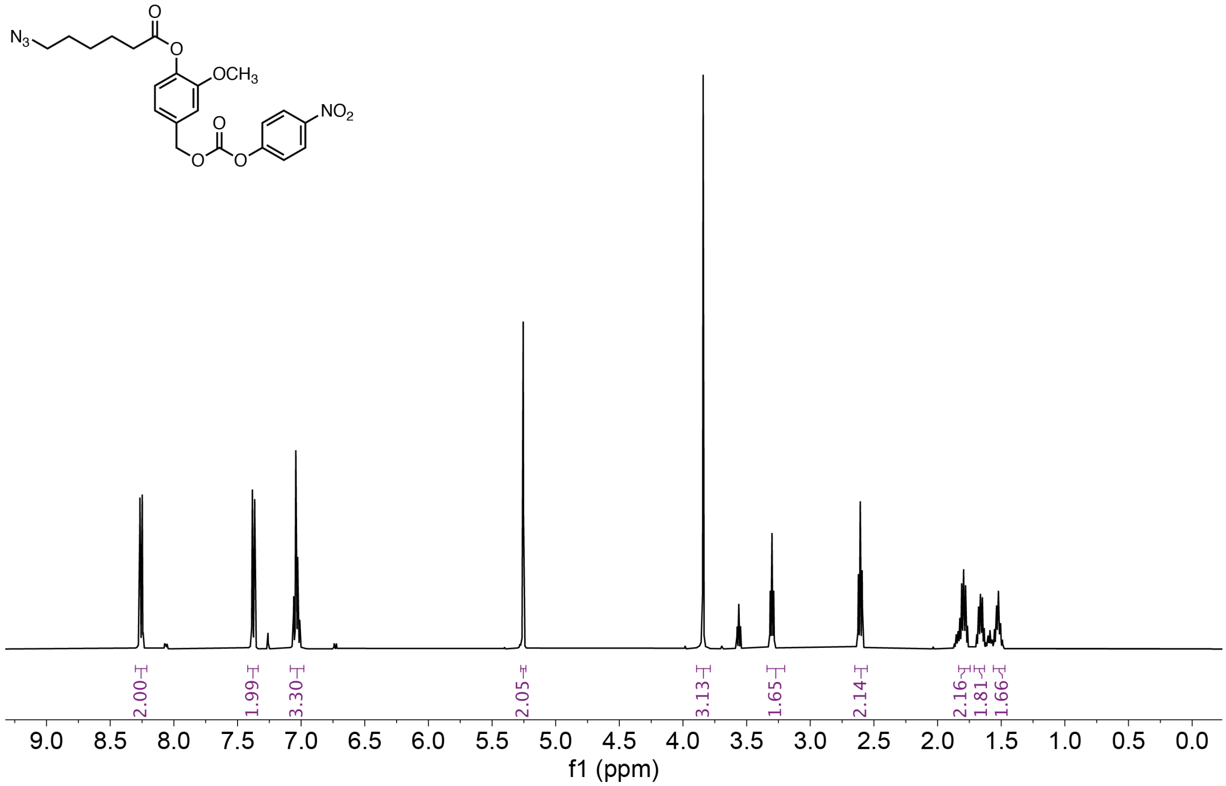

Fig. S5B-I. ^{1}H NMR spectrum of **4b** ($CDCl_3$, 500 MHz, 25 °C).

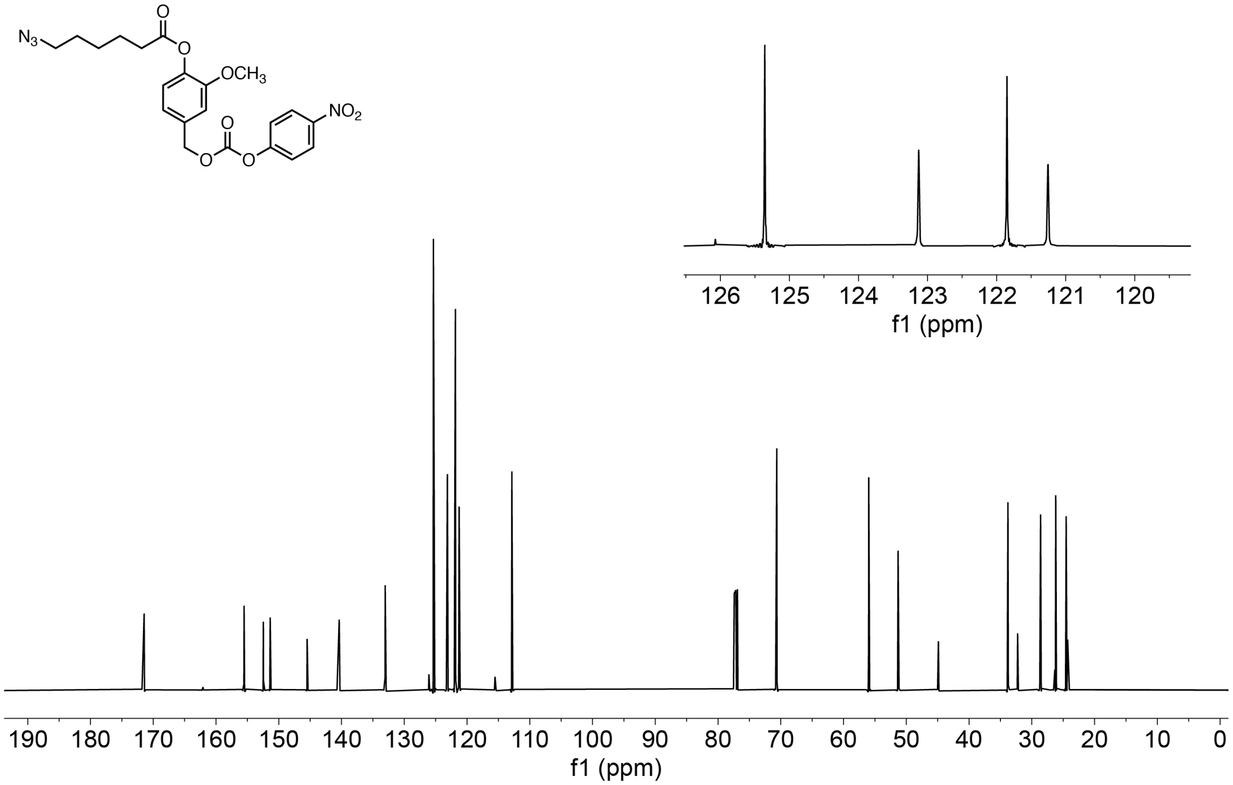

Fig. S5B-II. $^{13}C\{^{1}H\}$ NMR spectrum of **4b** ($CDCl_3$, 126 MHz, 25 °C).

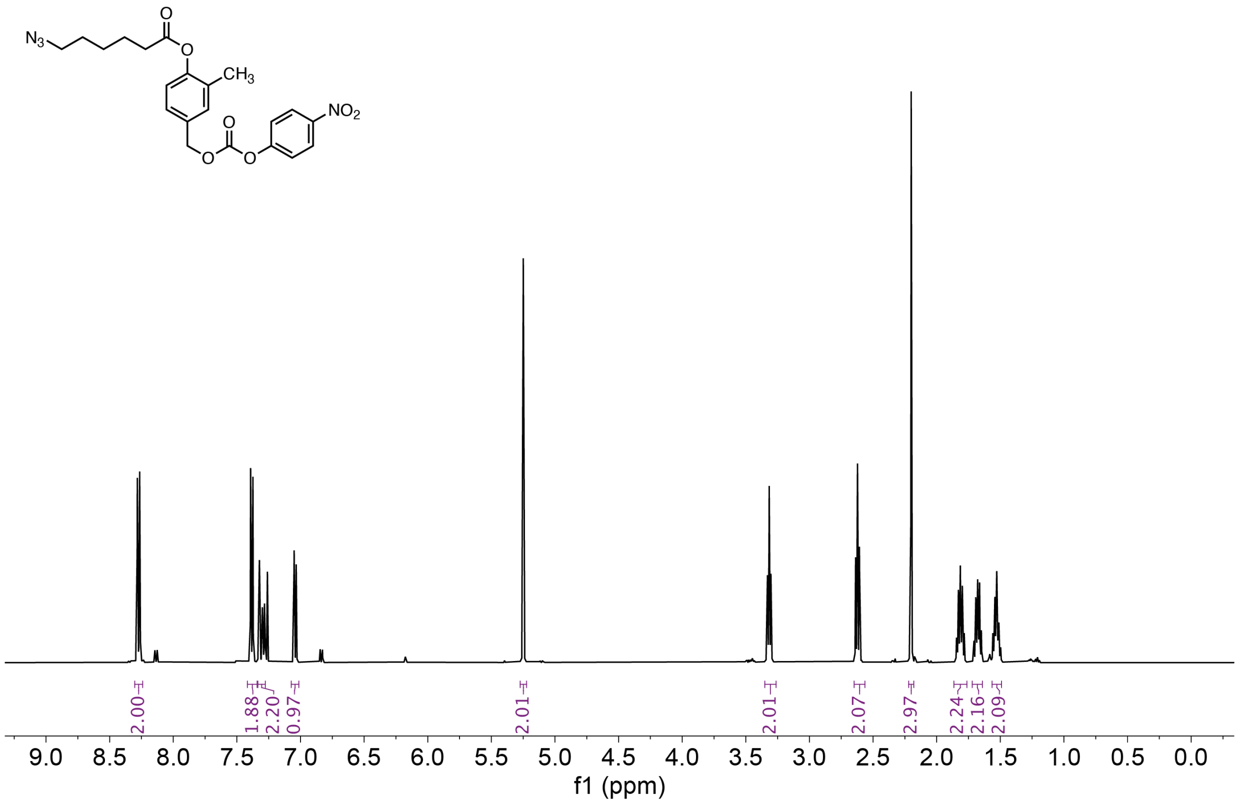

Fig. S5C-I. ^{1}H NMR spectrum of **4c** ($CDCl_3$, 500 MHz, 25 °C).

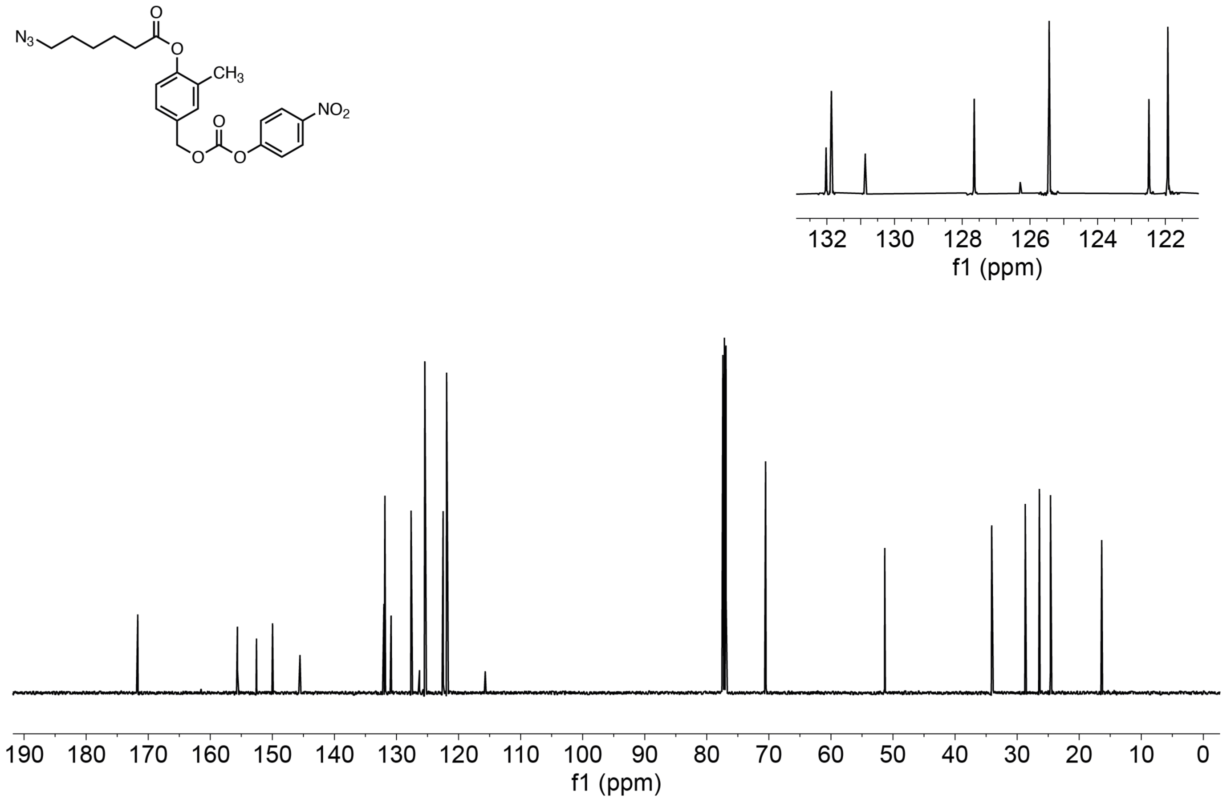

Fig. S5C-II. $^{13}C\{^{1}H\}$ NMR spectrum of **4c** ($CDCl_3$, 126 MHz, 25 °C).

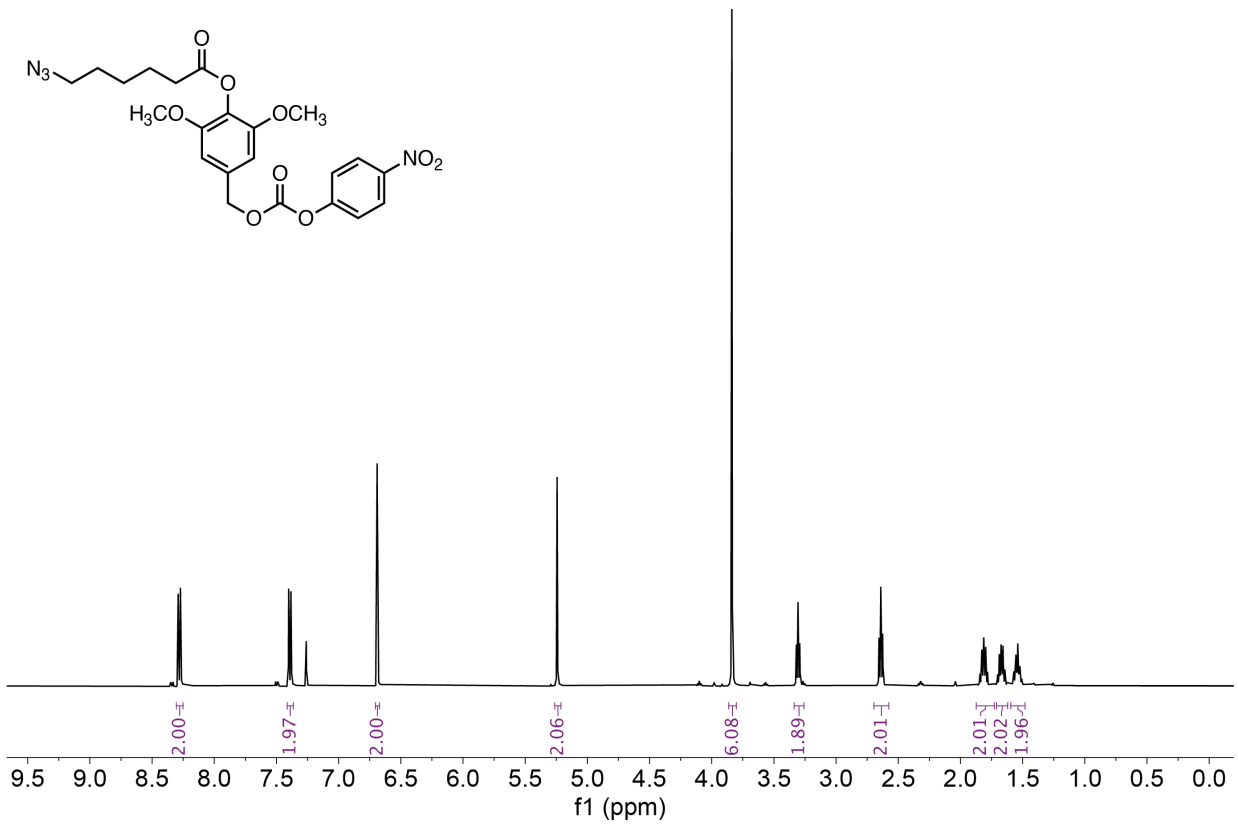

Fig. S5D-I. ^{1}H NMR spectrum of **4d** ($CDCl_3$, 500 MHz, 25 °C).

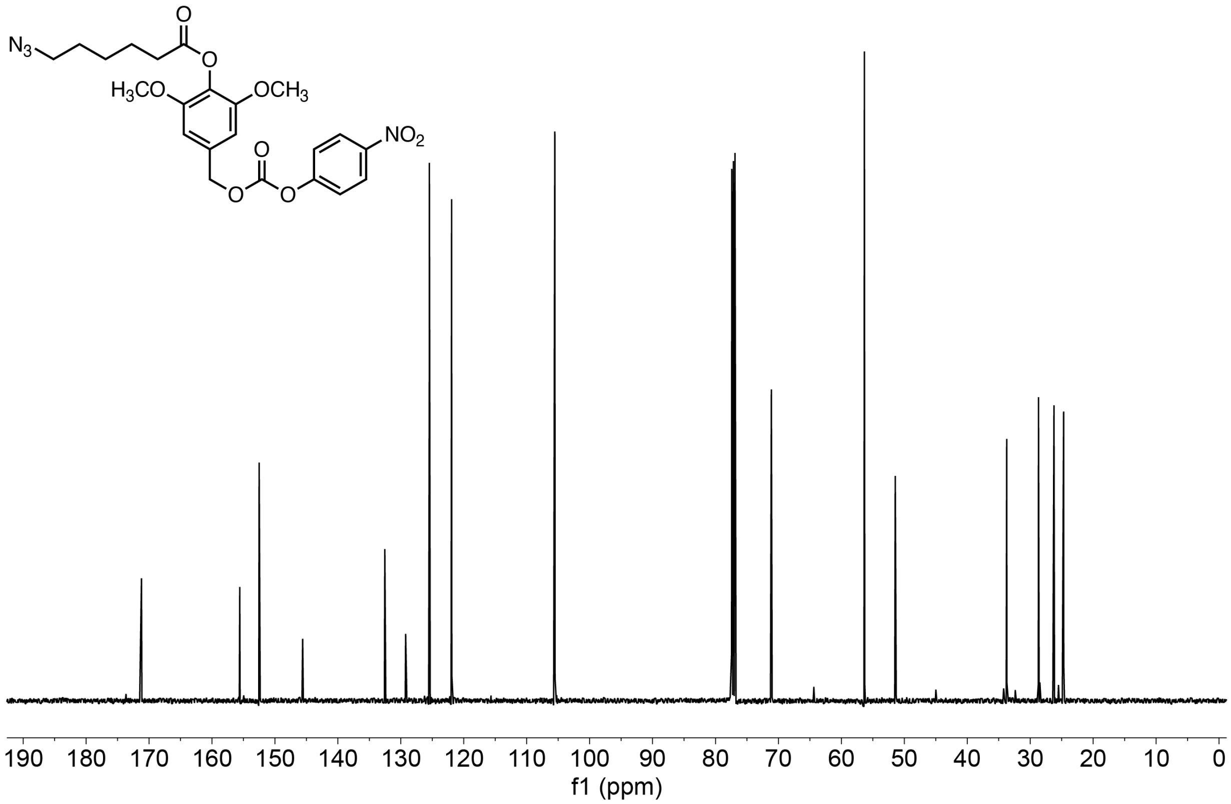

Fig. S5D-II. $^{13}C\{^{1}H\}$ NMR spectrum of **4d** ($CDCl_3$, 126 MHz, 25 °C).

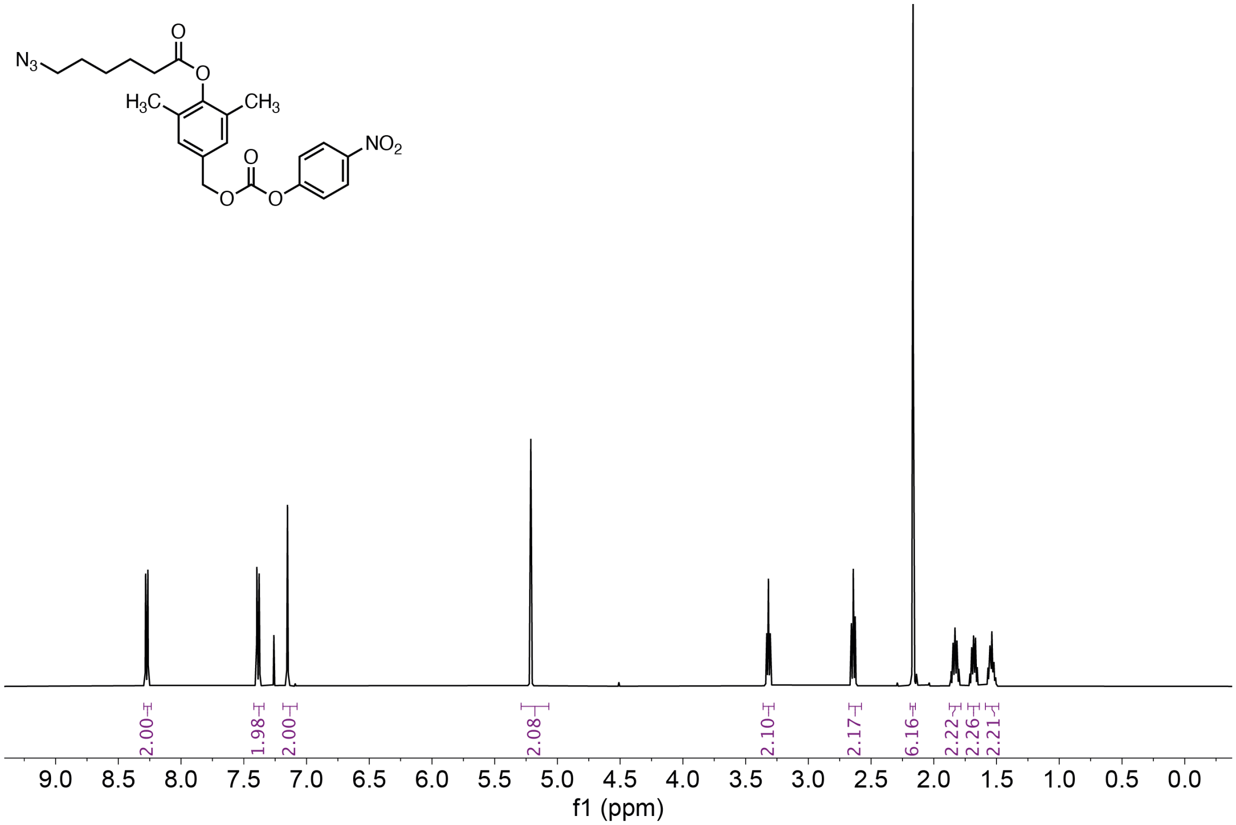

Fig. S5E-I. ^{1}H NMR spectrum of **4e** ($CDCl_3$, 500 MHz, 25 °C).

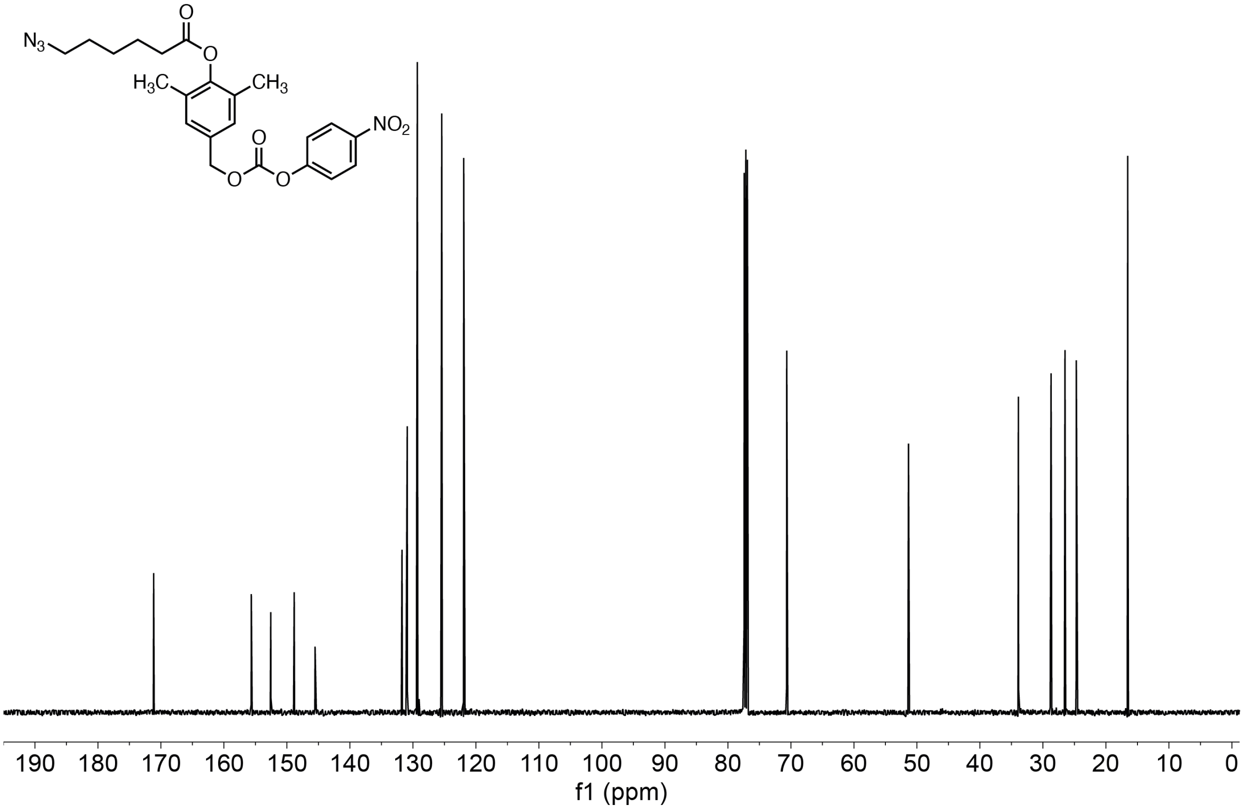

Fig. S5E-II. $^{13}C\{^{1}H\}$ NMR spectrum of **4e** ($CDCl_3$, 126 MHz, 25 °C).

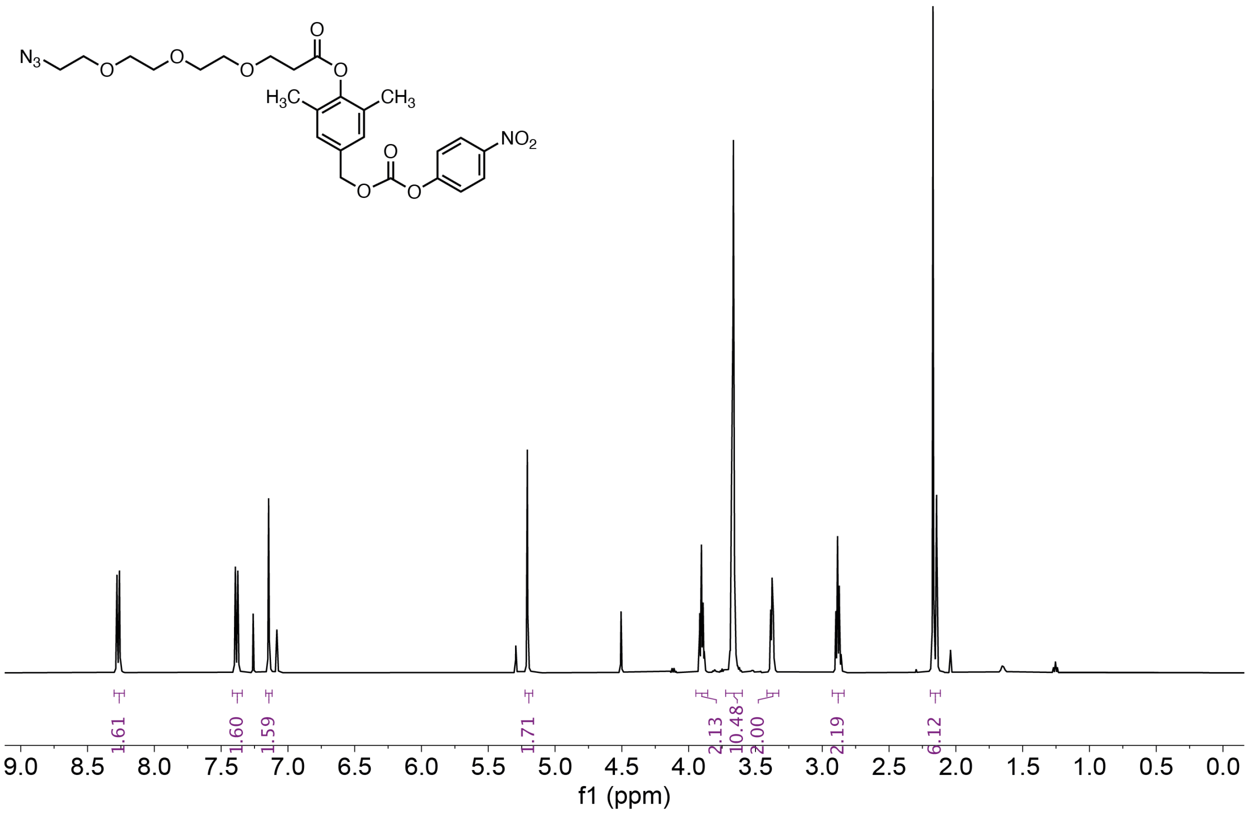

Fig. S5F-I. ^{1}H NMR spectrum of **4f** ($CDCl_3$, 500 MHz, 25 °C).

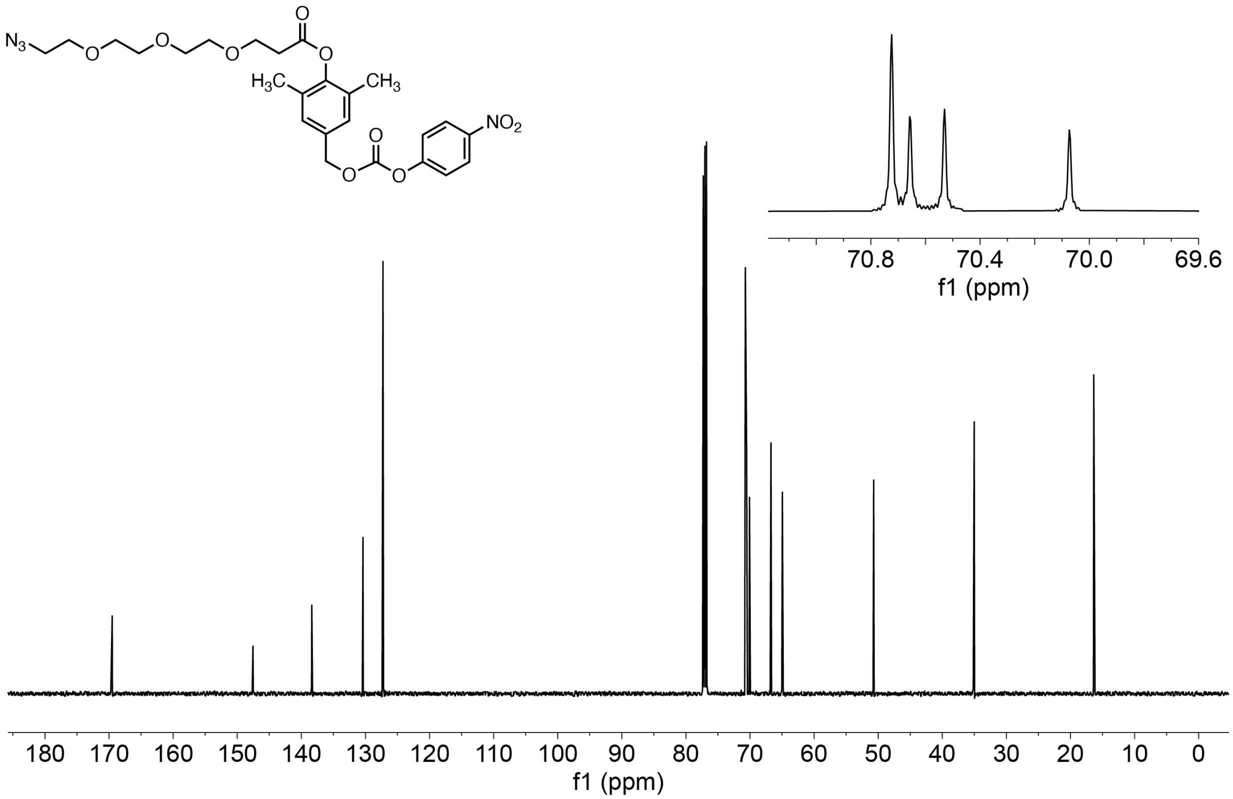

Fig. S5F-II. $^{13}C\{^{1}H\}$ NMR spectrum of **4f** ($CDCl_3$, 126 MHz, 25 °C).

Resiquimod

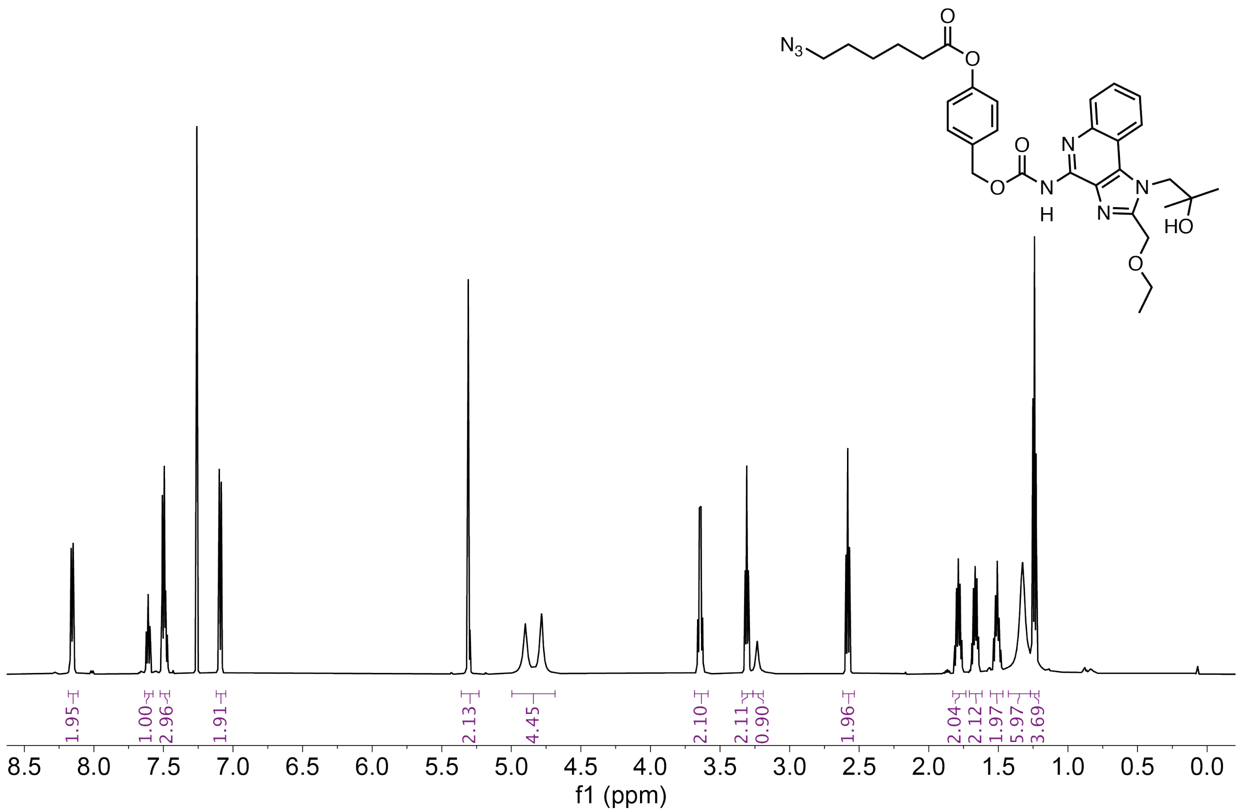

Fig. S6A-I. ^{1}H NMR spectrum of **5a** ($CDCl_3$, 500 MHz, 25 °C).

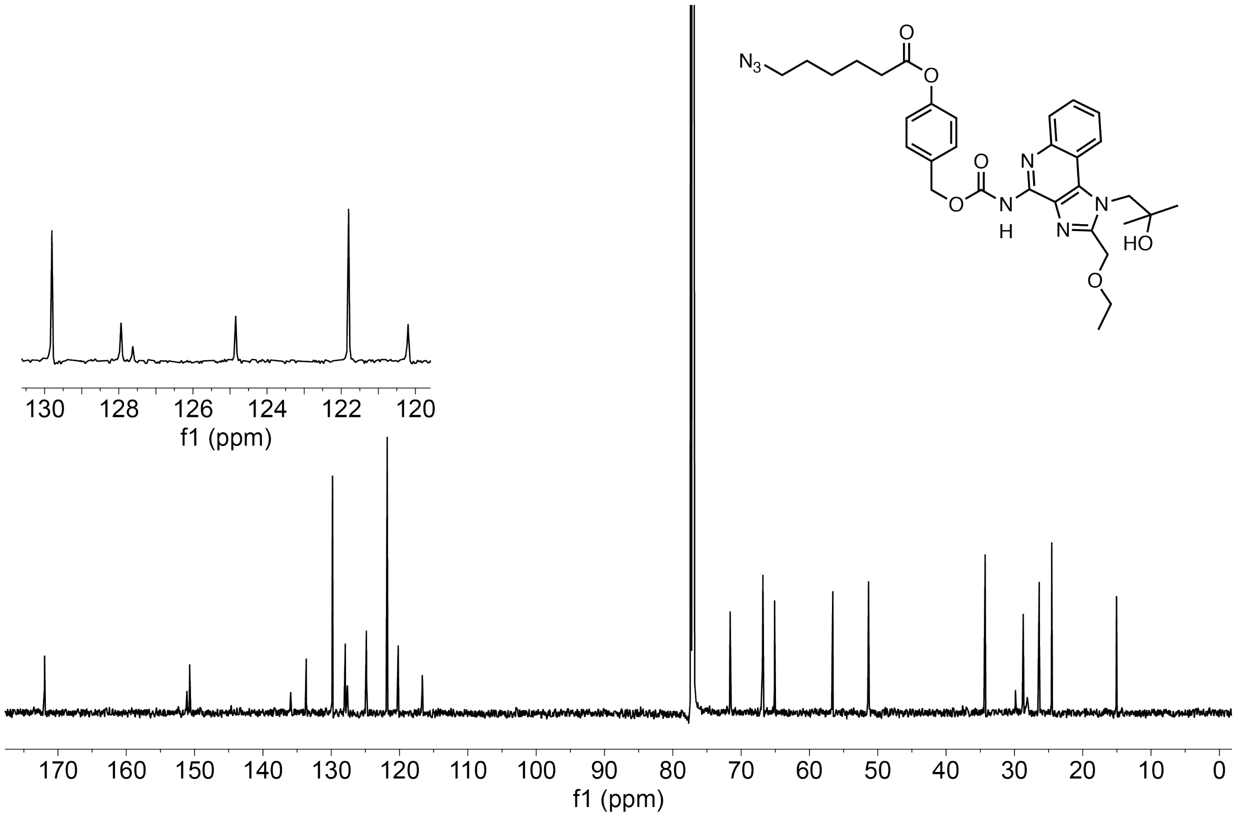

Fig. S6A-II. $^{13}C\{^{1}H\}$ NMR spectrum of **5a** ($CDCl_3$, 126 MHz, 25 °C).

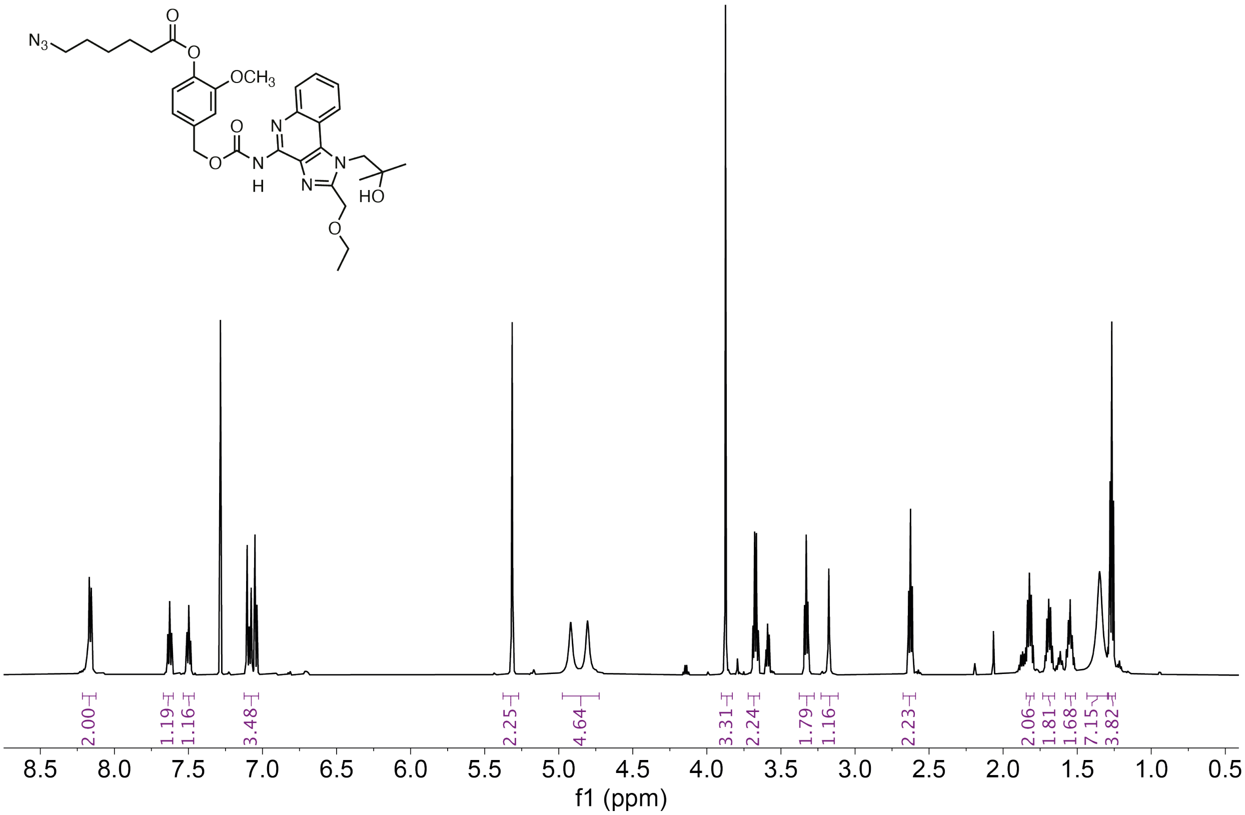

Fig. S6B-I. ^{1}H NMR spectrum of **5b** ($CDCl_3$, 500 MHz, 25 °C).

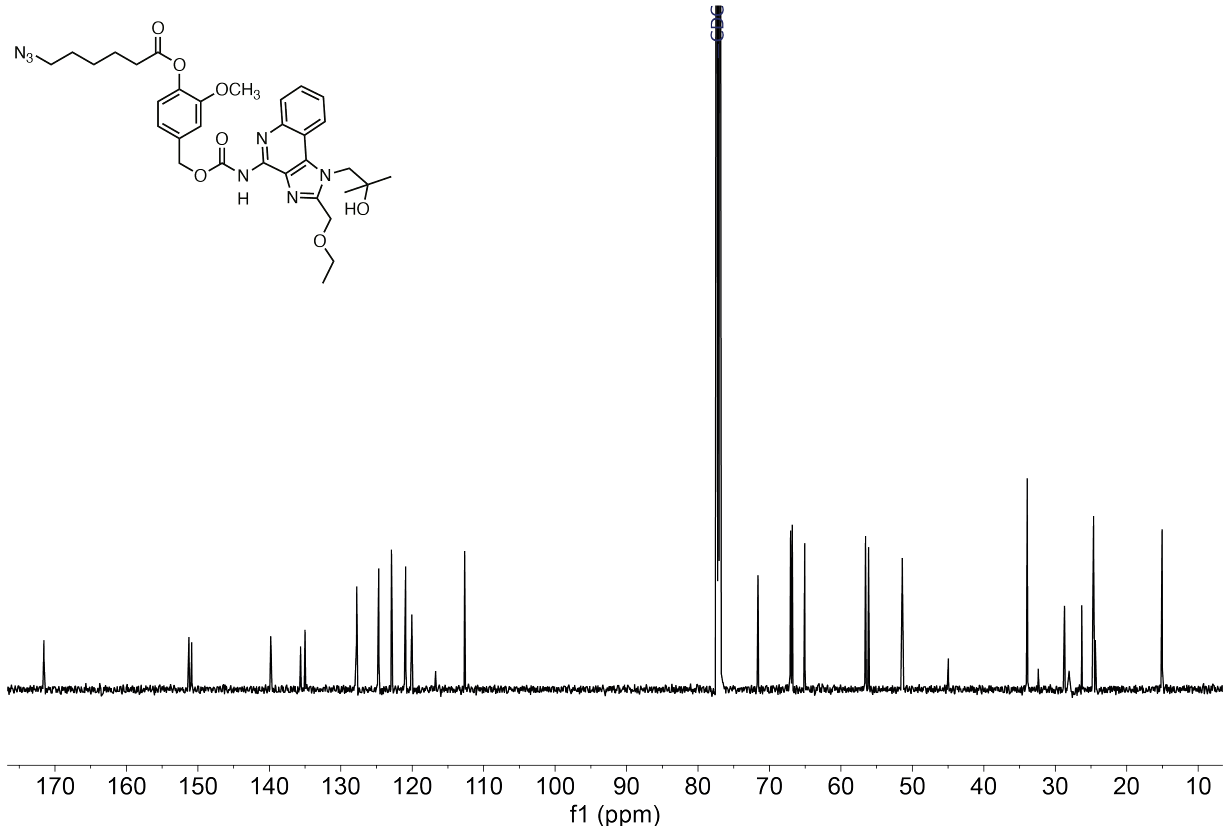

Fig. S6B-II. $^{13}C\{^{1}H\}$ NMR spectrum of **5b** ($CDCl_3$, 126 MHz, 25 °C).

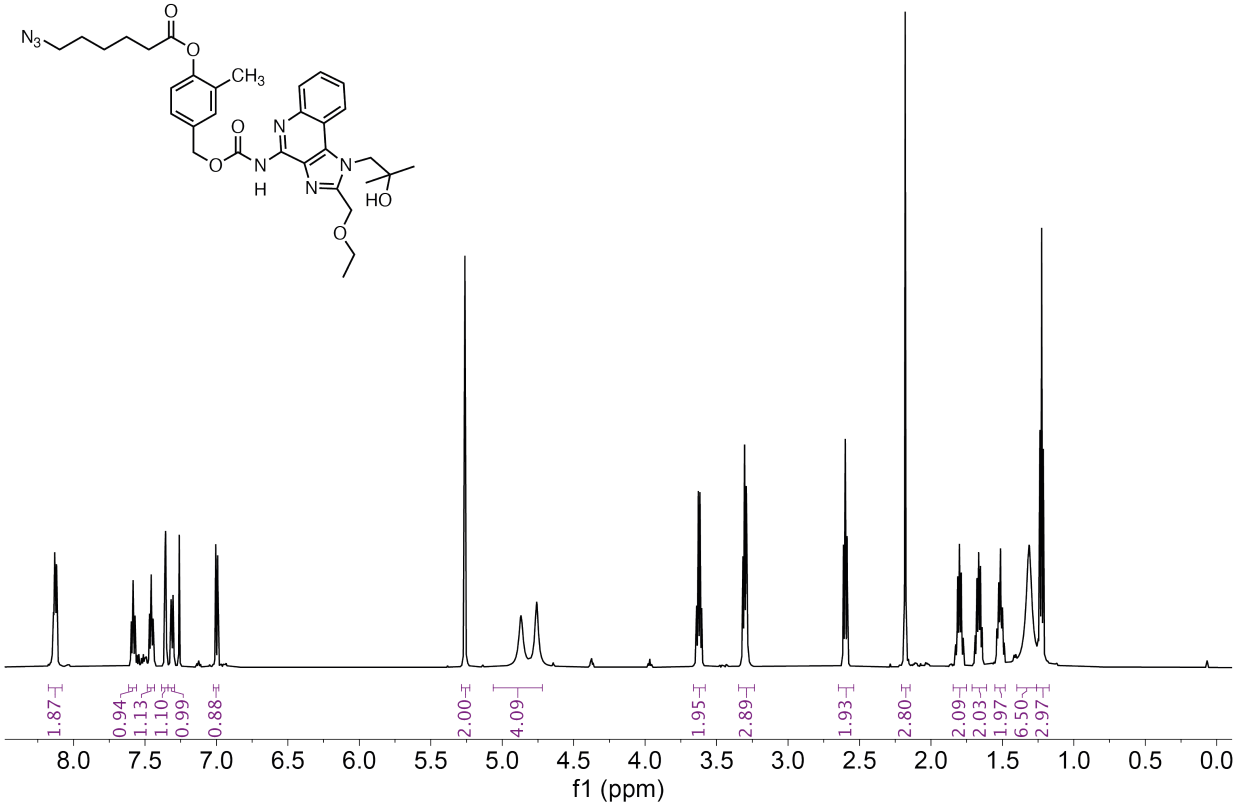

Fig. S6C-I. ^{1}H NMR spectrum of **5c** ($CDCl_3$, 500 MHz, 25 °C).

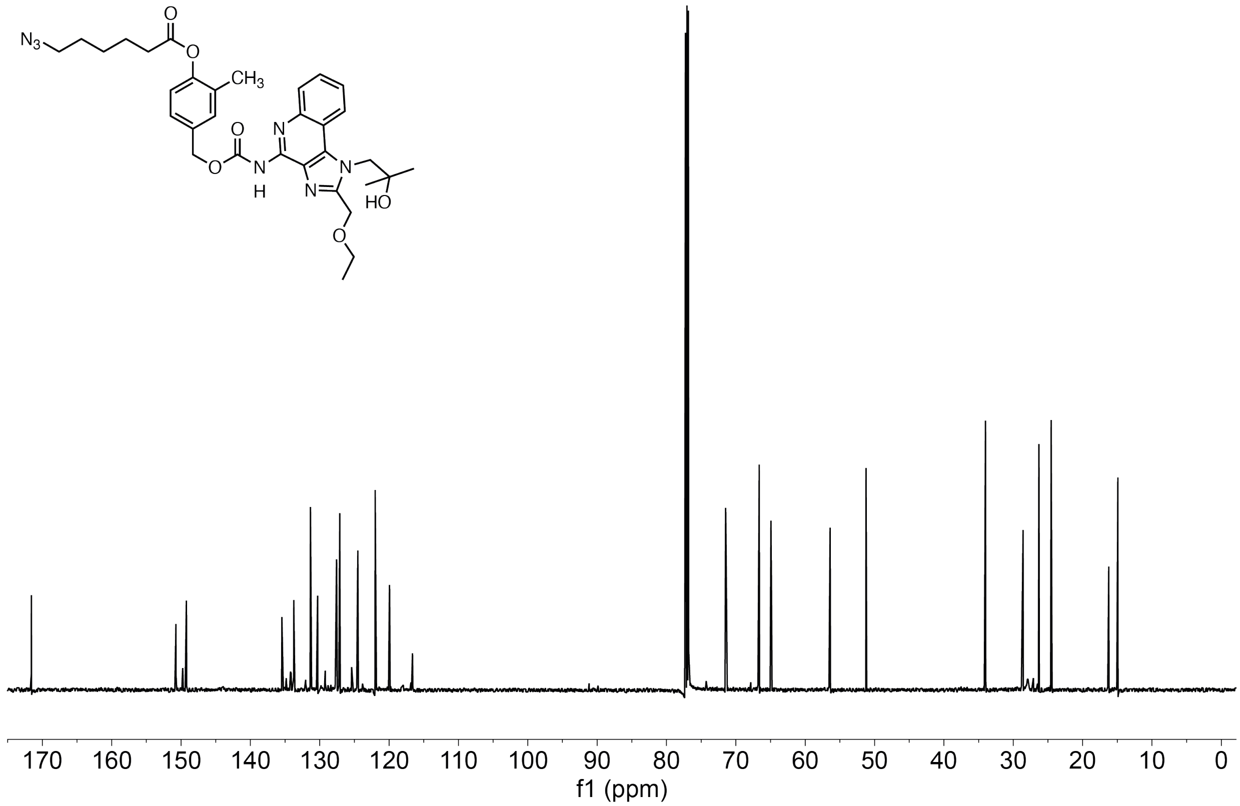

Fig. S6C-II. $^{13}C\{^{1}H\}$ NMR spectrum of **5c** ($CDCl_3$, 126 MHz, 25 °C).

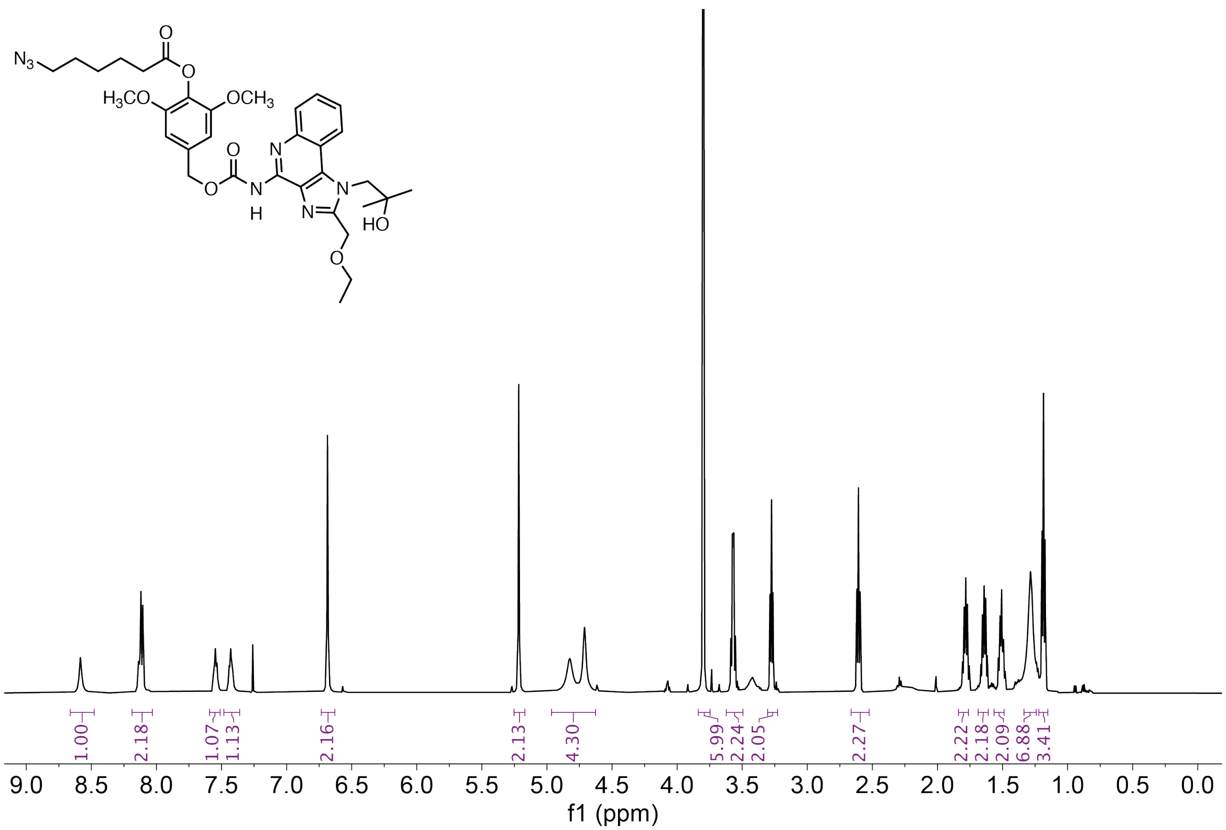

Fig. S6D-I. ^{1}H NMR spectrum of **5d** ($CDCl_3$, 500 MHz, 25 °C).

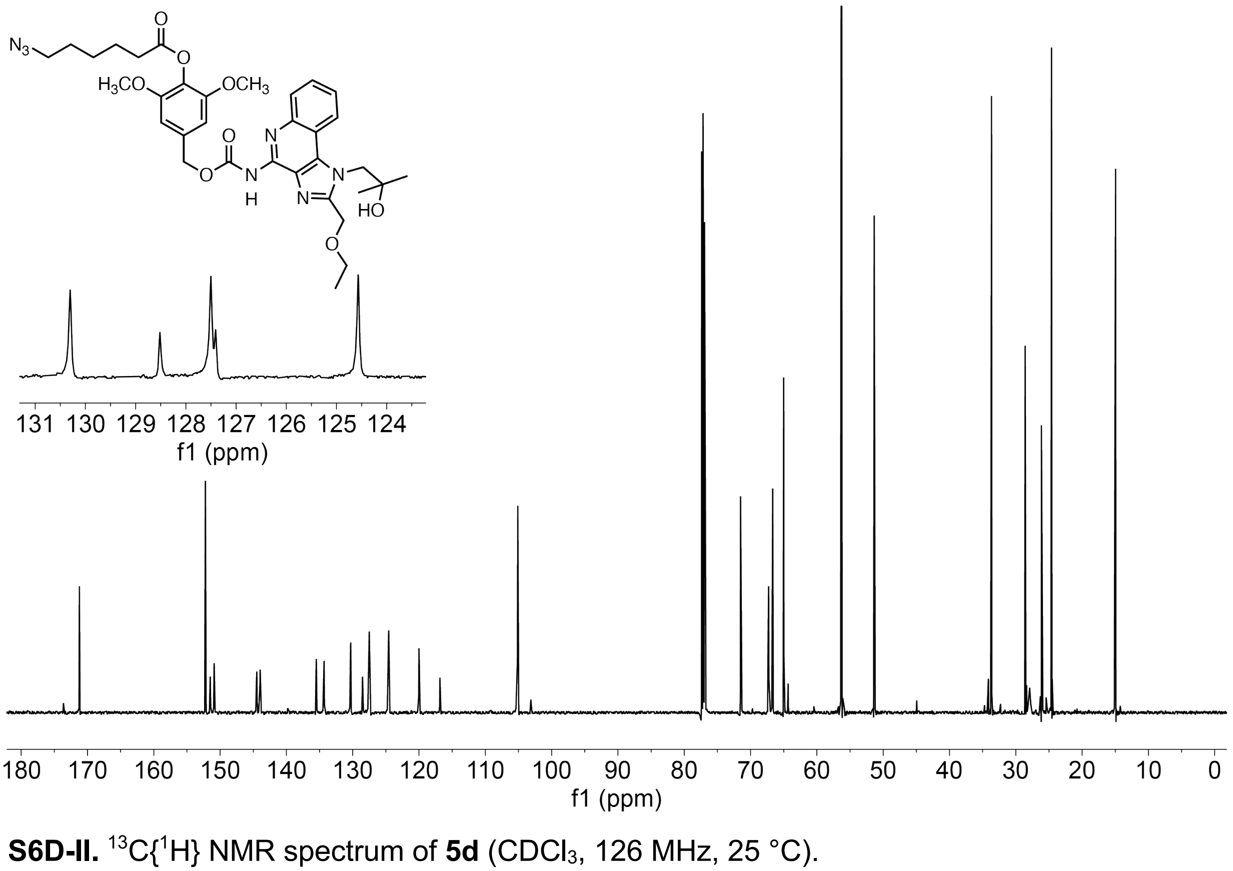

Fig. S6D-II. $^{13}C\{^{1}H\}$ NMR spectrum of **5d** ($CDCl_3$, 126 MHz, 25 °C).

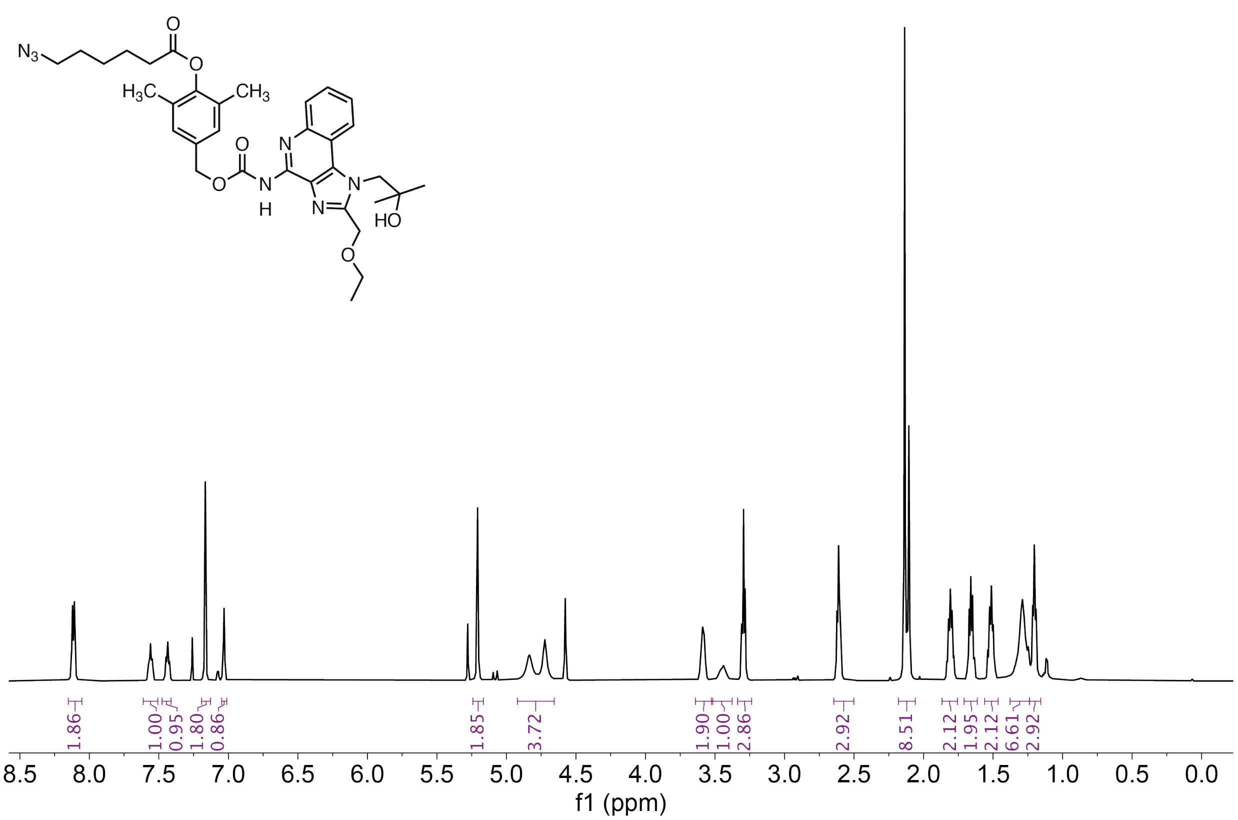

Fig. S6E-I. ^{1}H NMR spectrum of **5e** ($CDCl_3$, 500 MHz, 25 °C).

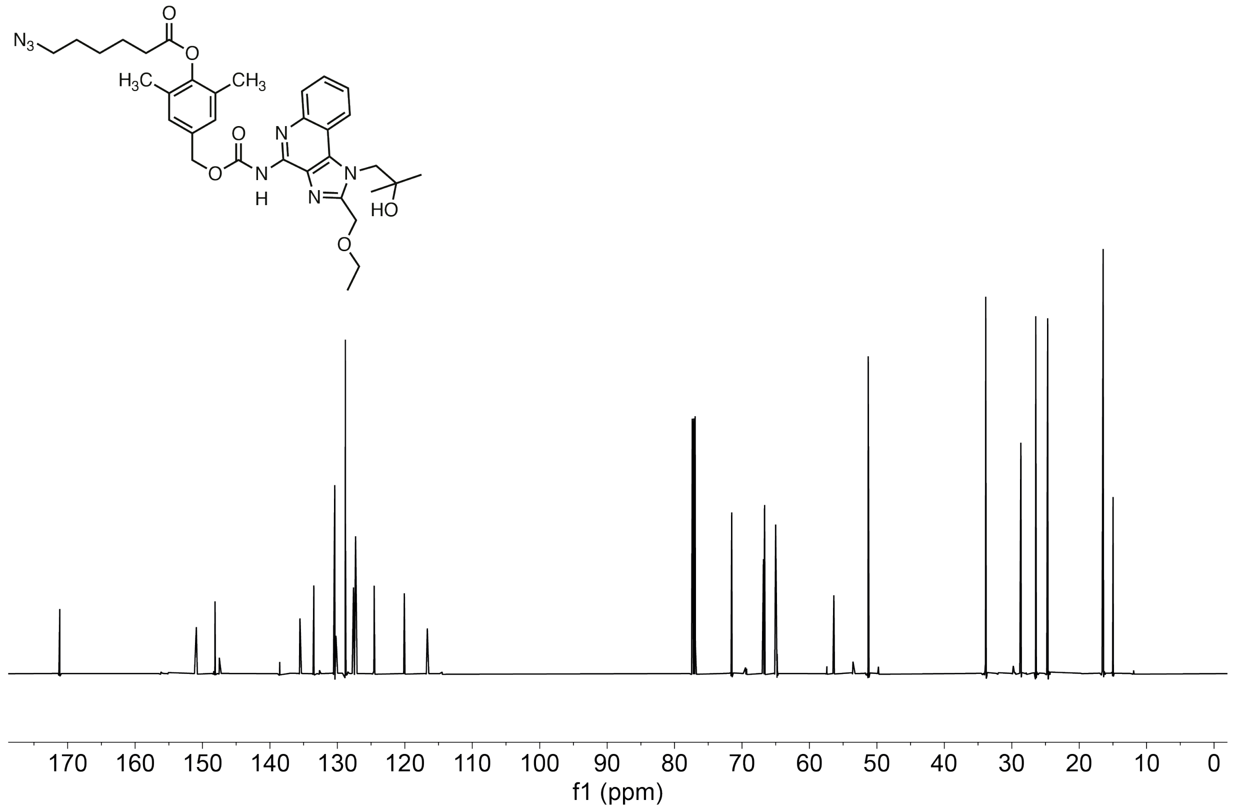

Fig. S6E-II. $^{13}C\{^{1}H\}$ NMR spectrum of **5e** ($CDCl_3$, 126 MHz, 25 °C).

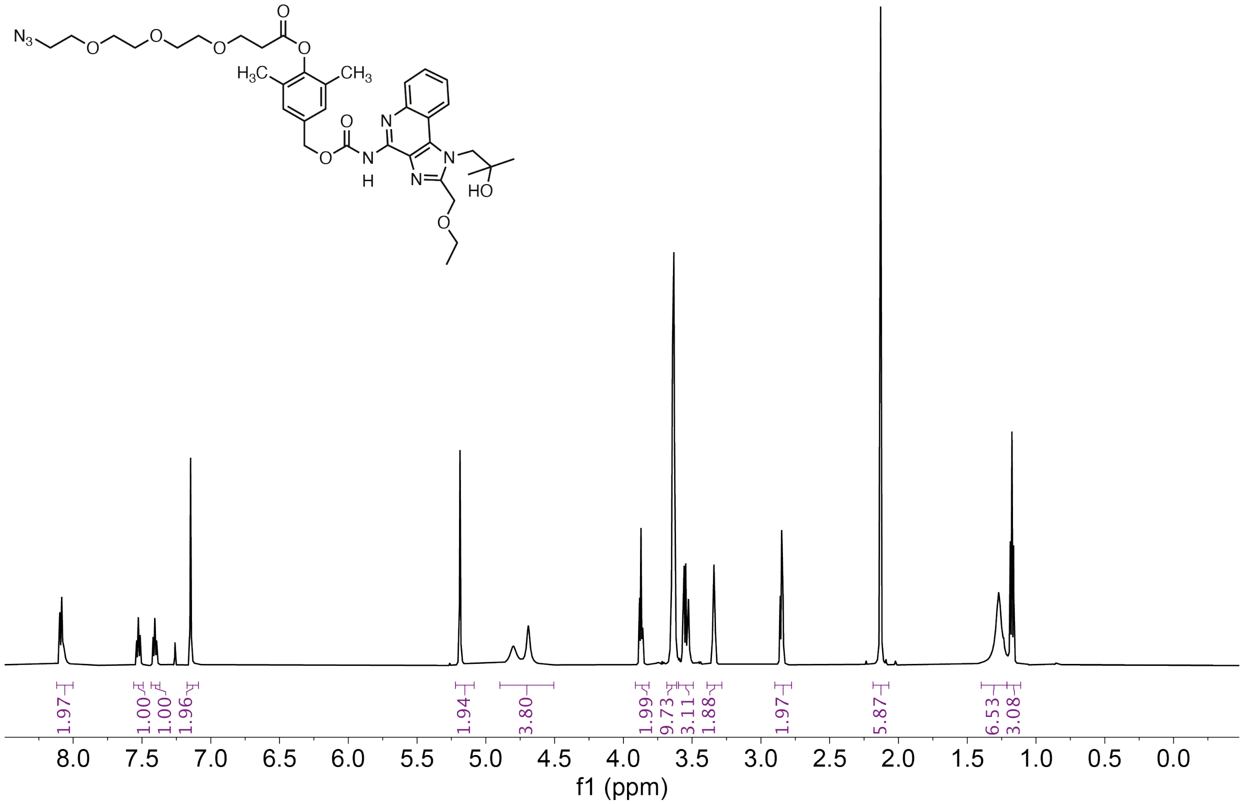

Fig. S6F-I. ^{1}H NMR spectrum of **5f** ($CDCl_3$, 500 MHz, 25 °C).

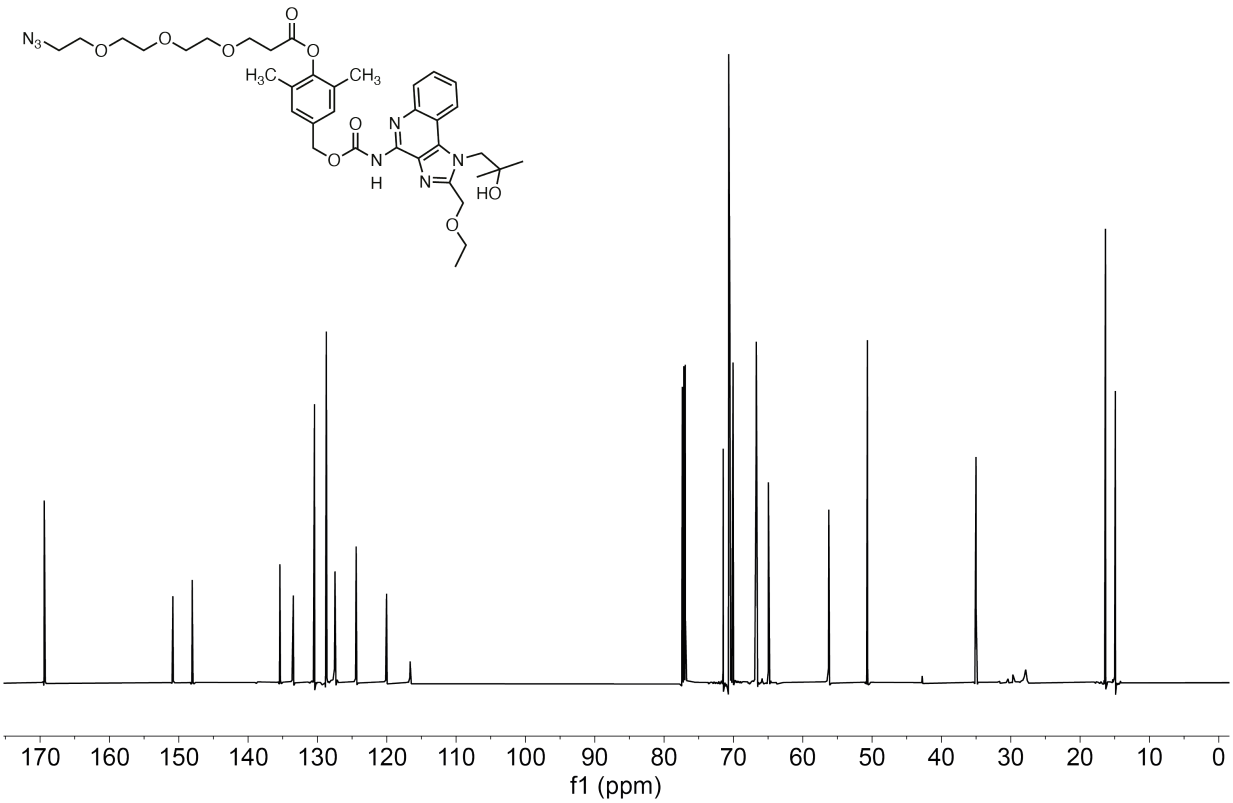

Fig. S6F-II. $^{13}C\{^{1}H\}$ NMR spectrum of **5f** ($CDCl_3$, 126 MHz, 25 °C).

Macromonomer

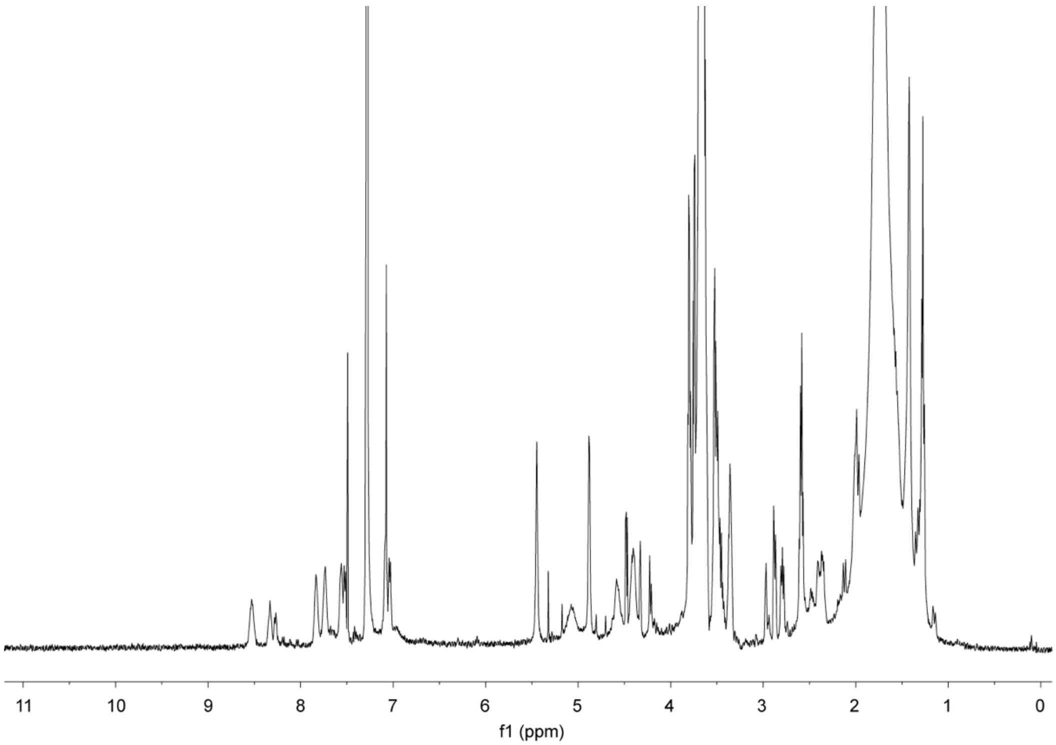

Fig. S7A. ^{1}H NMR spectrum of **6a** ($CDCl_3$, 500 MHz, 25 °C).

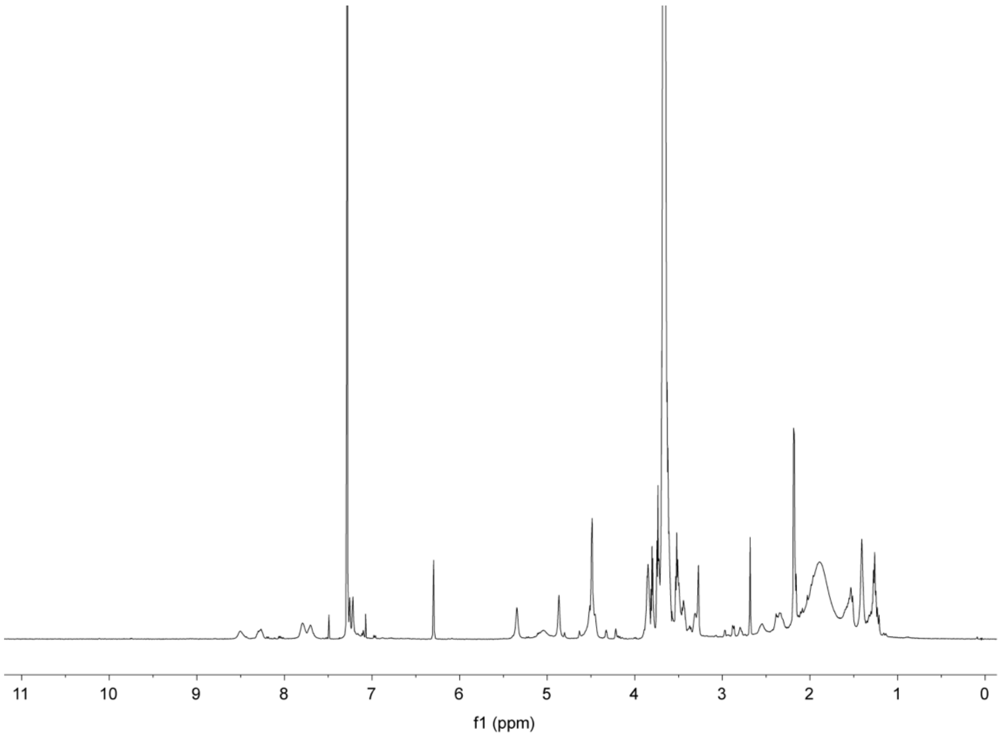

Fig. S7B. ^{1}H NMR spectrum of **6b** ($CDCl_3$, 500 MHz, 25 °C).

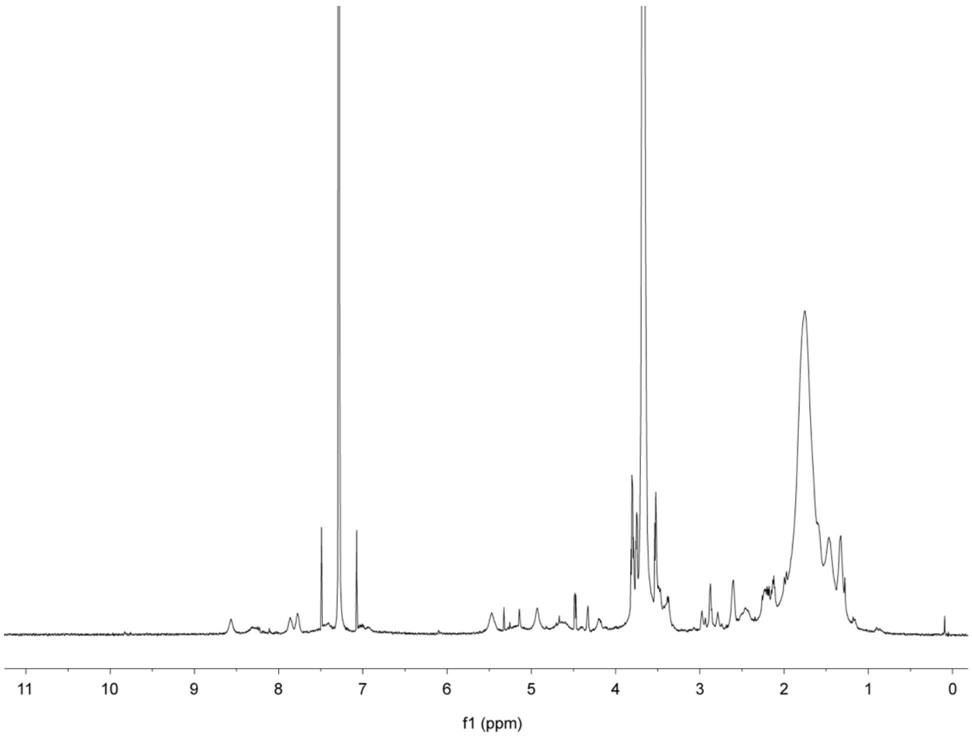

Fig. S7E. ^{1}H NMR spectrum of **6d** ($CDCl_3$, 500 MHz, 25 °C).

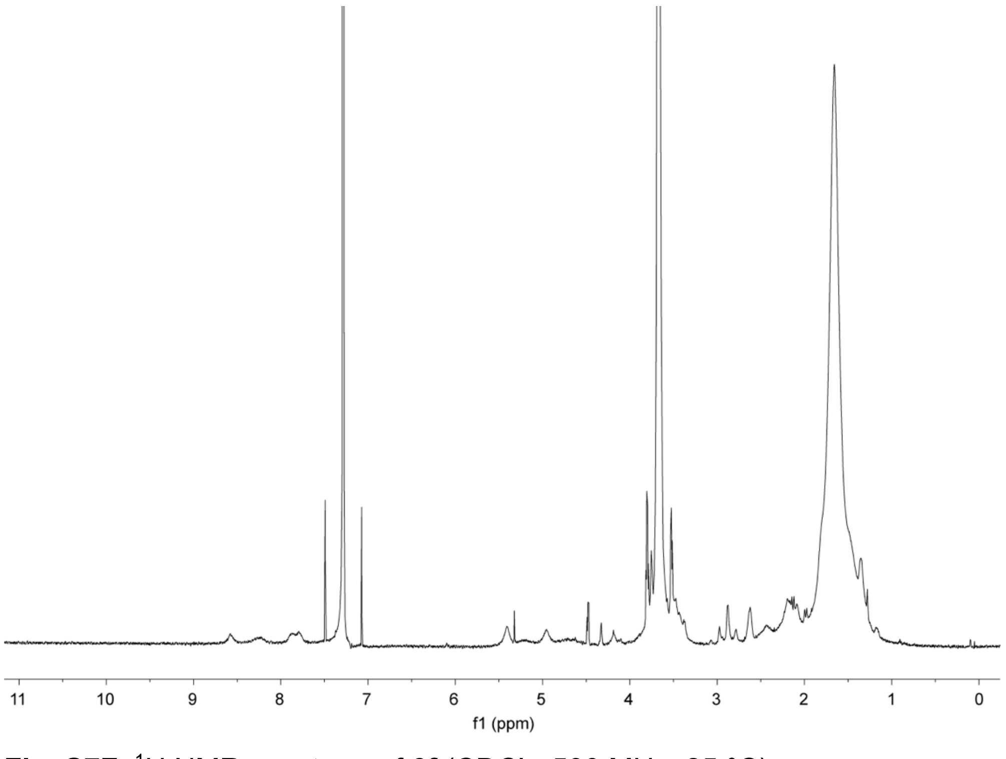

Fig. S7F. ^{1}H NMR spectrum of **6f** ($CDCl_3$, 500 MHz, 25 °C).

Cryo-transmission electron microscopy (Cryo-TEM) images and measurements

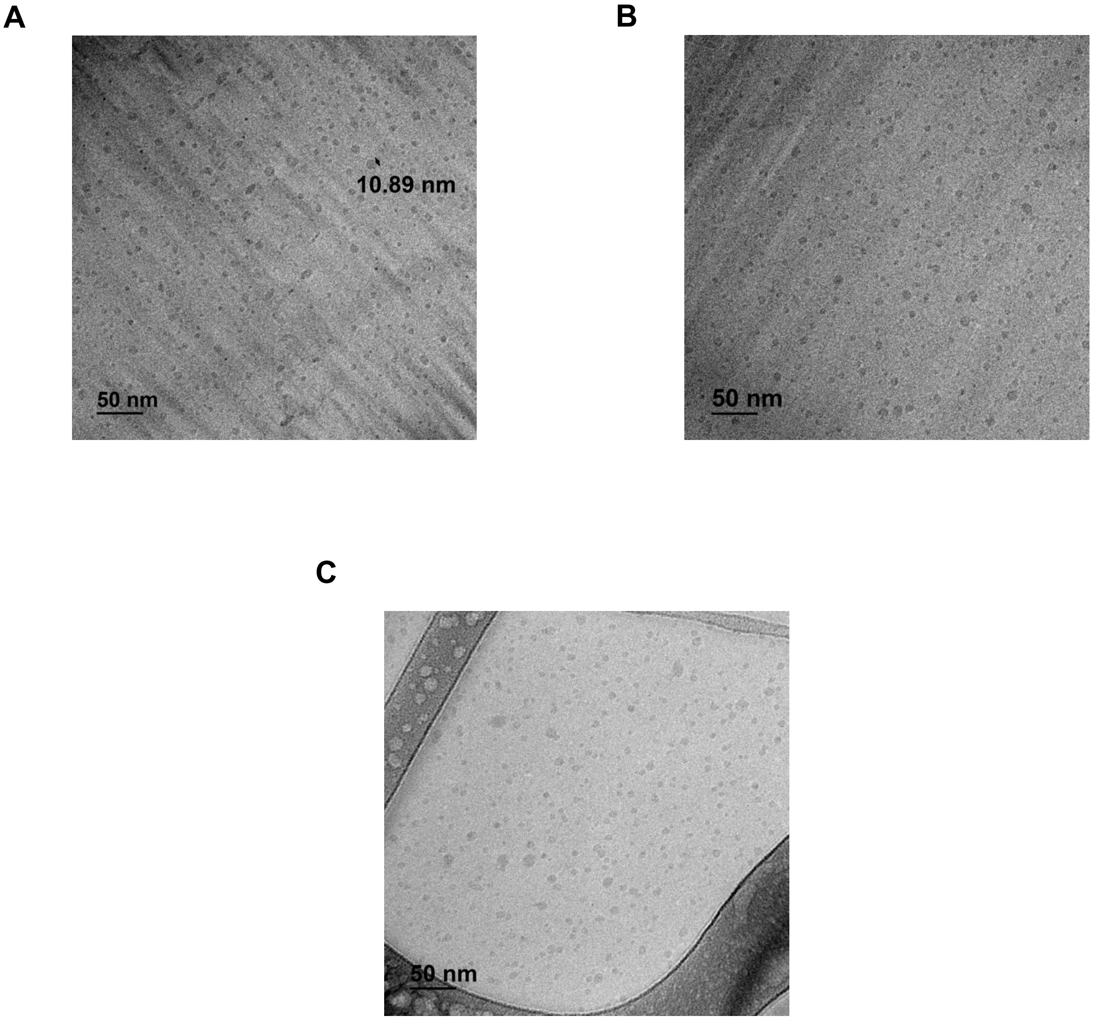

Fig. S8. (A-C) Additional Cryo-TEM images of R848-BPDs indicating particle sizes in the 10nm range.

Table. S1. Size measurements of cryo-TEM images of R848-BPDs

Fig 1E		S8A		S8B		S8C	
X	Y	X	Y	X	Y	X	Y
12.9	6.9	9.4	4.7	8.9	7.3	8.8	7.4
7.9	6.7	5.7	5.8	13	11.5	8.7	7.5
12.1	11.9	6.5	6.8	6.9	5.9	9.1	8
11.1	10.9	6.4	5.6	9.5	8.6	8.6	7.4
17.3	10.8	5.6	4.8	8.1	6.9	13.6	12.6
9.5	5.9	9.5	11.7	6.9	6.3	6.9	6
6.4	6.1	9.7	8.2	8.4	6.1	10.2	8.8
8.9	6.8	20	7.8	9.6	8.6	10.9	8.4
9.6	6.6	7.5	8.4	6.7	4.6	6.7	5.6
10.1	5.2	7.1	6.9	7.1	6.9	6.5	6.3
6.1	6	6.7	6.1	8.5	7.8	7.3	6.2
11.3	9.1	9	11	6.7	6.3	6.5	6.1
6.5	6.2	8.2	7.3	7.6	6.1	6.3	5.6
9.8	9.5	5.9	7.5	8.6	6.9	6.1	5.6
9.5	9	5.7	5	7.8	5.9	8.5	7.8
9.9	6.4	8.1	7.8	5.5	5.5	5.9	5.2
7.3	7	5.8	9.5	6.8	4.6	8.2	6.9
6.3	5.9	5.9	6.1	7.5	6.5	10.5	9.8
6.5	4	5.1	5.9	6.5	6.4	5.4	5.2
10	9.2	5.2	6.3	7.8	7.4	9.4	5.4
13.2	11.9	8.9	8.1	6.9	5.4	6.6	6.3
11.2	10.9	10.9	9.9	9.9	8.9	9.8	8.8
10.8	10.1	9	9.6	17.9	11.3	10.9	9
9.6	8.8	10.7	8.7	6.5	6.2	6.9	6.1
8.4	6.5	6.9	6.1	7.3	6.7	8.5	6.3
6.9	6.5	6.9	5.7	11.8	10.9	11.8	10.8
9.2	8	11.3	7.8	6.5	5.5	6.3	5.6
8.5	8.1	6.8	6.1	4.4	3.1	9.8	9.6
8.9	8.1	8.9	8	6.3	5.7	8.9	8.8
8	6.9	10.1	10.1	6.7	5.7	6.9	5.9
9.8	8.9	10.7	11.1	6.9	6.6	6.1	5.7
10	9	5.7	6.5	5.9	4.6	9.7	8.6
11.5	9.4	7.6	7.8	5.2	4.6	6.9	6.3
8.4	8.1	6.5	5.3	11.4	8.6	4.9	3.8
9.2	7.5	5.7	8.5	7.1	6	6.9	5.4
7.5	6.5	11.5	10.5	9.8	8.2	10.9	8.8
6.9	6.3	9.2	8.2	10.9	7.3	6.9	6.9
6.8	6.3	7.9	13.7	9.7	8.4	12.2	10.9
8.8	8.2	8	7.9	7.8	7.3	16.4	16.3
8.4	7.6	5.7	7.5	6.3	5.7	6.3	5.4
7.2	6.9	6.3	7.1	6.3	5.7	5	4.9
11.1	10.5	7.3	7.2	7.6	7.4	6.9	5.6
14.5	8.1	6.1	5.9	9.5	7.8	9.1	6.9
13.3	12.6	6.3	6.1	11.8	8.2	6.9	5.9
15.3	11.1	10.1	10.5	5.7	5	10.1	9.8
9.4	7.4	5.9	9.6	6.3	5.9	10.9	9.3
13.5	6.2	13.4	10.5	14	9.3	8.2	8
8.2	7.2	9.4	5.6	6.9	6.9	11.5	8.2
12	8.6	11.3	9.4	10.5	8.8	9.6	7.3
7.8	6.5	10.3	10.1	7.3	5.4	9.6	6.3
8.8	7.2	17.5	7.2	8	6.9	14.8	11.9
9.7	7.2	5.5	5.2	6.6	6.3	10.2	9.2
9.4	9.1	7.5	6.3	6.3	5.2	9.5	6.1
12	11.8	9	6.9	6.2	5	11.6	9
9.2	8.4	9.3	8	9.5	8.2	9.5	6.1
8.9	7.8	14.1	11	8.8	8.2	6.5	5.7
7.2	6.3	7.1	5.7	9	7.4	6.9	6.5
8.1	7.7	7.4	6.3	10.9	7.3	14.1	11.1
7.6	6.9	6.8	5.9	9.4	8	6.9	6.8
15.9	10.8	7.4	5.7	6.5	5.2	8.8	6.5
7.1	6.9	9.4	7.3	14.1	11.5	7.6	7.5
12.4	8.4	6.8	5	6.6	5	9.2	8.3
9.9	6.7	13.1	7.6	11.4	6.3	10	9
9.4	8	10.1	7.3	6.3	6.1	14.5	10.1
9	8.2	7.3	7.1	10.3	9.8	9	6.9
8.8	8.3	9	6.3	9.4	8.8	10.9	9.6
9.2	8.8	6.2	5	9	8.6	8.4	6.9
9.5	8.3	10.3	8.6	8.3	7.5	13.3	10
11.5	9.6	6.3	5.9	12.6	10.7	19.6	17.4
12.5	11.26	10.7	9	9.1	8.4	11.9	10.7
9.4	8.1	5.9	5.7	9.5	6.5	8.3	7.8
10.1	9.5	9.4	8.8	13.1	9.5	10.6	9.1
8.8	8.4	7.1	6.4	10.9	10.3	11	8.8
8.9	8.2	7.3	6.9	9	7.6	8.4	6.9
8.2	4.8	8.3	8.3	9.7	8.2	9.5	8.3
8.6	7.6	6.4	5.7	6.7	6.7	9.3	8.4
8.6	7.4	8.7	4	11.2	10.1	10.5	6.3
9.9	6.9	10.1	3.4	9.7	7.5	7	6.9
8.9	6.4	6.7	6.3	10.9	9.3	6.7	5.9
8.3	6.9	7.4	5.7	7	5.3	7.8	5.8
8.6	8.2	8.9	6.9	7.1	6.7	9.4	9
9.9	9.2	7.1	5.7	8.8	8	9.2	6.3
9	8.9	7.6	6.7	8.6	8.2	7.5	6.9
9.6	8.2	14	8.6	8.2	6.1	6.9	6
9	8.9	6.3	6	6.9	6.5	8	7
9	8.8	6.3	5.3	8.2	7	10.5	7.6
9.8	8.8	4.8	4.4	6.2	6	12.8	10.9
6.5	6.3	8.8	6.1	7.2	6.3	14.4	10.1
6.9	6.9	5.3	5	7.2	7	11.4	8
8.2	7.2	6.9	6.3	9.5	7.2	8.4	7.5
11.1	10.1	6.3	5	9.4	9.3	7.9	6.5
10.9	10.3	5.7	5.7	10.7	9.2	9.8	9.4
9.8	9.3	10.9	10.9	6.3	6.2	10.5	8.9
5.6	4.6	5.8	4.7	11.4	9.1	6.6	6
13.8	8.6	5.5	4.2	12.3	9	6.8	6.6
8.3	8.2	5.4	5.1	11.1	10.8	8.3	8
8.5	7.2	5.4	5.4	11.4	9.9	9.2	7.9
11.1	9.2	4.8	4.6	8.8	7.6	9.4	7.5
6.8	6.8	12.7	9.9	8.4	7.8	9.1	8
6.8	6	13.5	12	9.3	8.1	11.8	9.5
**Avg X**	**Avg Y**	**Avg X**	**Avg Y**	**Avg X**	**Avg Y**	**Avg X**	**Avg Y**
**9.483**	**8.0236**	**8.184**	**7.193**	**8.625**	**7.306**	**9.157**	**7.764**
**Avg Aspect Ratio**		**Avg Aspect Ratio**		**Avg Aspect Ratio**		**Avg Aspect Ratio**	
**1.1965089**		**1.17030139**		**1.18438108**		**1.18571771**	

Release rate measurements *in-vitro*

Table. S2. Release rate half-life calculations for R848-BPDs

	Ln(1-x)	Note: x = %R848 released (as measured by LC-MS)				
**Time (in hours)**	**PE**	**tDMPE**	**MPE**	**dMPE**	**OMPE**	**dOMPE**
0	0	0	0	0	0	0
3	-0.0356956	-0.0315685	-0.0133464	-0.0078428	-0.0133464	-0.0078428
30	-0.2372693	-0.1014046	-0.0623598	-0.0270543	-0.0623599	-0.0301413
45	-0.3506816	-0.1817966	-0.1020843	-0.0445978	-0.1132209	-0.048789
50	-0.3740936	-0.1966971	-0.11117	-0.0473244	-0.1337753	-0.0610481
145	-1.0645457	-0.5807622	-0.3559668	-0.1255632	-0.4612486	-0.1800841
190	-1.3602343	-0.7519959	-0.4427496	-0.1606751	-0.5757767	-0.2464522
350	-2.1548197	-1.2136777	-0.6930088	-0.2781654	-0.9161177	-0.3795465
480	-3.5446791	-1.794617	-0.9412233	-0.3457428	-1.0412233	-0.3457428
500	-3.8419306	-1.8888523	-0.9712003	-0.3633757	-1.0926156	-0.4856044
525	-3.7678227	-1.9731922	-1.1434688	-0.3690091	-1.1434688	-0.5090091
675	-5.154117	-2.4826632	-1.2243868	-0.4452877	-1.5420695	-0.5609969

$T_{1/2}$	PE	tdMPE	OMPE	MPE	dOMPE	dMPE
**hours**	94.9516686	187.337076	301.368339	364.814306	866.433976	990.210258
**days**	**3.95631952**	**7.80571149**	**12.5570141**	**15.2005961**	**36.1014157**	**41.2587607**

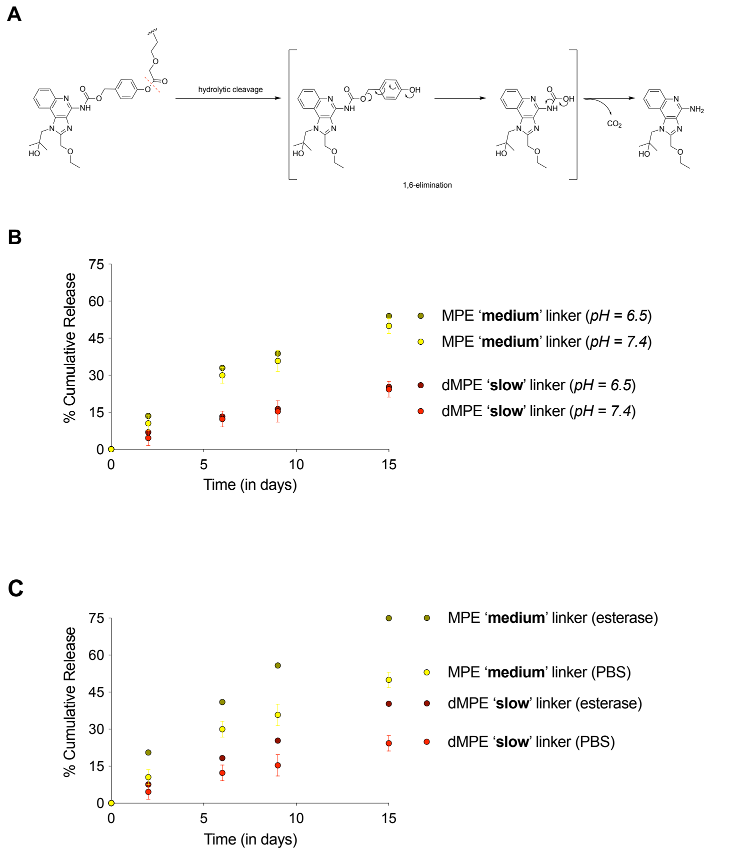

Fig. S9. *In-vitro* release studies. (A) Mechanism for release of free R848 from R848-BPDs. **(B)** Release studies carried out at pH = 6.5. **(C)** release studies carried out in the presence of a model esterase (T. lanuginosus lipase, 800 U/ml added at t=0)

In-vitro assays

A

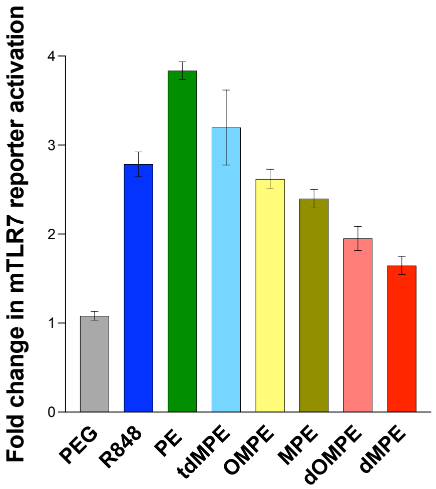

B

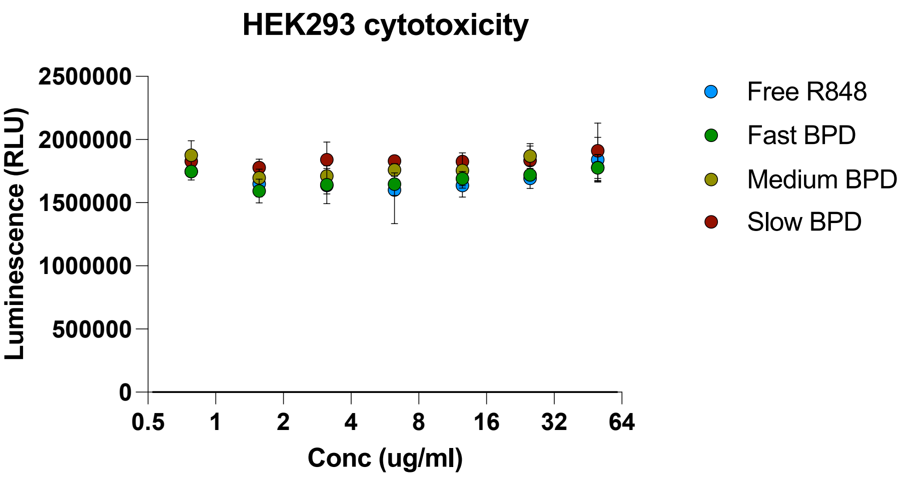

Fig. S10. *In-vitro* analysis of (A) immune activity in mouse TLR7 HEK293 reporter cells. Cells were seeded at 3 × 10^4 cells/96-well for 24 h then treated with R848, R848-BPDs, PBS or DMSO for 48 h. To assess innate immune stimulation, secreted alkaline phosphatase (SEAP) was measured from supernatants using QuantiBlue reagent to confirm dose-dependent activation of the target TLR receptor. **(B) Cytotoxicity with HEK293 cells via Cell-Titer Glo.** HEK293 cells were incubated with R848, R848-BPDs, PBS or DMSO for 48 h. Cell viability was measured via CellTiterGlo for the respective groups. Data are representative of at least two independent experiments with n=3 technical replicates per group unless otherwise indicated.

In vivo maximum tolerable dose data for R848-BPDs

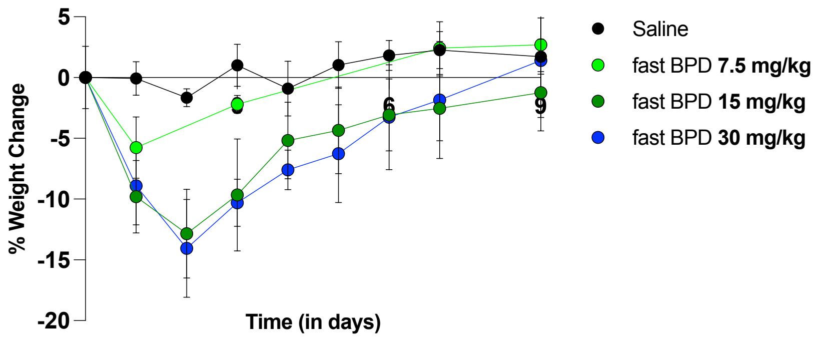

Fig. S11. Weight loss data for fast (PE) BPD when injected i.v. at different concentrations (7.5 mg/kg, 15 mg/kg, & 30 mg/kg). 7.5 mg/kg was determined as the maximum tolerable dose for the fast BPD.

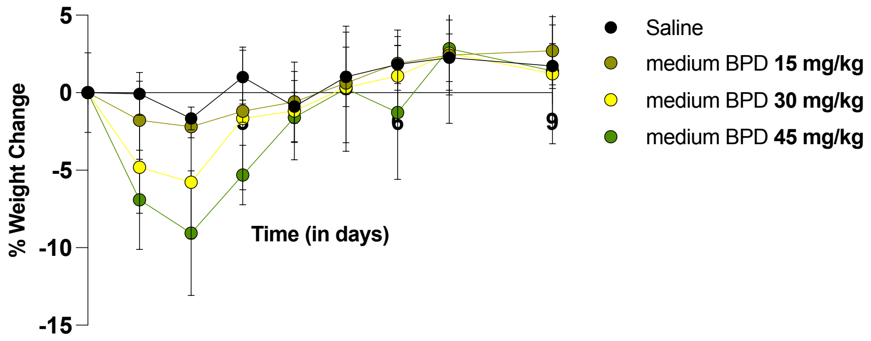

Fig. S12. Weight loss data for medium (MPE) BPD when injected i.v. at different concentrations (15 mg/kg, 30 mg/kg and 45 mg/kg). 30 mg/kg was determined as the maximum tolerable dose for the medium BPD.

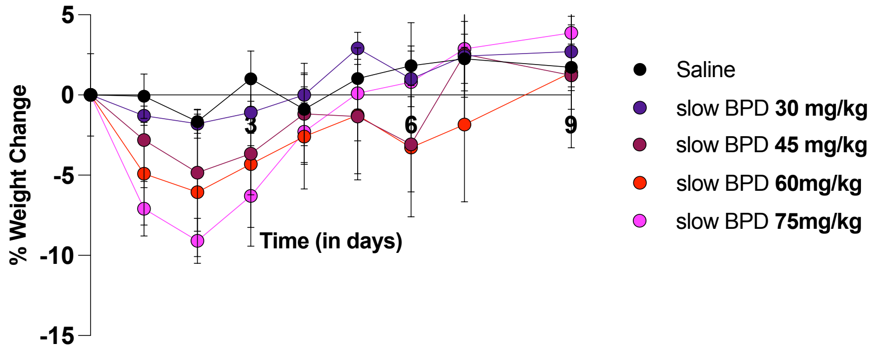

Fig. S13. Weight loss data for slow (dMPE) BPD when injected i.v. at different concentrations (30 mg/kg, 45 mg/kg, 60 mg/kg and 75 mg/kg). 60 mg/kg was determined as the maximum tolerable dose for the medium BPD.

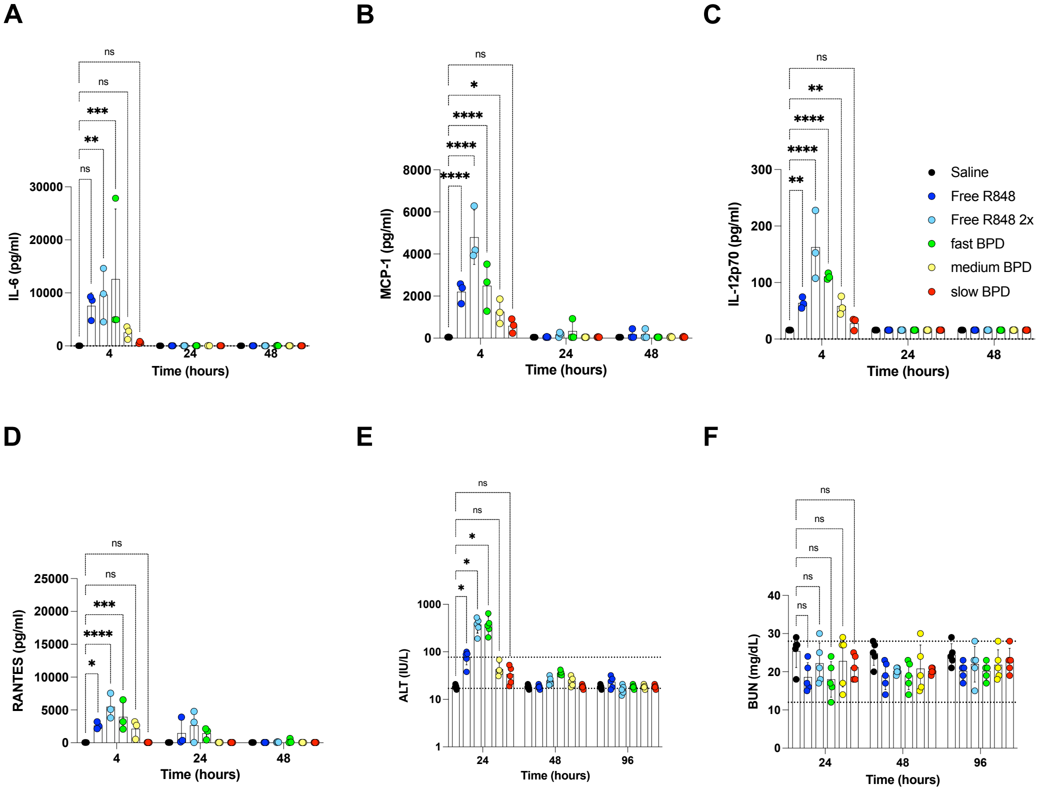

Fig. S14. Additional toxicity analysis for R848-BPDs. Saline, R848 (at 7.5 mg/kg and 15mg/kg) and R848-BPDs (7.5 mg/kg) when injected *i.v.* via retro-orbital injection. Shown are **(A-D)** serum concentrations of inflammatory cytokines and chemokines measured using a cytokine bead array as a function of time, **(E)** liver enzyme alanine aminotransferase and **(F)** blood urea nitrogen with the normal ranges indicated by dashed horizontal lines.

Table. S3. One phase decay curve fitting of plasma concentrations for Free R848, Medium BPDs, & Slow BPDs in non-tumor bearing C57BL/6 mice

	Y0	Plateau	K	Half Life (hrs)	Tau	AUC
Free R848	1352	7.219	1.484	0.467	0.6738	525.1
Medium BPDs	195.1	66.18	0.101	6.843	9.873	110.4
Slow BPDs	118.5	-216.0	0.008	82.92	119.6	67.87

In vivo multi-dose weight loss data for R848-BPDs

A

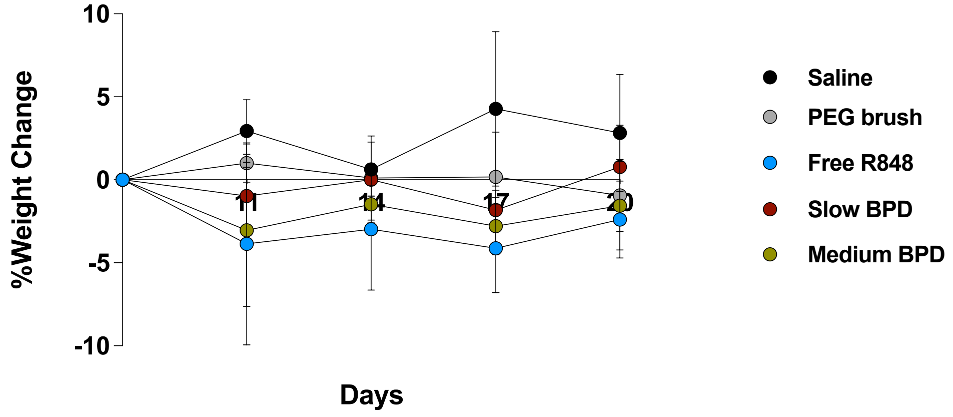

B

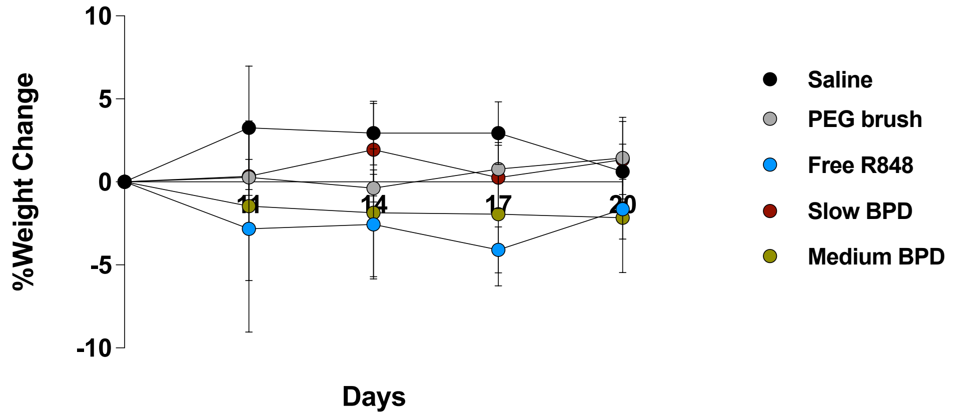

Fig. S15. **(A)** Weight loss curves for C57BL/6 mice bearing syngeneic MC38 colon carcinoma tumors injected retro-orbitally with R848-BPDs (medium and slow) along with controls. **(B)** Weight loss curves for BALB/c mice bearing syngeneic CT26 colon carcinoma tumors injected retro-orbitally with R848-BPDs (medium and slow) along with controls.

Single-cell RNA-sequencing (scRNA-seq)

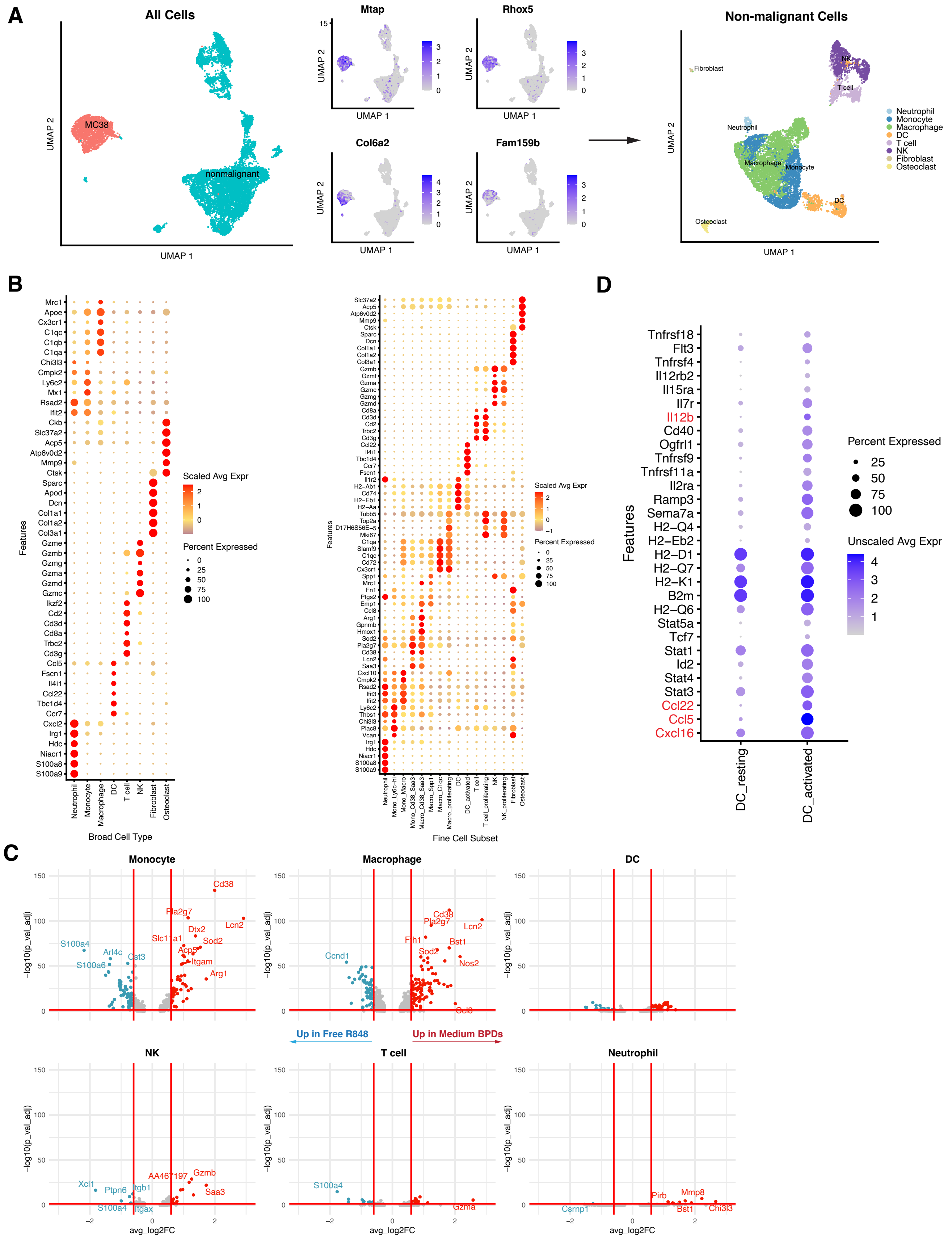

Fig. S16. Single-cell RNA-sequencing (scRNA-seq) of MC38 tumors treated with saline, free R848 and medium R848-BPDs (Medium BPD). (**A**) UMAP visualization of all 9,832 cells, including MC38 tumor cells and markers used to identify MC38 tumor cells (*Mtap, Rhox5, Col6a2, Fam159b).* After removing tumor cells and reclustering 8,017 non-malignant cells, 8 broad cell types were identified in the UMAP visualization. (**B**) RNA expression of top markers used to identify broad non-malignant cell types (left) and top differentially expressed genes in each fine cell subsets. (**C**) Volcano plots of differentially expressed genes in each broad immune cell type when comparing the Medium BPD condition to Free R848 condition. Genes upregulated in the Medium BPD condition is labeled in red and genes upregulated in Free R848 condition is labeled in blue. (**D**) Differentially expressed chemokines (colored in red), cytokines (colored in red), transcription factors, MHCs, and membrane signaling molecules in activated versus resting DCs.

Gating strategy for immunophenotyping

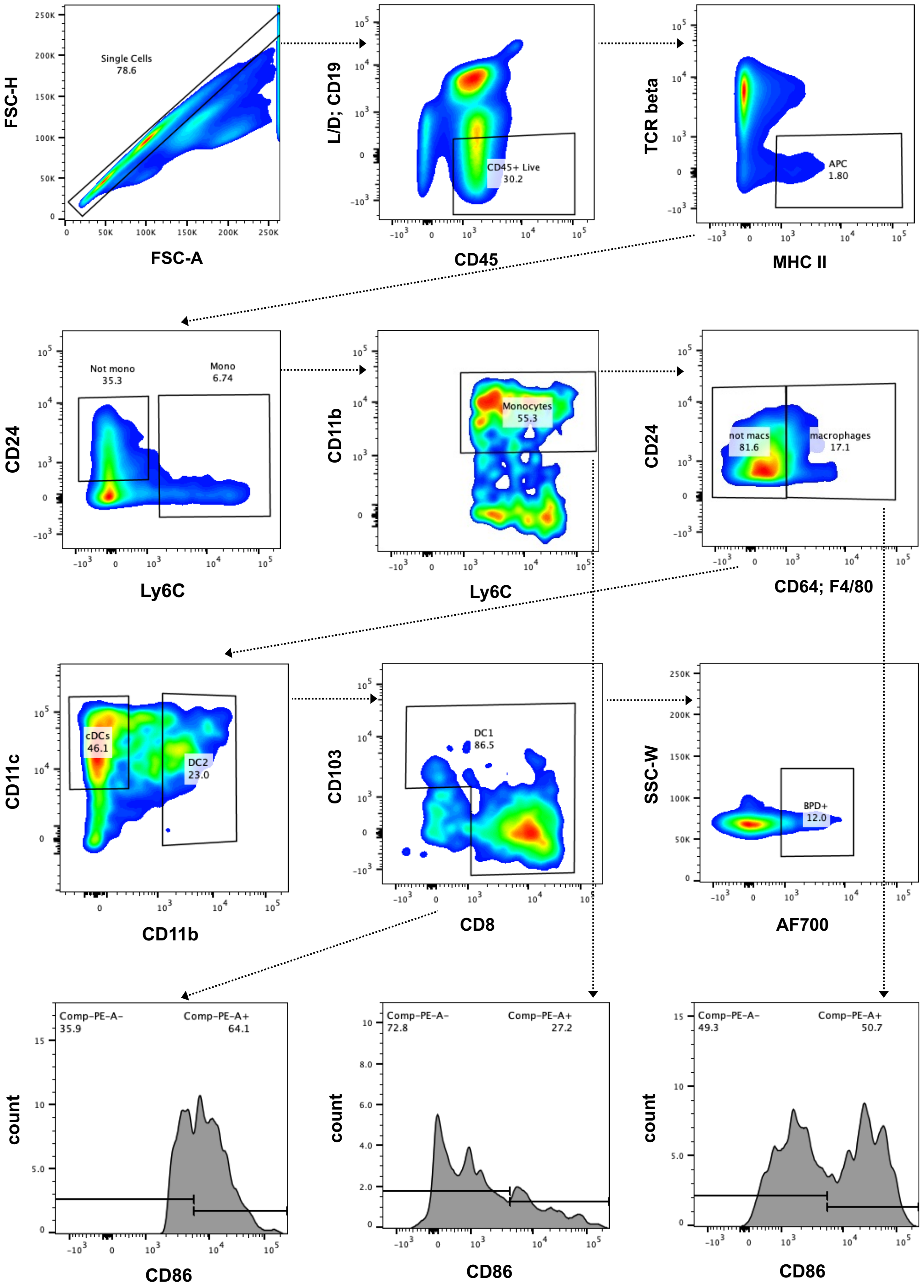

Fig. S17. Representative gating for myeloid analysis of tumor-draining lymph nodes as seen in Fig 7A.

Cell depletion studies

A

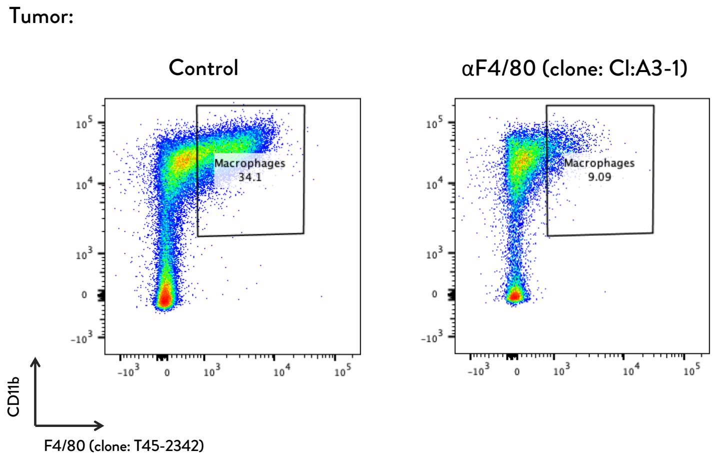

B

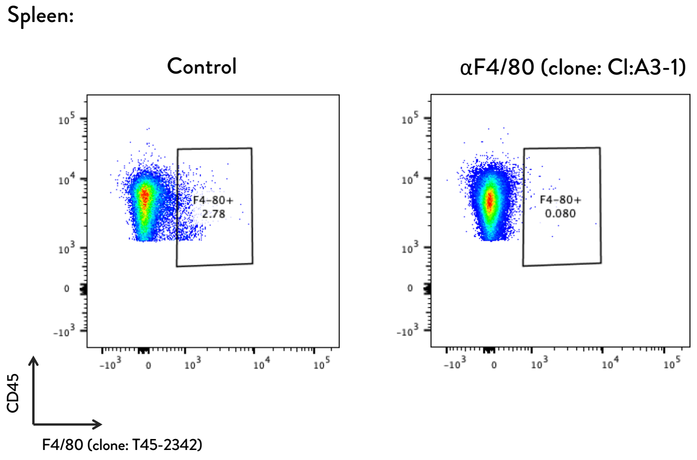

Fig. S18. Antibody-mediated depletions in (A) tumors and (B) spleen. Mice (n = 3) bearing MC38 tumors were administered indicated depleting antibodies beginning 1d before tumor inoculation and every 3d after. Tumors and spleens were collected at day 12 and analyzed for depletion efficiency via flow cytometry.

Table. S4. Statistics for scRNA-seq cell compositional analysis and differential gene analysis. Table for Fig. 6B: statistics for compositional change of cell subsets across 3 conditions; Table for Supplementary Fig. 16C: statistics for differential gene expression analysis within each immune cell type comparing Medium BPD to Free R848 condition.
